# Candidate methylation sites associated with endocrine therapy resistance in ER+/HER2- breast cancer

**DOI:** 10.1186/s12885-020-07100-z

**Published:** 2020-07-19

**Authors:** Maryam Soleimani Dodaran, Simone Borgoni, Emre Sofyalı, Pernette J. Verschure, Stefan Wiemann, Perry D. Moerland, Antoine H. C. van Kampen

**Affiliations:** 1 Bioinformatics Laboratory, Department of Clinical Epidemiology, Biostatistics, and Bioinformatics, Amsterdam Public Health research institute, Amsterdam UMC, University of Amsterdam, Meibergdreef 9, Amsterdam, AZ 1105 The Netherlands; 2 Biosystems Data Analysis, Swammerdam Institute for Life Sciences, University of Amsterdam, Science Park 904, Amsterdam, 1098 XH The Netherlands; 3 Division of Molecular Genome Analysis, German Cancer Research Center (DKFZ), Im Neuenheimer Feld 580, 69120 Heidelberg, Germany; 4 Faculty of Biosciences, University Heidelberg, 69120 Heidelberg, Germany; 5 Synthetic Systems Biology and Nuclear Organization, Swammerdam Institute for Life Sciences, University of Amsterdam, Science Park 904, Amsterdam, 1098 XH The Netherlands

**Keywords:** Breast cancer, DNA methylation, Endocrine therapy, Resistance, Survival, T47D

## Abstract

**Background:**

Estrogen receptor (ER) positive breast cancer is often effectively treated with drugs that inhibit ER signaling, i.e., tamoxifen (TAM) and aromatase inhibitors (AIs). However, 30% of ER+ breast cancer patients develop resistance to therapy leading to tumour recurrence. Changes in the methylation profile have been implicated as one of the mechanisms through which therapy resistance develops. Therefore, we aimed to identify methylation loci associated with endocrine therapy resistance.

**Methods:**

We used genome-wide DNA methylation profiles of primary ER+/HER2- tumours from The Cancer Genome Atlas in combination with curated data on survival and treatment to predict development of endocrine resistance. Association of individual DNA methylation markers with survival was assessed using Cox proportional hazards models in a cohort of ER+/HER2- tumours (*N* = 552) and two sub-cohorts corresponding to the endocrine treatment (AI or TAM) that patients received (*N* = 210 and *N* = 172, respectively). We also identified multivariable methylation signatures associated with survival using Cox proportional hazards models with elastic net regularization. Individual markers and multivariable signatures were compared with DNA methylation profiles generated in a time course experiment using the T47D ER+ breast cancer cell line treated with tamoxifen or deprived from estrogen.

**Results:**

We identified 134, 5 and 1 CpGs for which DNA methylation is significantly associated with survival in the ER+/HER2-, TAM and AI cohorts respectively. Multi-locus signatures consisted of 203, 36 and 178 CpGs and showed a large overlap with the corresponding single-locus signatures. The methylation signatures were associated with survival independently of tumour stage, age, AI treatment, and luminal status. The single-locus signature for the TAM cohort was conserved among the loci that were differentially methylated in endocrine-resistant T47D cells. Similarly, multi-locus signatures for the ER+/HER2- and AI cohorts were conserved in endocrine-resistant T47D cells. Also at the gene set level, several sets related to endocrine therapy and resistance were enriched in both survival and T47D signatures.

**Conclusions:**

We identified individual and multivariable DNA methylation markers associated with therapy resistance independently of luminal status. Our results suggest that these markers identified from primary tumours prior to endocrine treatment are associated with development of endocrine resistance.

## Background

Breast cancer (BRCA) is among the most common cancers diagnosed in women in Europe where it also is the third cause of cancer death after lung and colorectal cancer [1]. Approximately 75% of breast tumours is characterized by the expression of estrogen receptor alpha (ERα), encoded by the estrogen receptor 1 (*ESR1*) gene. These tumours require estrogen signals for continued growth and, consequently, patients generally receive endocrine treatment to inhibit ER signalling [2]. Endocrine treatment comprises selective estrogen receptor modulators including tamoxifen, selective estrogen receptor down-regulators including fulvestrant, and AIs (e.g., anastrozole, letrozole and exemestane) that inhibit the production of estrogen from androgen. Unfortunately, resistance to endocrine therapy (ET) develops in approximately 30% of ER+ BRCA patients resulting in recurrence of the tumour [3]. Despite many efforts the precise mechanisms leading to acquired treatment resistance remain mostly unknown and, therefore, therapies to prevent or revert resistance are currently lacking. Therefore, the identification of biomarkers, including epigenetic markers, that can predict endocrine resistance are considered of great value for patient stratification prior to ET [4].

In general, BRCA development, progression, and (endocrine) drug resistance result from the cumulative burden of genetic and epigenetic changes. Moreover, post-transcriptional and post-translational modifications are likely to contribute as well [5–7]. The association of epigenetic changes with tumour characteristics, subtypes, prognosis, and treatment outcome is only partially characterized [8]. Epigenetic changes have been shown to drive resistance acquisition (RA) through their effect on gene expression and/or chromosomal stability [9]. For example, using RNA-seq and ChIP-seq analysis of the acetylation of lysine 27 on histone 3 (H3K27ac), an established active enhancer marker, revealed that epigenetic activation of the cholesterol biosynthesis pathway causes activation of ERα resulting in resistance [10]. DNA methylation is also perturbed during BRCA development and may largely affect gene expression [4, 11]. Since DNA methylation has also been shown to be altered in endocrine resistant tumours [12], the identification of methylation markers for disease diagnosis, prognosis, and treatment outcome is receiving increased attention. Moreover, BRCA treatment might benefit from the regulation of methylation activity by using DNA methyltransferase inhibitors [4]. Treatment with the DNA methyltransferase inhibitor 5-aza-2′ deoxycytidine caused a significant reduction in promoter methylation and a concurrent increase in expression of the gene *ZNF350* that encodes a DNA damage response protein, and of *MAGED1* which is a tumour antigen and putative regulator of P53, suggesting that a methylation-targeted therapy might be beneficial [13]. However, current inhibitors have weak stability, lack specificity for cancer cells and are inactivated by cytidine deaminase thus limiting their use in the treatment of BRCA [14].

Several studies investigated DNA methylation in relation to disease outcome and therapy resistance. Lin et al. observed significant differences in DNA methylation profiles between tamoxifen sensitive and tamoxifen resistant cell lines [15]. There, a large number of genes, several of which have been previously implicated in BRCA pathogenesis, were shown to have increased DNA methylation of their promoter CpG islands in the resistant cell lines. Similarly, Williams et al. observed a large number of hypermethylated genes in a tamoxifen-resistant cell line [13]. In a meta-analysis of two human BRCA gene expression datasets, 144 genes for which methylation levels had been linked to BRCA survival were shortlisted as putative epigenetic biomarkers of survival. Kaplan-Meier survival analysis on the expression of these genes further reduced this list to 48 genes, and a subsequent correlation analysis of gene expression and DNA methylation provided evidence for the potential association of DNA methylation with survival in different BRCA subtypes including ER+/HER2- [16]. Another study compared ductal carcinoma in situ to invasive BRCA and suggested that methylation changes indicate an early event in the progression of cancer and, therefore, might be of relevance for clinical decision making [17]. In contrast to studies that showed the impact of promoter methylation, it has also been demonstrated that endocrine response in cell lines is mainly modulated by methylation of estrogen-responsive enhancers [18]. There, increased *ESR1*-responsive enhancer methylation in primary tumours was found to be associated with endocrine resistance and disease relapse in ER+ (luminal A) human BRCA, suggesting that methylation levels can be used to identify patients that positively respond to ET. Note that, although limited ER-responsive enhancer methylation may already be present in the primary tumour, the analysis of methylation profiles of matched relapse samples showed that enhancer DNA methylation increased during treatment. Therefore, a combination of pre-existing and acquired differences in enhancer DNA methylation could be associated with the development of ET resistance.

Current evidence on the association of DNA methylation and endocrine resistance is largely based on cell line models. The largest BRCA patient cohort to study genome-wide DNA methylation profiles and their association with resistance to ET is provided by The Cancer Genome Atlas (TCGA) [19]. However, to the best of our knowledge, these data have hardly been used to find candidate methylation sites associated with endocrine resistance. One exception is a recent study by Zhang et al., who used the TCGA BRCA cohort to identify regions that were differentially methylated between patients resistant and sensitive to ET [20]. However, their analysis was based on only a small subset of 32 samples selected based on either short-term (less than 30 months; resistant) or long-term (more than 100 months; sensitive) survival.

In the current work we investigated if DNA methylation profiles of primary ER+/HER2- tumours provide information to predict endocrine resistance. We selected methylation profiles provided by TCGA from patients treated with tamoxifen or AIs, and assumed that patient survival is a proxy for absence of therapy resistance. To identify specific DNA methylation markers, we tested the association with survival using a Cox proportional hazards model. We were able to identify DNA methylation markers associated with patient outcome in a cohort of 552 ER+/HER2- patients, a sub-cohort of 172 patients treated with TAM, and a sub-cohort of 210 patients treated with AIs. We validated these markers and associated gene sets using DNA methylation profiles generated in a time course experiment using the T47D cell line treated with tamoxifen or deprived from estrogen.

## Methods

### Data

We used clinical, biospecimen, gene expression (RNAseq V2) and DNA methylation (Illumina Human Methylation 450 K) data of 1098 patients with breast invasive carcinoma from TCGA (cancergenome.nih.gov). Samples represented in TCGA were all collected prior to adjuvant therapy [21]. TCGA also recorded patient follow-up information describing clinical events such as type of treatment, the number of days from the date of initial pathological diagnosis to a new tumour event, death, and date of last contact. Since clinical and biospecimen data are scattered over multiple files in the TCGA repository, we first merged all information in a single table with one row per patient using the patient identifiers provided in the clinical and biospecimen data. Subsequently, we corrected drug names for tamoxifen and AIs (anastrozole, exemestane and letrozole) for spelling variants and mapped synonyms to their generic drug names (Additional File 1).

### Patient cohorts

**Fig. 1:**
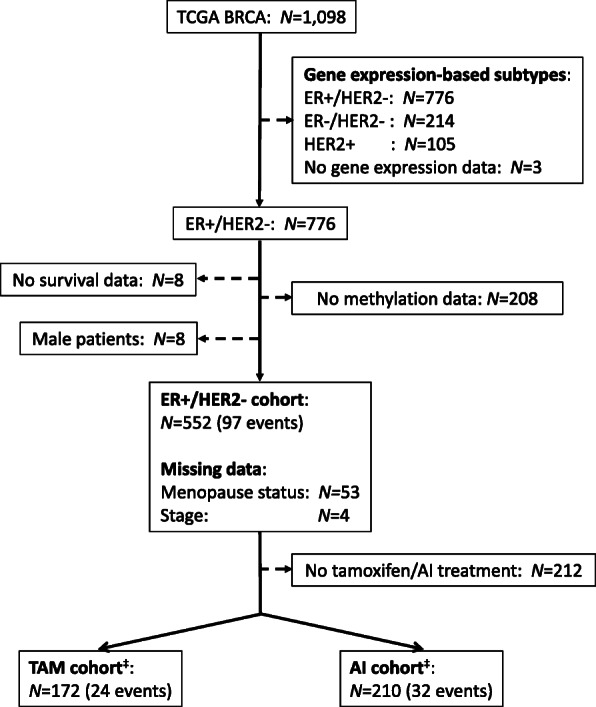
Study flow chart and cohort definition. This figure shows the steps taken to define each of the three cohorts. First the molecular subtype was determined using TCGA BRCA gene expression data and ER+/HER2- patient samples were selected. Next, patients without follow-up data and patients for whom no methylation profiles were measured were removed. Finally, male patients were removed leading to the study cohort of ER+/HER2- patients. Patients who received tamoxifen form the TAM sub-cohort and patients who received AI form the AI sub-cohort. Dashed arrows indicate filter steps. ‡42 patients received both tamoxifen and AI and are included in both the TAM and AI sub-cohort. No missing data for TAM and AI cohorts. AI, aromatase inhibitor; BRCA, breast invasive carcinoma; ER, estrogen receptor; HER2, human epidermal growth factor receptor 2; TAM, tamoxifen; TCGA, The Cancer Genome Atlas

For all patients with DNA methylation data available we selected data from primary tumours (indicated with “01” in the patient barcode) of female ER+/HER2- BRCA patients (Fig. 1). The molecular subtype was determined using TCGA gene expression data for these samples (see below). The ER+/HER2- cohort was further subdivided according to the endocrine treatment (AI or tamoxifen) that patients received during follow-up. Patients who received both drugs were included in both sub-cohorts. Consequently, we considered three patient cohorts, i.e., ER+/HER2-, AI, and TAM.

### Subtype determination

Information for BRCA subtyping by immunohistochemistry of ER or HER2 is missing for 192 out of 1098 patients. Therefore, we used TCGA BRCA RNAseq V2 gene expression data to determine molecular subtypes (Additional File 2). To this end, gene expression data from primary tumours were retrieved from the Genomic Data Commons legacy archive using the R package *TCGAbiolinks* [22]. RSEM estimated abundances were normalised using the upper quartile method from the R package *edgeR* [23] and subsequently log2-transformed with an offset of one. BRCA subtypes ER−/HER2-, HER2+, and the lowly proliferative ER+/HER2- (luminal A) and highly proliferative ER+/HER2- (luminal B) subtypes were determined using the SCMOD2 model from the R package *genefu* [24].

### DNA methylation data and pre-processing

Illumina Human Methylation 450 K raw data (IDAT files) for the patients in the cohorts defined above were retrieved from TCGA. Pre-processing was performed using the R package *minfi* [25]. Data were normalized using functional normalization with dye bias correction using a reference array [26]. Detection *p*-values were calculated for each methylation probe and 82,150 probes with an unreliable signal (*p* > 0.01) in one or more samples were removed. Probes corresponding to loci that contain a SNP in the CpG site or in the single-base extension site were removed. We also removed probes that have been shown to cross-hybridize to multiple genomic positions [27]. Finally, M-values were calculated and probes with low variation across samples (standard deviation of M-values ≤ 0.4) were removed. The final data set comprised 320,504 CpG loci. Probes were annotated to genes and enhancer regions using the R package *IlluminaHumanMethylation450kanno.ilmn12.hg19*.

### Survival analysis

#### Clinical variables

Based on literature [28–30] we selected menopause status (pre/post, after merging pre- and peri-menopausal; values ‘[Unknown]’ and ‘Indeterminate’ were considered missing), AI treatment (yes, no), tamoxifen treatment (yes, no), tumour stage (I-IV, after merging subcategories; stage X was considered missing), and age at diagnosis as candidate variables predictive of survival. We tested association with survival using the Cox proportional hazards model (R package *survival*). We defined an event as the first occurrence of a new tumour event or death. For patients without an event we used the latest contact date as provided by the clinical data (right censoring). To account for missing values for the variables menopause status and stage in the ER+/HER2- cohort we used multiple imputation (R package *mice*) to generate 50 datasets and perform survival analysis on each dataset separately [31]. The input data used for multiple imputation is available in Additional File 3. Rubin’s rule was applied to combine individual estimates and standard errors (SEs) of the model coefficients from each of the imputed datasets into an overall estimate and SE resulting in a single *p*-value for each variable. Clinical variables with a *p*-value < 0.10 in a univariable survival model were selected for inclusion in the multivariable survival model. Variables in the final multivariable model were determined using backward selection by iteratively removing variables with the highest *p*-value until all variables had a *p*-value < 0.05.

#### Single-locus survival analysis

Next we performed survival analysis to identify single methylation loci associated with patient survival using the methylation M-values in a Cox proportional hazards model. The models for each locus were adjusted for significant clinical variables from the multivariable model. To account for missing values for clinical variables, multiple imputation was used as described above. Resulting *p*-values were corrected for multiple testing using the Benjamini-Hochberg false discovery rate (FDR). Adjusted *p*-values < 0.05 were considered statistically significant. Subsequently, single-locus survival models were also adjusted for ER+/HER2- subtypes (luminal A/luminal B) in addition to the clinical variables selected above. Kaplan-Meier curves for individual loci were determined by calculating the median of the methylation levels over all patients in a cohort and then assigning a patient to a low (methylation level < median) or a high (methylation level ≥ median) group.

#### Multi-locus survival analysis

We used the Cox proportional hazards model with elastic net regularization (function cv.glmnet, R package *glmnet*) [32] to identify a signature of multiple methylation loci associated with survival. We followed a two-stage approach. First, the CpG signature was determined without including clinical variables using Cox regression with elastic net penalty. Secondly, from the resulting model the risk score (see below) was calculated and used in a new model that includes the clinical variables selected above in order to establish whether the methylation signature provided additional information compared to merely using clinical variables. Optimal values, minimizing the partial likelihood deviance, for the elastic net mixing parameter (α) and tuning parameter (λ) were determined by stratified (for event status) 10-fold cross-validation using a grid search varying α from 0 to 1 in steps of 0.1 and using 100 values for λ that were automatically generated for each α. We constructed one model for each of the three cohorts (ER+/HER2-, AI, TAM). Subsequently, for each cohort we used the identified signature to calculate a risk score for each patient:

$$\mathrm{risk}\ \mathrm{score}={\sum}_i{c}_i\ast {M}_i$$

where for CpG locus *i, c*_*i*_ denotes the corresponding coefficient in the Cox model and *M*_*i*_ the methylation M-value. Next, multivariable Cox proportional hazards regression was performed using the risk score as a variable and adjusting for significant clinical variables from the multivariable model. Missing values for the clinical variables were imputed as described above. Finally, the risk-score-based models were also adjusted for ER+/HER2- subtypes (luminal A/luminal B) in addition to the selected clinical variables. Kaplan-Meier curves were determined for two groups of patients by calculating the median of the risk scores over all patients in a cohort and then assigning a patient to a good (risk score < median) or a bad prognosis group (risk score ≥ median).

#### Stability of multi-locus signatures

To assess the stability of the multi-locus signatures 30 regularized Cox models were fitted using a stratified (for event status) selection of 90% of the samples for each cohort. We counted the number of times each CpG locus was included in the 30 signatures and then selected those CpGs that occurred in at least 6 or at least 21 signatures. We refer to the resulting signatures as stability signatures. Fisher’s exact test was used to determine the significance of the overlap between the original multi-locus signature and the stability signatures.

### Correlation between DNA methylation and gene expression

CpGs in single-locus and multi-locus signatures were annotated to their nearest gene(s) (package *IlluminaHumanMethylation450kanno.ilmn12.hg19*). For each signature Pearson correlation coefficients (and corresponding *p*-values) were calculated between the methylation and gene expression profiles of each CpG-gene pair. Resulting *p*-values were corrected for multiple testing in each signature using the Benjamini-Hochberg FDR.

### Methylation profiling of resistance acquisition in an ER+ breast cancer cell line

T47D cells were either treated with 100 nM 4-hydroxytamoxifen (TMX), long-term estrogen deprived (LTED; modelling AI treatment [33]) or not treated (wild type (WT)) in two biological replicates cultured for 7 and 5 months, respectively. DNA was extracted after 0, 1, 2, 5 and 7 (only one replicate) months. Methylation profiling was performed using the Illumina MethylationEPIC BeadChip platform at the Genomic and Proteomic Core Facility (DKFZ, Germany). For each sample two technical replicates were measured. Pre-processing was performed as described above, except that a single sample approach was used for dye bias correction. The 8682 probes with an unreliable signal (detection *p*-value > 0.01) in one or more samples were removed. Probes that cross-hybridized to multiple genomic positions as listed by Pidsley et al. [34] were removed. No filtering based on M-values was performed. The final data set contains 786,872 CpG loci. Using the resulting M-values CpG-wise linear models were fitted with coefficients for each treatment (TMX, LTED, WT) and time point combination. In addition, we included a coefficient to correct for systematic differences between the two biological replicates (R package *limma*). For both LTED and TMX treated cells, contrasts were made between each individual time point *t* and the WT cell line at baseline, that is, LTED_*t*_ – WT_0_ and TMX_*t*_ – WT_0_, respectively. The comparison of the average of TMX and LTED treated cells versus WT baseline was estimated via the contrast (LTED_*t*_ + TMX_*t*_)/2 – WT_0_. Differential methylation was assessed using empirical Bayes moderated statistics while also including the consensus correlation within pairs of technical replicates in the linear model fit (function duplicateCorrelation, *limma* package). The resulting signatures are referred to as the LTED, TMX and TMX/LTED signatures.

### Enrichment analysis

We performed generalized gene set testing with a hypergeometric test using the gsameth function (R package *missMethyl*) to test if significant CpG sites are enriched in selected pathways [35]. For the single-locus survival analysis, signatures were defined as those CpGs with *p*-value < 0.006 (TAM, AI) and *p*-value < 0.002 (ER+/HER2-) corresponding to signatures of ~ 2500 CpGs. For the T47D RA experiment signatures were defined as the top 10,000 CpGs ranked on *p*-value as determined using a moderated F-test (*limma* package), which tests whether a CpG is differentially methylated at any time point versus WT, for the three sets of contrasts (TMX, LTED, TMX/LTED) described above. We used a combination of Hallmark gene sets (collection H) and a subset of 16 curated gene sets (collection C2; gene set name contained either “tamoxifen” or “endocrine_therapy”) from the Molecular Signatures Database (MSigDB) v7.0 (Entrez Gene ID version) [36]. Resulting *p*-values were corrected for multiple testing using the Benjamini-Hochberg FDR.

**Fig. 2:**
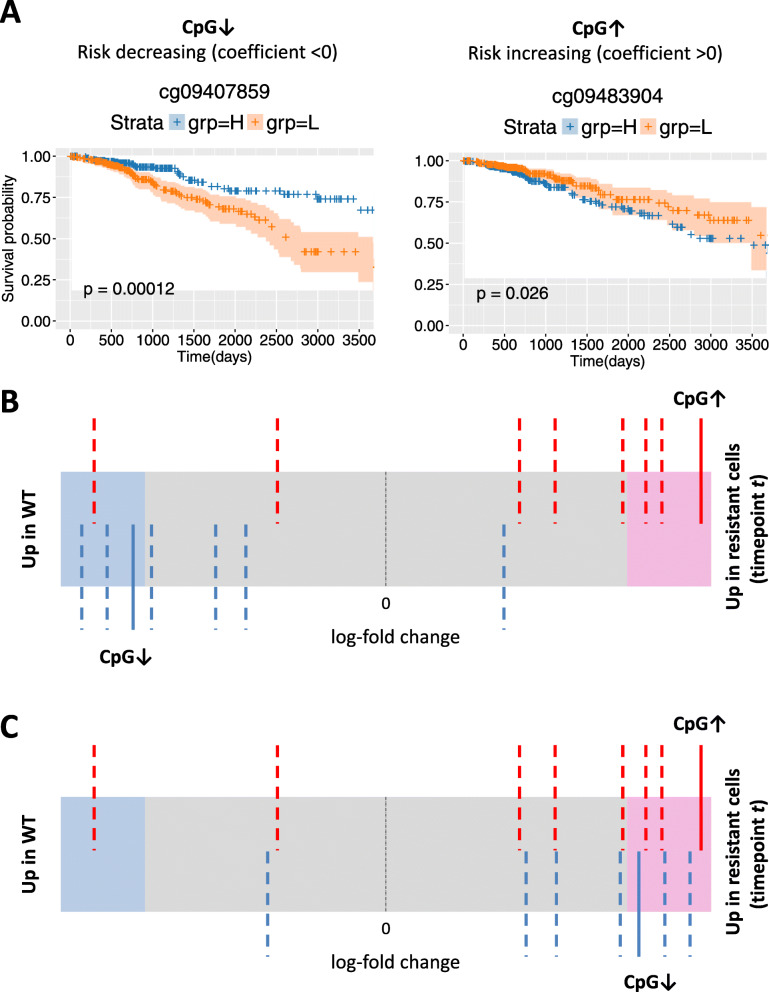
Validation of survival signatures in T47D resistance acquisition experiment. **a** Kaplan-Meier plots for two selected CpGs significantly associated with survival in the ER+/HER2- cohort. Patients were stratified based on the methylation levels of a risk decreasing locus CpG↓ (left; higher methylation is associated with longer survival) and a risk increasing locus CpG↑ (right; higher methylation is associated with shorter survival). H, methylation levels above median; L, methylation levels below median. Shaded areas in the Kaplan-Meier plot denote the 95% CI in the H and L strata. *P*-values are based on a log-rank test. **b** Example of a barcode enrichment plot for a TCGA BRCA survival signature in the cell line comparison of treated (LTED or TMX) samples at time point *t* versus WT baseline. All methylation loci are ranked from left to right by increasing log-fold change in the cell line comparison under consideration and represented by a shaded bar. Loci within the survival signature are represented by vertical bars. Red bars correspond to risk increasing loci (for example, CpG↑ indicated with a solid bar), blue bars correspond to risk decreasing loci (for example, CpG↓ indicated with a solid bar). In this example, the risk increasing loci tend to be hypermethylated (log-fold change > 0) in the treated cell line and the risk decreasing loci tend to be hypomethylated (log-fold change < 0). That is, most loci change in the same direction in the survival signature and the T47D RA experiment. **c** When using directional weights of 1 and − 1 for risk increasing and risk decreasing loci respectively, the blue bars are mirrored across the black dashed line at a log-fold-change of 0. In this case for a ROAST gene set test, the alternative hypothesis ‘up’ corresponds to methylation levels changing in the same direction whereas the alternative hypothesis ‘down’ corresponds to a change in the opposite direction

We also tested whether the methylation loci identified from the TCGA BRCA single-locus and multi-locus signatures (based on Illumina 450 K arrays) and represented on the Illumina EPIC array were enriched in the T47D RA experiment using ROAST rotation-based gene set tests (*limma* package) [37]. Enrichment of TAM and AI survival signatures was assessed using the comparisons of respectively TMX and LTED treated cells to WT baseline described above. Enrichment of the ER+/HER2- survival signature was assessed using the comparison of the average of TMX and LTED treated cells versus WT baseline described above. ROAST *p*-values were calculated_,_ for two alternative hypotheses denoted as ‘up’ and ‘down’ using 9999 rotations. In the ROAST analyses directional contribution weights of 1 or − 1 were used depending on whether a CpG of the signature under consideration had a positive (corresponding to increased risk of an event) or negative (corresponding to decreased risk of an event) coefficient in the corresponding Cox model. In this case, the alternative hypothesis ‘up’ corresponds to methylation levels changing in the same direction in the TCGA BRCA survival signature and in the T47D RA experiment, whereas the alternative hypothesis ‘down’ corresponds to a change in the opposite direction (Fig. 2). The two-sided directional *p*-value is reported.

### Quantitative real-time PCR

Total RNA was isolated from WT and T47D cells treated with tamoxifen or deprived from estrogen with RNeasy Mini kit (Qiagen, Hilden, Germany) according to the manufacturer’s instructions and treated with DNase Max Kit (Qiagen). cDNA was synthesized with the Revert Aid H Minus First Strand cDNA Synthesis Kit (Fermentas, Waltham, MA, USA). Quantitative real-time PCR (qRT-PCR) reactions for target genes were performed with the Applied Biosystems QuantStudio™ 3 Real-Time PCR System, using probes from the Universal Probe Library, UPL (Roche Diagnostics, Mannheim, Germany). The data were analyzed using the SDS software with the ΔΔCt method. The Ct values were normalized to the housekeeping gene *ACTB*.

## Results

### Clinical variables are associated with survival in ER+/HER2- cohort

For the TCGA BRCA ER+/HER2- cohort (*N* = 552, Fig. 1) we assessed whether the clinical variables menopause status, AI treatment, tamoxifen treatment, tumour stage and age at diagnosis were associated with survival, with an event defined as first occurrence of a new tumour event or death. In a univariable Cox proportional hazards model tumour stage (HR 1.92, 95% CI 1.43–2.59; *p* = 1.63E-05) and age at diagnosis (HR 1.03, 95% CI 1.01–1.05; *p* = 2.40E-04) are significantly associated with survival (Table 1). This is in agreement with previous findings that a more advanced tumour stage and increased age are associated with poorer outcome [38]. Tamoxifen treatment, AI treatment and menopause status are not significantly associated with survival in our cohort. When we included the clinical variables in a multivariable Cox proportional hazards model, tumour stage, age and AI treatment were selected for inclusion in the final multivariable model using backward selection (Table 2).

**Table 1:** Univariable Cox proportional hazards model

	HR	95% CI	*P*-value
Stage (per stage increment)	1.92	1.43–2.59	1.63E-05
Age (per 1-yr increment)	1.03	1.01–1.05	2.40E-04
AI treatment (vs. no AI treatment)	0.68	0.45–1.05	0.0812
Post-menopausal (vs. pre-menopausal)	1.52	0.94–2.45	0.0913
Tamoxifen treatment (vs. no tamoxifen treatment)	0.67	0.42–1.07	0.0921

Univariable Cox proportional hazards model for clinical variables (ER+/HER2- cohort). *HR* hazard ratio, *CI* confidence interval, *AI* aromatase inhibitor

**Table 2:** Multivariable Cox proportional hazards model

	HR	95% CI	*P*-value
Stage (per stage increment)	2.15	1.61–2.89	3.05E-07
Age (per 1-yr increment)	1.04	1.02–1.05	2.48E-06
AI treatment (vs. no AI treatment)	0.61	0.40–0.94	0.026

Multivariable Cox proportional hazards model for clinical variables (ER+/HER2- cohort). *HR* hazard ratio, *CI* confidence interval, *AI* aromatase inhibitor

### Single methylation loci associated with survival

To identify individual methylation loci associated with survival we fitted 320,504 Cox proportional hazard models using the M-value of each CpG while adjusting for the clinical variables selected in the multivariable model above (tumour stage, age and AI treatment (ER+/HER2- cohort only)). This resulted in 134, 5 and 1 CpGs for which DNA methylation is significantly (adjusted *p*-value < 0.05) associated with survival in the ER+/HER2-, TAM, and AI cohort respectively (Additional File 4). The Kaplan-Meier curves show a significant difference in survival between the two groups stratified on median methylation level for nearly all selected loci (Additional File 5). Interestingly, apart from three CpGs in the ER+/HER2- signature, for all of the CpGs increased methylation is associated with decreased risk of an event. Additional File 6 shows the overlap of the signatures for the three cohorts. Three out of five methylation loci from the TAM signature are also found in the ER+/HER2- signature and, consequently, the other two loci are specific for tamoxifen treated patients. Since all patients in the TAM cohort are also included in the ER+/HER2- cohort, overlap between the signatures is expected. TAM and AI signatures do not share methylation loci. ER+/HER2- and TAM signatures are enriched for enhancer CpGs (ER+/HER2-: 36%, *p* = 0.0005; TAM: 80%, *p* = 0.0113; Fisher’s exact test, one-sided).

For all selected patients we had paired DNA methylation and gene expression data (Fig. 1). We therefore calculated the Pearson correlation coefficient between the methylation profile of each locus in any of the three signatures and gene expression of the gene(s) closest to that locus (Additional File 4). DNA methylation is significantly (adjusted *p*-value < 0.05) (anti-)correlated with gene expression for 52 (of 136), 3 (of 5) and 2 (of 2) CpG-gene pairs in the ER+/HER2-, TAM and AI signature respectively.

To gain insight in the main biological processes involved in differences in survival, we performed gene set enrichment analyses on genes linked to CpG loci associated with survival. All three signatures are significantly enriched (FDR < 0.1) in gene sets associated with ET or endocrine resistance, genes activated when upregulating the PI3K/AKT/mTOR pathway and genes upregulated in response to TGFB1, which have both been implicated in endocrine resistance [39, 40] (Additional File 12).

### Multi-locus methylation signature associated with survival

**Fig. 3:**
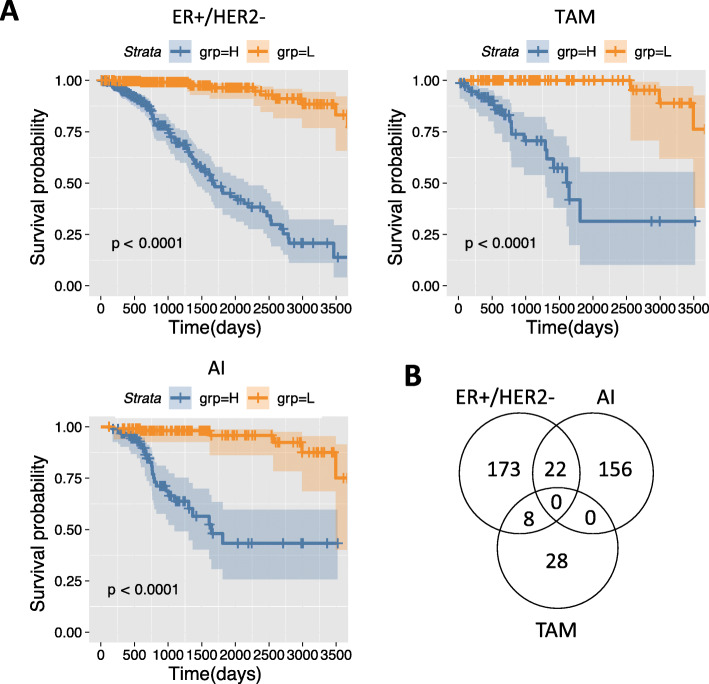
Multi-locus survival analysis. **a** Kaplan-Meier plots of the patients stratified based on the risk scores of the multi-locus signature in ER+/HER2, TAM and AI cohorts. H, risk score above median; L, risk score below median. Shaded areas denote the 95% CI in the H and L strata. *P*-values are based on a log-rank test. **b** Venn diagram denoting the number of methylation loci in the multi-locus signatures for the ER+/HER2-, TAM, and AI cohorts’

Next we performed a multivariable analysis with elastic net penalty to find combinations of methylation loci associated with survival in a Cox proportional hazards model. This resulted in 203, 36 and 178 CpGs that are included in the survival signatures of the ER+/HER2-, TAM, and AI cohort respectively (Additional File 7). The ER+/HER2- and AI signatures are enriched for enhancer loci (ER+/HER2-: 36%, *p* = 1.79E-05; AI: 29%, *p* = 0.044; Fisher’s exact test, one-sided), whereas the TAM signature is not significantly enriched for enhancer loci (TAM: 36%, *p* = 0.051; Fisher’s exact test, one-sided). The risk score calculated from the multi-locus signature and adjusted for tumour stage, age and AI treatment (ER+/HER2- cohort only) is significantly associated with survival (*p* < 10E-12) for all three cohorts (Additional File 8) indicating that DNA methylation is an independent factor in predicting survival. The risk scores calculated from the multi-locus signatures stratify the patients in two groups for each cohort (Fig. 3a).

There is no overlap between the signatures of TAM and AI cohorts. However, the ER+/HER2- signature partly overlaps with the TAM and AI signatures (Fig. 3b). The coefficients in the Cox models corresponding to the overlapping loci have an identical sign in both cohorts. The multi-locus signatures include a large number of methylation loci that were also identified in the corresponding single-locus survival analysis. Out of 203 methylation loci in the ER+/HER2- multi-locus signature 34 were also found in the single-locus signature (Additional File 9). Moreover, all methylation loci in the TAM and AI single-locus signatures, five and one respectively, are part of the corresponding multi-locus signature.

We assessed the stability of the multi-locus signatures using a 10% leave-out test. The stability signature is enriched in the original multi-locus signature for each corresponding cohort (*p* < 0.05; Additional File 10).

We calculated the Pearson correlation coefficient between the methylation profile of each locus in any of the three multi-locus signatures and gene expression of the gene(s) closest to that locus (Additional File 7). DNA methylation is significantly (adjusted *p*-value < 0.05) (anti-)correlated with gene expression for 109 (of 235), 17 (of 37) and 57 (of 181) CpG-gene pairs in the ER+/HER2-, TAM and AI signature respectively.

### Profiling of resistance development in T47D cells

To investigate the possible association between DNA methylation of the loci identified in the survival analyses and ET resistance in more detail, we performed a time course experiment using the T47D ER+ BRCA cell line treated with tamoxifen or long-term estrogen deprived. qRT-PCR analysis showed that endocrine resistance associated genes *HDAC9* [41] and *CD36* [42] are indeed significantly increased in the treated cells compared to WT (Additional File 11). Also known tamoxifen induced genes *KRT4* and *FGF12* [43] show a significant upregulation in the treated cells. Next, we generated genome-wide DNA methylation profiles for both treatments on five different time points (0 (=WT), 1, 2, 5, and 7 months). We identified three signatures, corresponding to CpGs that were differentially methylated over time in TMX treated cells, LTED cells, and in the comparison of the average of TMX and LTED cells versus WT. These signatures consist of thousands of loci that are significantly differentially methylated over time versus WT. To gain insight in the main biological processes involved in RA, we performed gene set enrichment analyses on genes associated with differentially methylated loci. All three signatures are significantly enriched (FDR < 0.1) in gene sets associated with ET or endocrine resistance, gene sets related to metastasis such as the epithelial-mesenchymal transition, gene sets corresponding to signaling pathways implicated in endocrine resistance such as hedgehog signaling [44], and a gene set defining early response to estrogen (Additional File 12).

### Validation of survival signatures in T47D resistance acquisition experiment

We then investigated the concordance between the CpG loci in the survival signatures and in the RA signatures (Fig. 2). The multi-locus survival signatures for ER+/HER2- and AI are significantly enriched in the comparison of the last time point (7 months) versus WT baseline in the T47D RA experiment (ER+/HER2-: *p* = 0.0017, AI: *p* = 0.0222; direction: ‘up’; Table 3). The signatures are not enriched at earlier time points. However, the proportion of CpGs contributing to enrichment in the same direction (‘up’) increases over time until it becomes significant for the last time point. The single-locus survival signature for TAM is also significantly enriched at the 7-month time point in the T47D RA experiment (*p* = 0.0032), but not for ER+/HER2- despite an increasing trend in the proportion of CpGs contributing to enrichment in the same direction (‘up’) over time (Table 4). The single-locus AI signature consists of only one CpG and an enrichment analysis is therefore not possible. However, for this locus the change in methylation level when comparing LTED treated cells with WT baseline is not concordant with the log-hazard ratio for that locus (data not shown).

**Table 3:** ROAST test results for the multi-locus signatures

Time point	Direction	*P*-value	Prop. (down)	Prop. (up)
**ER+/HER2- (193 CpG sites)**				
1	Up	0.3948	0.06	0.07
2	Up	0.6744	0.24	0.24
5	Up	0.3137	0.26	0.28
7	Up	**0.0017**	0.23	0.35
**TAM (32 CpG sites)**				
1	Down	0.0995	0.06	0.03
2	Down	0.1736	0.16	0.06
5	Down	0.0037	0.28	0.06
7	Down	0.0028	0.22	0.13
**AI (164 CpG sites)**				
1	Up	0.1004	0.05	0.13
2	Up	0.5434	0.15	0.22
5	Down	0.2088	0.24	0.23
7	Up	**0.0222**	0.15	0.26

Direction indicates the direction of change. Methylation loci were weighted by their direction of change in the survival signature. ‘Up’ therefore corresponds to changes in the same direction in the survival signature and in the T47D RA experiment. That is, if a locus is risk in/decreasing in the survival signature than it is hyper/hypomethylated in the cell line signature for the indicated time point as compared to WT baseline. ‘Down’ corresponds to changes in the opposite direction. Prop., proportion of loci in the signature contributing to the estimated *p*-value and direction. Significant *p*-values (< 0.05) for concordant changes (‘Up’) are indicated in bold

**Table 4:** ROAST test results for the single-locus signatures

Time point	ER+/HER2- (128 CpG sites)				TAM (5 CpG sites)			
	Direction	*P*-value	Prop. (down)	Prop. (up)	Direction	*P*-value	Prop. (down)	Prop. (up)
1	Down	0.6920	0.11	0.07	Down	0.7699	0	0
2	Up	0.7571	0.16	0.20	Up	0.8784	0	0
5	Down	0.0365	0.30	0.25	Up	0.1013	0.2	0.4
7	Up	0.3455	0.25	0.31	Up	**0.0032**	0	0.6

Direction indicates the direction of change. Methylation loci were weighted by their direction of change in the survival signature. ‘Up’ therefore corresponds to changes in the same direction in the survival signature and in the T47D RA experiment. That is, if a locus is risk in/decreasing in the survival signature than it is hyper/hypomethylated in the cell line signature for the indicated time point as compared to WT baseline. ‘Down’ corresponds to changes in the opposite direction. Prop., proportion of loci in the signature contributing to the estimated p-value and direction. Significant p-values (< 0.05) for concordant changes (‘Up’) are indicated in bold

**Fig. 4:**
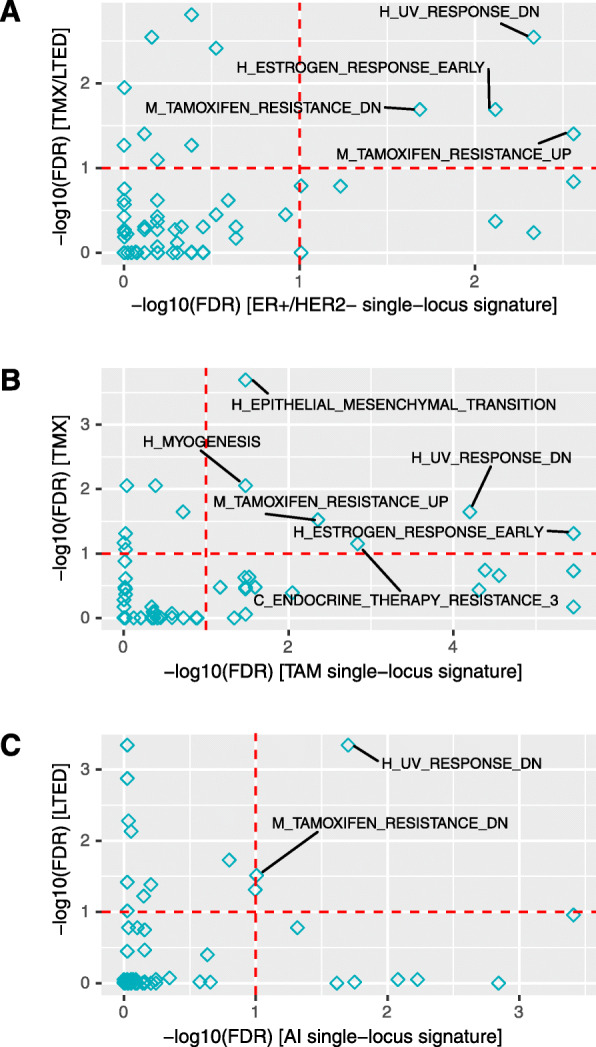
Gene sets enriched in single-locus survival and resistance acquisition signatures. Gene set enrichment analysis of single-locus survival (*x*-axis) and RA signatures (*y*-axis). **a** T47D TMX/LTED signature versus ER+/HER2− single-locus signature. **b** T47D TMX signature versus TAM single-locus signature. **c** T47D LTED signature versus AI single-locus signature. Each diamond represents either a Hallmark (H) gene set or a curated gene set (C: Creighton, M: Massarweh) related to tamoxifen treatment or ET from the Molecular Signatures Database. Gene sets significantly enriched (FDR < 0.1, that is -log10(FDR) > 1, indicated by the red dashed lines) in both signatures are labelled with their name. See Additional File 12 for a version of this figure in which more gene sets are labelled

Also in terms of gene sets there is overlap between the sets enriched in both the single-locus survival signatures and RA signatures. Interestingly, one of the most significant gene sets in all six signatures consists of genes down-regulated in response to ultraviolet (UV) radiation. Many genes in this gene set are related to cell motility. Indeed, upon UV stress cells down regulate non-essential processes such as invasion and motility, whereas these processes are upregulated in resistant cells that become more invasive. Four gene sets are significantly enriched (FDR < 0.1) in the two ER+/HER2- signatures.. These include sets associated with endocrine resistance and a gene set defining early response to estrogen (Fig. 4a, Additional File 12A). Six gene sets, several of them related to endocrine resistance, are significantly enriched in the two tamoxifen signatures (Fig. 4b, Additional File 12B). Two gene sets are significantly enriched in both the AI and LTED signature (Fig. 4c, Additional File 12C).

## Discussion

We investigated whether TCGA DNA methylation profiles measured in primary ER+/HER2- tumours can be used to predict development of resistance to ET in two sub-cohorts of patients treated with tamoxifen or AI. Using a single-locus Cox proportional hazard model we were able to identify 134, 5 and 1 CpGs for which DNA methylation is significantly associated with survival in the ER+/HER2-, TAM and AI cohorts respectively, while the corresponding multi-locus signatures consisted of 203, 36 and 178 CpGs. The multi-locus signatures showed a large overlap of 25, 100, and 100% with the ER+/HER2-, TAM and AI single-locus signatures respectively. The risk scores of the multi-locus signatures were significantly associated with survival. Moreover, we found that the ER+/HER2- and TAM single-locus and ER+/HER2- and AI multi-locus signatures were significantly enriched for CpGs in enhancer regions suggesting a functional effect (on gene expression) [18]. For both the single-locus signatures (Additional File 6) and the multi-locus signatures (Fig. 3b) we observed no overlap of loci associated with survival between the AI and TAM cohorts. This could be indicative of a difference in development of resistance against tamoxifen or AI. This is in line with earlier observations in endocrine-resistant cells compared with wild type MCF7 cells, which also showed limited overlap in their response to tamoxifen and estrogen deprivation in terms of their gene expression [10] and DNA methylation profiles [18].

In our analyses we adjusted for clinical variables associated with survival (tumour stage, age and AI treatment (ER+/HER2- cohort only)) in order to estimate the independent effect of methylation on survival. It has been shown that methylation profiles can discriminate between the ER+/HER2- subtypes luminal A and B [45]. Moreover, patients with a luminal B tumour have worse prognosis compared to patients with a luminal A tumour [46], which is also the case in our ER+/HER2- cohort (HR 2.04, 95%CI 1.11–3.74, *p* = 0.020). We, therefore, also performed survival analyses adjusted for luminal status in addition to the clinical variables mentioned earlier. The single-locus signatures with correction for luminal status showed a considerable overlap of 85, 40, and 100% with the original (that is, without correction for luminal status) ER+/HER2-, TAM and AI single-locus signatures respectively (Additional File 13). Notably, all CpGs included in the original single-locus signatures still have an FDR < 0.15 after correction for luminal status. The risk scores of the original multi-locus signatures were also significantly associated with survival after correction for luminal status (Additional File 13). In summary, the methylation signatures we identified are associated with survival independently of luminal status.

We note that although the methylation profiles provided by TCGA are measured in untreated primary tumour samples, treatment regimens after initial diagnosis are heterogeneous. Some patients received adjuvant chemotherapy and/or radiotherapy next to ET and 42 patients in the TAM and AI cohorts received both types of endocrine treatments. Moreover, the duration of (endocrine) treatment varied among patients. Furthermore, treatment information may not be complete [21]. These aspects were not taken into account in our analyses and might have biased the results. We also acknowledge that this study is limited by the relatively modest number of events (i.e., new tumour event, death) for the different cohorts (ER+/HER2-: 97 events in 552 patients; TAM: 24 events in 172 patients; AI: 32 events in 210 patients) due to the relatively short follow-up time. This affects statistical power to identify methylation loci associated with survival.

In this study we assumed that the methylation events in the primary tumour, rather than acquired methylation during tumour progression, are associated with patient survival as a proxy for development of therapy resistance. To validate our results we aimed to use methylation profiles from the International Cancer Genome Consortium (ICGC). However, the number of patients in the ICGC BRCA cohort with reliable information on endocrine treatment was too small to make such a comparison meaningful. Instead, we used DNA methylation measurements obtained from T47D cells as a model system for RA in ER+ luminal A BRCA. We showed that our multi-locus signatures for the ER+/HER2- and AI cohorts were conserved among the loci that are differentially methylated in endocrine-resistant T47D cells. Similarly, our single-locus signature for the TAM cohort was also significantly enriched in the T47D experiment. At the gene set level, several sets related to ET and endocrine resistance were significantly enriched in both the survival and RA signatures. Although this is not a final validation of our results, it strongly suggests that the loci we identified from primary tumours, that is prior to any endocrine treatment, are also associated with endocrine resistance.

**Fig. 5:**
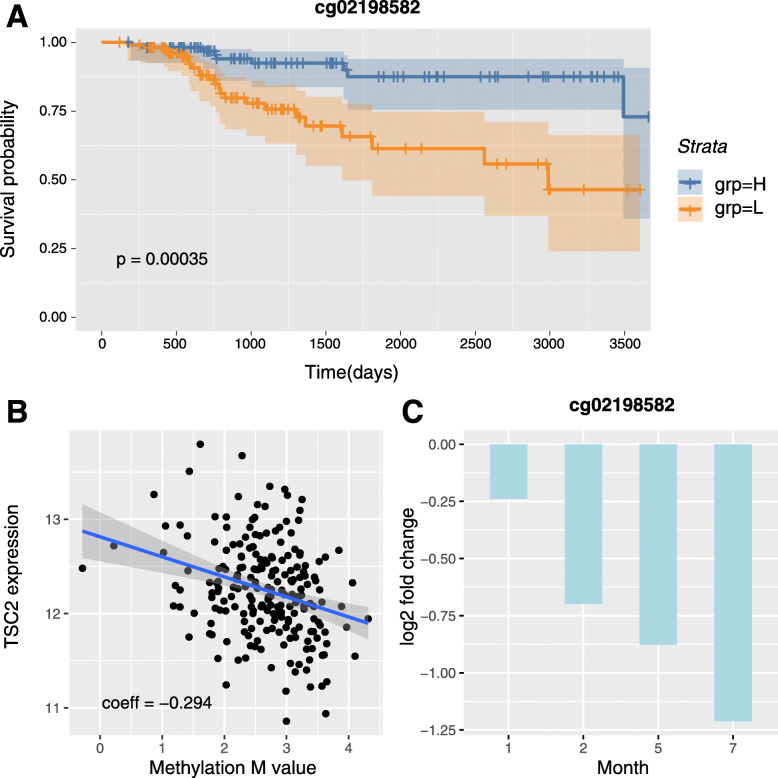
Association of methylation levels of CpG site cg02198582 with survival and resistance acquisition and its correlation with *TSC2* expression levels. **a** Kaplan-Meier plot for CpG site cg02198582 located in the gene body of *TSC2* and significantly associated with survival in the AI cohort. Patients were stratified based on methylation levels. H, methylation levels above median; L, methylation levels below median. Shaded areas in the Kaplan-Meier plot denote the 95% CI in the H and L strata. P-value is based on a log-rank test. **b** Correlation between paired DNA methylation and gene expression profiles (cg02198582, *TSC2*). Each circle corresponds to a patient sample in the AI cohort. The Pearson correlation coefficient is indicated, together with the corresponding regression line and its 95% CI. **c** Log2-fold change of the methylation M-values of cg02198582 inT47D LTED versus WT cells

CpGs with concordant significant changes in the survival and RA signatures and with significant (anti-)correlation between paired DNA methylation and gene expression profiles in TCGA BRCA are promising candidates for further investigation and are listed in Additional File 14. Most genes associated with these CpG sites have been implicated in survival and resistance related processes in BRCA. In particular, high levels of *TSC2* and *PXN* are associated with decreased metastasis-free survival [47, 48]. This is in agreement with our findings that lower methylation of the corresponding CpG loci is associated with decreased survival and that their DNA methylation profile is negatively correlated with gene expression (Fig. 5a-b, Additional File 15A-B). Interestingly, in the T47D RA experiment these loci are also significantly hypomethylated in resistant cells compared with WT (Fig. 5c, Additional File 15C). In the ER+/HER2- single-locus signature, the cg07145834 locus in the 5’UTR of *ZHX2* was selected. Low levels of *ZHX2* are associated with better overall survival [49], in agreement with the findings from this study that higher methylation of the corresponding CpG locus is associated with increased survival, its DNA methylation profile is positively correlated with *ZHX2* gene expression, and the CpG locus is hypomethylated in resistant cells compared with WT T47D cells (Additional File 15D-F).

Stone et al. [18] recently demonstrated in a small cohort of patients who received endocrine treatment for at least five years that methylation levels in selected ESR1-enhancer loci were significantly increased in primary tumours of patients who relapsed within six years as compared to patients with 14-year relapse free survival. Moreover, these differences were even more pronounced in matched local relapse samples. DNA methylation data measured in a large number of pre- and post-treatment samples obtained from patients who received ET that either relapsed due to endocrine resistance or remained relapse-free will enable validation of the signatures identified in this and other studies. Moreover, such a cohort enables comparison of methylation levels in paired primary and local relapse samples providing the opportunity to identify epigenetic drivers of endocrine resistance [50].

## Conclusions

In this study we identified individual and multivariable DNA methylation markers associated with survival and resistance in a large cohort of 552 ER+/HER2- BRCA patients from The Cancer Genome Atlas. Survival signatures were validated using time series DNA methylation profiles of T47D cells during development of resistance to endocrine therapy. A number of promising targets with concordant significant changes in survival and RA signatures were identified. These include CpG sites associated with *TSC2, PXN* and *ZHX2* that have all been implicated in survival related processes in BRCA. Our results suggest that methylation signatures associated with the development of endocrine resistance can also be identified in primary breast tumours prior to any endocrine treatment.

## Supplementary information

**Additional file 1.** Mapping to generic drug names. Overview of synonyms and spelling variants for drug names used in TCGA BRCA and their mapping to a generic drug name used in our study.

**Additional file 2.** Molecular subtypes. Overview of the molecular subtype frequency as determined by immunohistochemistry of ER and HER2 and as predicted by the SCMOD2 model (R package *genefu*) using TGCA BRCA primary tumour gene expression data. Subtypes are listed for the 1095 patients for whom gene expression data is available (Fig. 1).

**Additional file 3.** Sample annotation. Sample annotation for the 552 patients in the ER+/HER2- cohort. The first sheet provides a short definition of the variables included in the second sheet.

**Additional file 4.** Single-locus survival analysis. Results of single-locus survival analysis on ER+/HER2-, TAM and AI cohorts. For each CpG the results of the correlation analysis and of the differential methylation analysis of month 7 versus WT in the T47D RA experiment are also included.

**Additional file 5 **Single-locus Kaplan-Meier plots. Kaplan-Meier plots for each CpG site from the single-locus signatures. Patients were stratified based on the methylation levels of the indicated locus in ER+/HER2, TAM and AI cohorts. H, methylation level above median; L, methylation level below median. Shaded areas denote the 95% CI in the H and L strata. *P*-values are based on a log-rank test.

**Additional file 6.** Single-locus Venn diagram. Venn diagram of the single-locus signatures in the ER+/HER2-, TAM and AI cohorts.

**Additional file 7.** Multi-locus survival analysis. Results of multi-locus survival analysis on ER+/HER2-, TAM and AI cohorts. For each CpG the results of the correlation analysis and of the differential methylation analysis of month 7 versus WT in the T47D RA experiment are also included.

**Additional file 8.** Survival analysis using risk score. Results of survival analysis of the multi-locus signature using the risk score corrected for selected clinical variables in ER+/HER2-, TAM and AI cohorts.

**Additional file 9.** Overlap between single-locus and multi-locus signatures. Venn diagrams of the overlap between single-locus and multi-locus signatures in the three cohorts ER+/HER2-, TAM and AI. (PPTX 41 kb)

**Additional file 10.** Stability of multi-locus signatures. Results of Fisher’s exact test to determine the significance of the overlap between the original multi-locus signature and the stability signature.

**Additional file 11.** qRT-PCR results. Primer sequences and gene expression levels of CD36, FGF12, HDAC9, and KRT4 determined by qRT-PCR after treatment with tamoxifen or long-term estrogen deprivation relative to their expression in untreated T47D cells.

**Additional file 12.** Gene set enrichment analysis. Gene set enrichment analysis of single-locus survival (*x*-axis) and RA signatures (*y*-axis). (A) T47D TMX/LTED signature versus ER+/HER2− single-locus signature. (B) T47D TMX signatuare versus TAM single-locus signature. (C) T47D LTED signature versus AI single-locus signature. Each diamond represents either a Hallmark gene set or a curated gene set related to tamoxifen treatment or ET from the Molecular Signatures Database. Gene sets significantly enriched (FDR < 0.1, that is -log10(FDR) > 1, indicated by the red dashed lines) in at least one of the two signatures are labelled with their name. Purple: gene sets that are significantly enriched in all three survival signatures. Red: gene sets that are significantly enriched in all three RA signatures. Blue: gene sets that are significantly enriched in all six signatures.

**Additional file 13. **Survival analyses including luminal status. Reanalysis when also including luminal status in the (*i*) multivariable survival analysis, (*ii*) single-locus survival analysis, and (iii) risk score for the multi-locus signature.

**Additional file 14. **CpGs with concordant significant changes in the survival and resistance acquisition signatures and with significant correlation between paired DNA methylation and gene expression profiles. CpGs in single-locus (Additional File 4) and multi-locus (Additional File 7) survival signatures were selected according to three additional criteria: (*i*) CpG DNA methylation is significantly (adjusted *p*-value < 0.05) (anti-)correlated with expression of the nearby gene(s), (*ii*) CpG is also significantly differentially methylated (adjusted p-value < 0.05) in the corresponding RA signature at month 7 versus WT, (*iii*) CpG changes concordantly in survival and corresponding RA signature, that is, risk increasing loci are hypermethylated and risk decreasing loci are hypomethylated.

**Additional file 15. **Association of methylation levels of selected CpG sites with survival and resistance acquisition and their correlation with expression levels of the associated genes. (A,D) Kaplan-Meier plot for CpG site cg14094027 located in the gene body of *PXN* (A) and CpG site cg07145834 located in the 5’UTR of *ZHX2* (D), both significantly associated with survival in the ER+/HER2- cohort. Patients were stratified based on methylation levels. H, methylation levels above median; L, methylation levels below median. Shaded areas in the Kaplan-Meier plot denote the 95% CI in the H and L strata. *P*-values are based on a log-rank test. (B,E) Correlation between paired DNA methylation and gene expression profiles (B: cg14094027, PXN; E: cg07145834, *ZHX2*). Each circle corresponds to a patient sample in the ER+/HER2- cohort. The Pearson correlation coefficient is indicated, together with the corresponding regression line and its 95% CI. (C,F) Log2-fold change of the methylation M-values of cg14094027 (C) and cg07145834 (F) in the comparison of T47D TMX/LTED versus WT.

## Data Availability

The DNA methylation dataset supporting the conclusions of this article is available in the Genomics Data Commons Legacy Archive (https://portal.gdc.cancer.gov/legacy-archive) repository. The other datasets supporting the conclusions of this article are included within the article and its additional files.

## Abbreviations

AI: Aromatase inhibitor
BRCA: Breast cancer
CI: Confidence interval
ESR1: Estrogen receptor 1
ER: Estrogen receptor
ET: Endocrine therapy
FDR: False discovery rate
H3K27ac: Acetylation of lysine 27 on histone 3
HR: Hazard ratio
LTED: Long-term estrogen deprived/deprivation
RA: Resistance acquisition
SE: Standard error
TAM: Tamoxifen (patient data)
TCGA: The Cancer Genome Atlas
TMX: Tamoxifen (cell line experiment)
