# Supplementary material for: Candidate methylation sites associated with endocrine therapy resistance in ER+/HER2- breast cancer
Source: BMC Cancer. 2020 Jul 19;20:676. doi: 10.1186/s12885-020-07100-z (PMC7368985; doi:10.1186/s12885-020-07100-z)
Supplement: Supplementary file 5 — Additional file 5 Single-locus Kaplan-Meier plots. Kaplan-Meier plots for each CpG site from the single-locus signatures. Patients were stratified based on the methylation levels of the indicated locus in ER+/HER2, TAM and AI cohorts. H, methylation level above median; L, methylation level below median. Shaded areas denote the 95% CI in the H and L strata. P-values are based on a log-rank test. [file 12885_2020_7100_MOESM5_ESM.pdf]

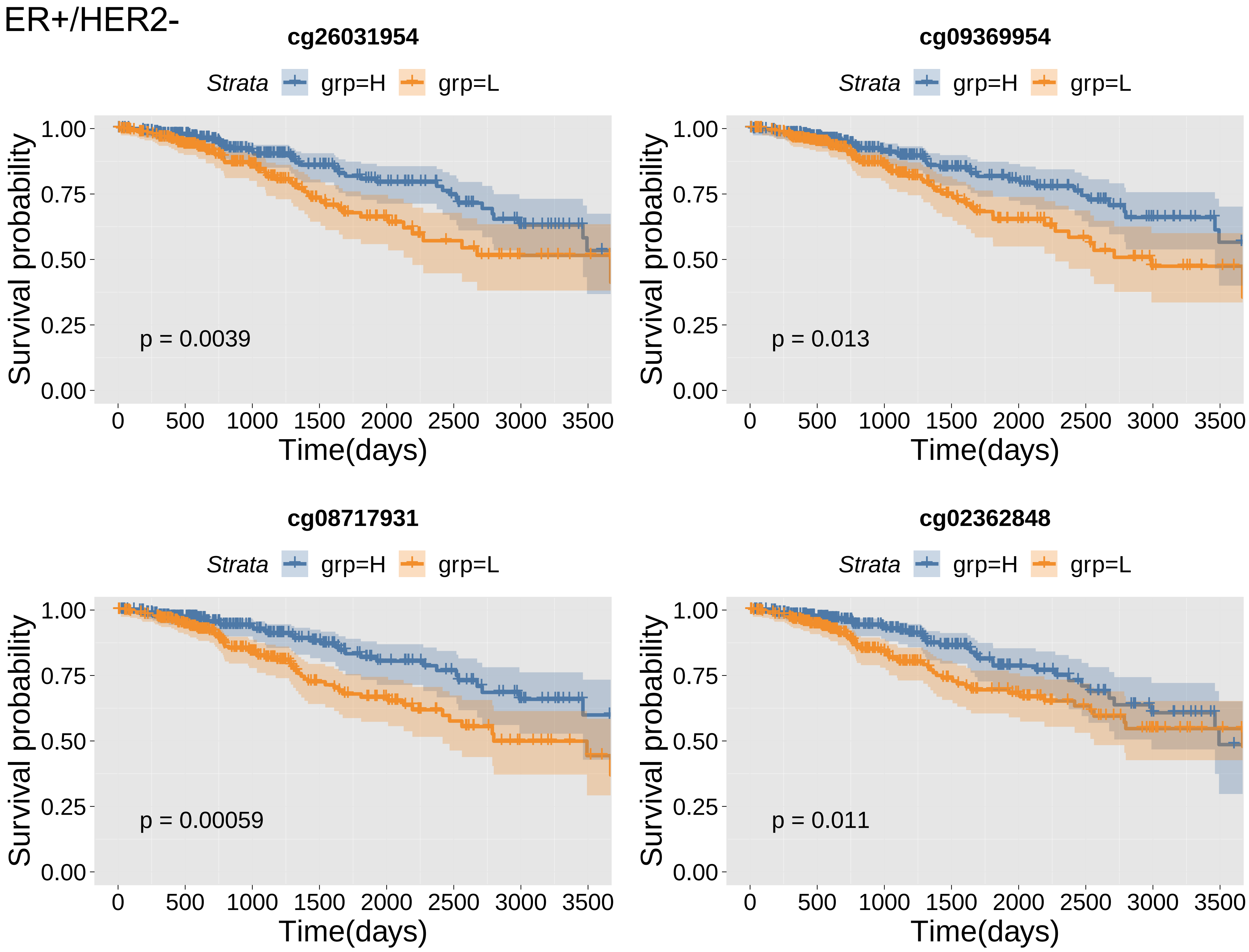

ER+/HER2-
cg26031954
Strata grp=H grp=L
Survival probability
1.00
0.75
0.50
0.25
0.00
p = 0.0039
0 500 1000 1500 2000 2500 3000 3500
Time(days)
cg09369954
Strata grp=H grp=L
Survival probability
1.00
0.75
0.50
0.25
0.00
p = 0.013
0 500 1000 1500 2000 2500 3000 3500
Time(days)
cg08717931
Strata grp=H grp=L
Survival probability
1.00
0.75
0.50
0.25
0.00
p = 0.00059
0 500 1000 1500 2000 2500 3000 3500
Time(days)
cg02362848
Strata grp=H grp=L
Survival probability
1.00
0.75
0.50
0.25
0.00
p = 0.011
0 500 1000 1500 2000 2500 3000 3500
Time(days)

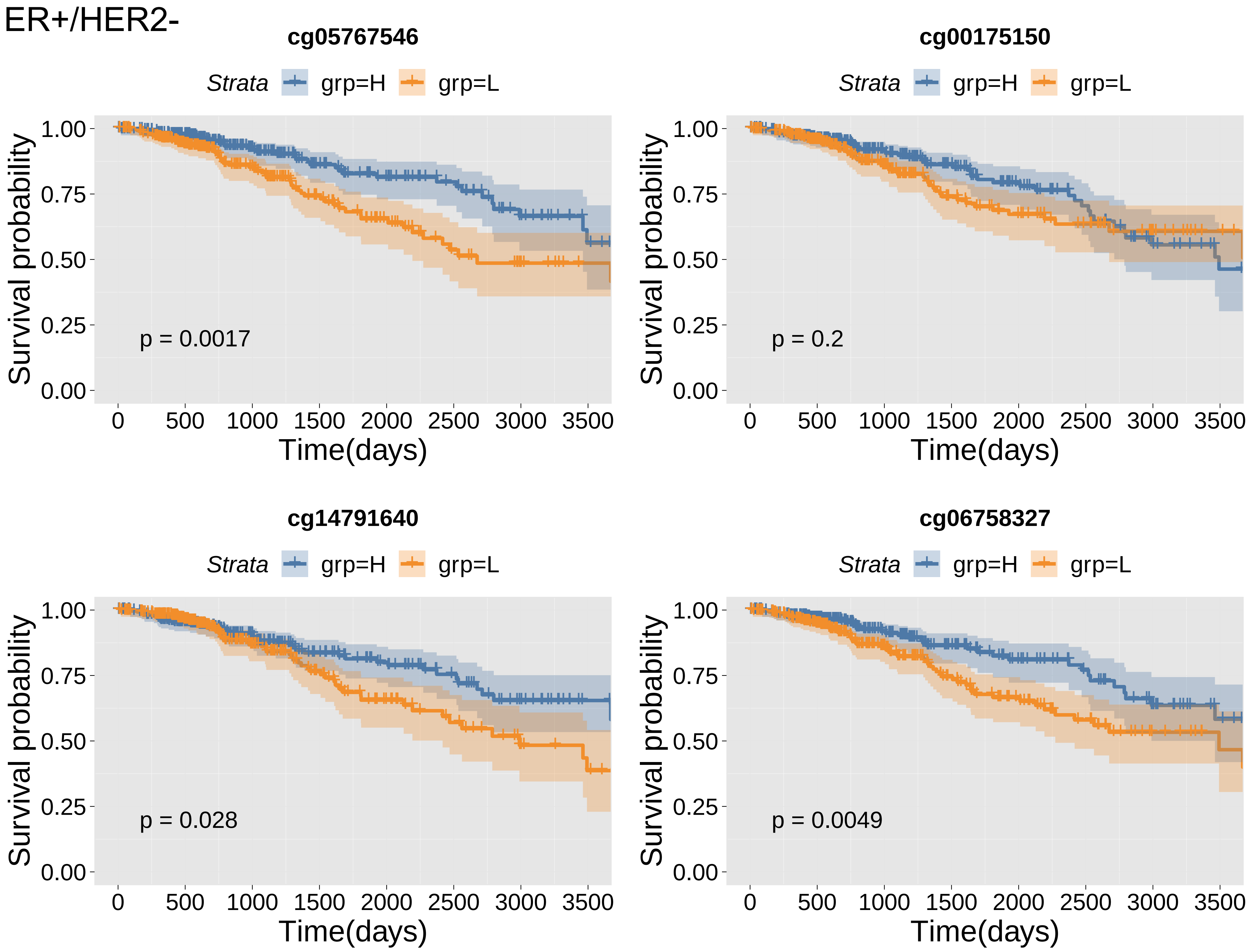
ER+/HER2-
cg05767546
Strata
grp=H
grp=L
Survival probability
1.00
0.75
0.50
0.25
0.00
p = 0.0017
0
500
1000
1500
2000
2500
3000
3500
Time(days)
cg00175150
Strata
grp=H
grp=L
Survival probability
p = 0.2
Time(days)
cg14791640
Strata
grp=H
grp=L
Survival probability
p = 0.028
Time(days)
cg06758327
Strata
grp=H
grp=L
Survival probability
p = 0.0049
Time(days)

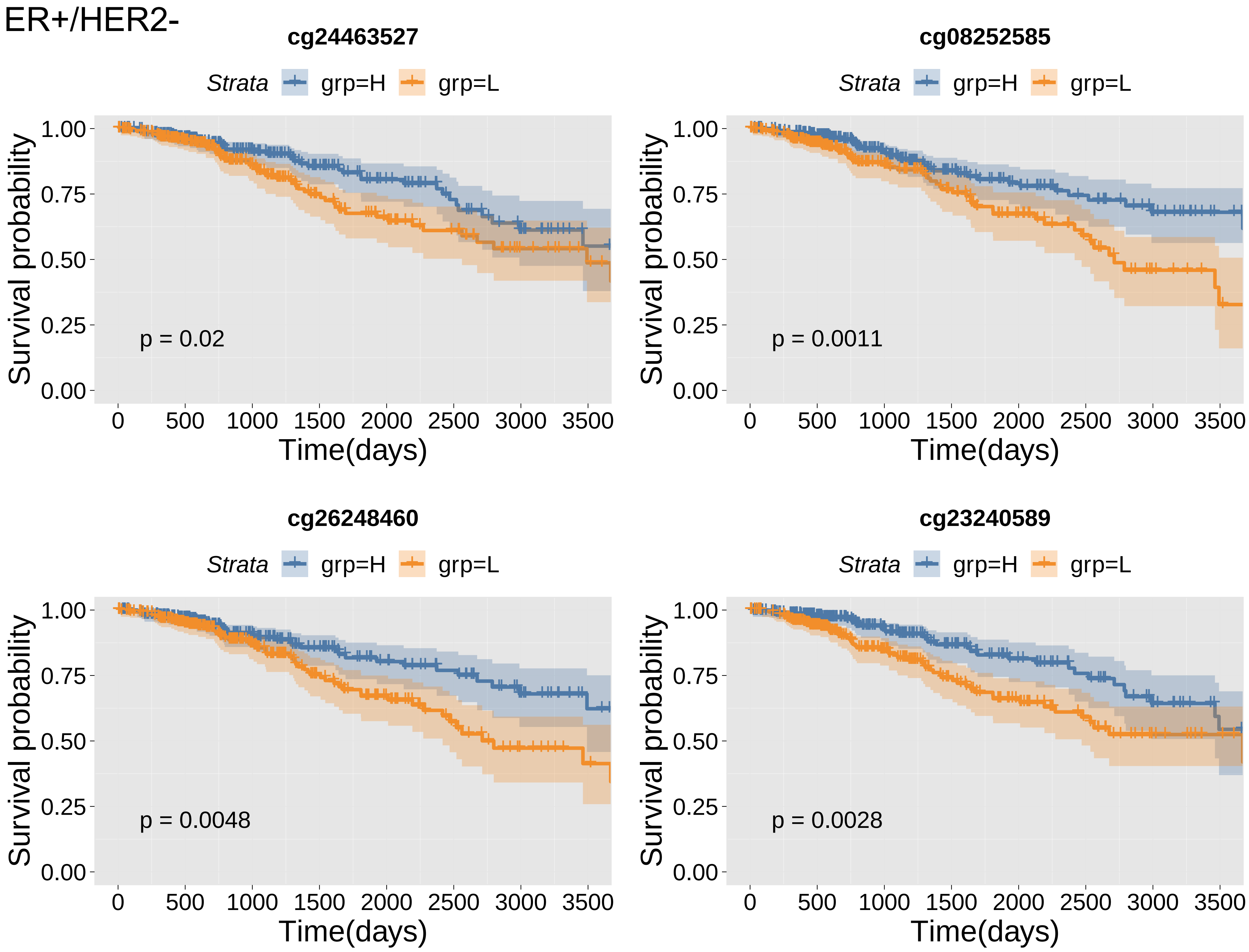

ER+/HER2-
cg24463527
Strata grp=H grp=L
Survival probability
1.00
0.75
0.50
0.25
0.00
p = 0.02
0 500 1000 1500 2000 2500 3000 3500
Time(days)
cg08252585
Strata grp=H grp=L
Survival probability
1.00
0.75
0.50
0.25
0.00
p = 0.0011
0 500 1000 1500 2000 2500 3000 3500
Time(days)
cg26248460
Strata grp=H grp=L
Survival probability
1.00
0.75
0.50
0.25
0.00
p = 0.0048
0 500 1000 1500 2000 2500 3000 3500
Time(days)
cg23240589
Strata grp=H grp=L
Survival probability
1.00
0.75
0.50
0.25
0.00
p = 0.0028
0 500 1000 1500 2000 2500 3000 3500
Time(days)

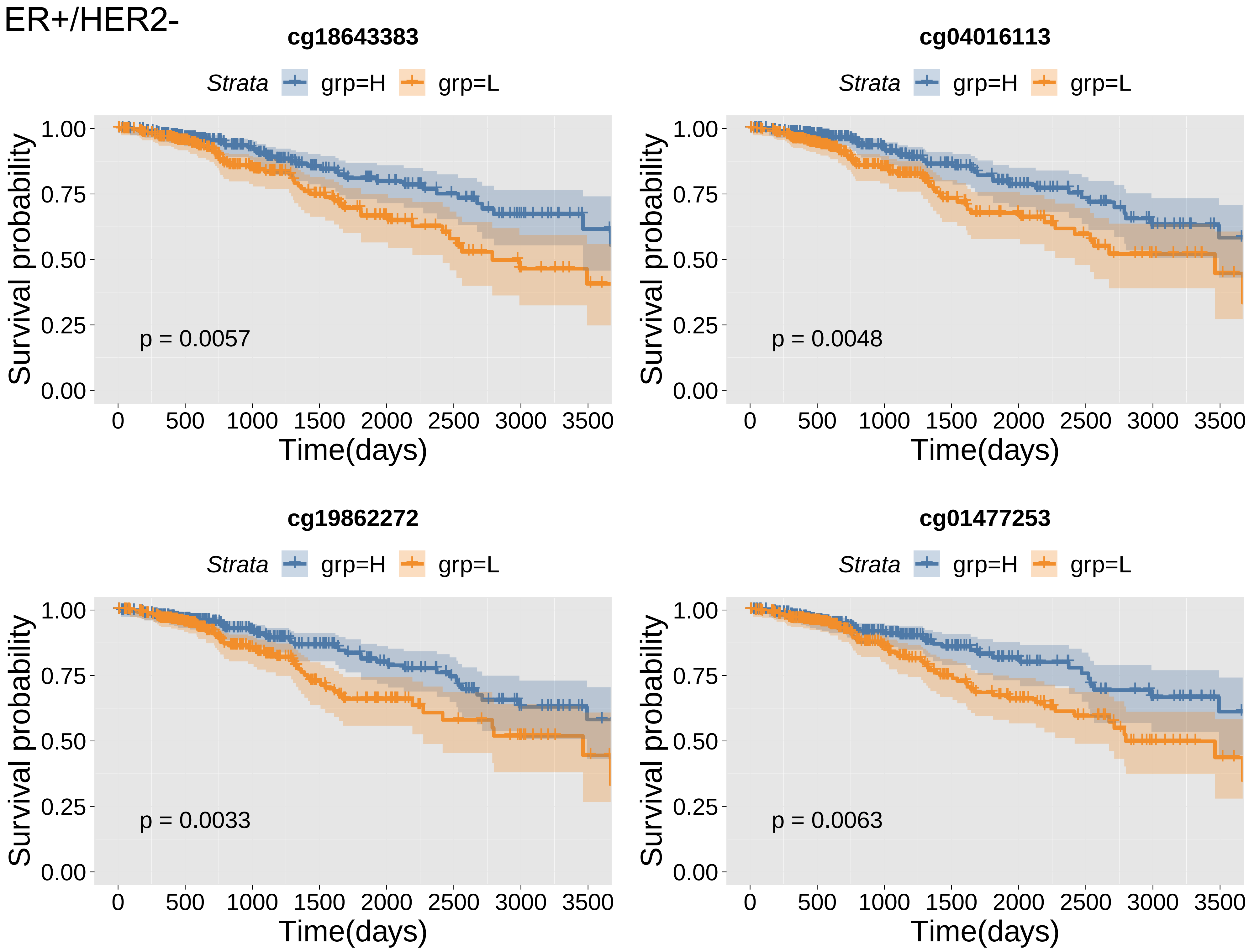
ER+/HER2-
cg18643383
Strata grp=H grp=L
Survival probability
1.00
0.75
0.50
0.25
0.00
p = 0.0057
0 500 1000 1500 2000 2500 3000 3500
Time(days)
cg04016113
Strata grp=H grp=L
Survival probability
p = 0.0048
Time(days)
cg19862272
Strata grp=H grp=L
Survival probability
p = 0.0033
Time(days)
cg01477253
Strata grp=H grp=L
Survival probability
p = 0.0063
Time(days)

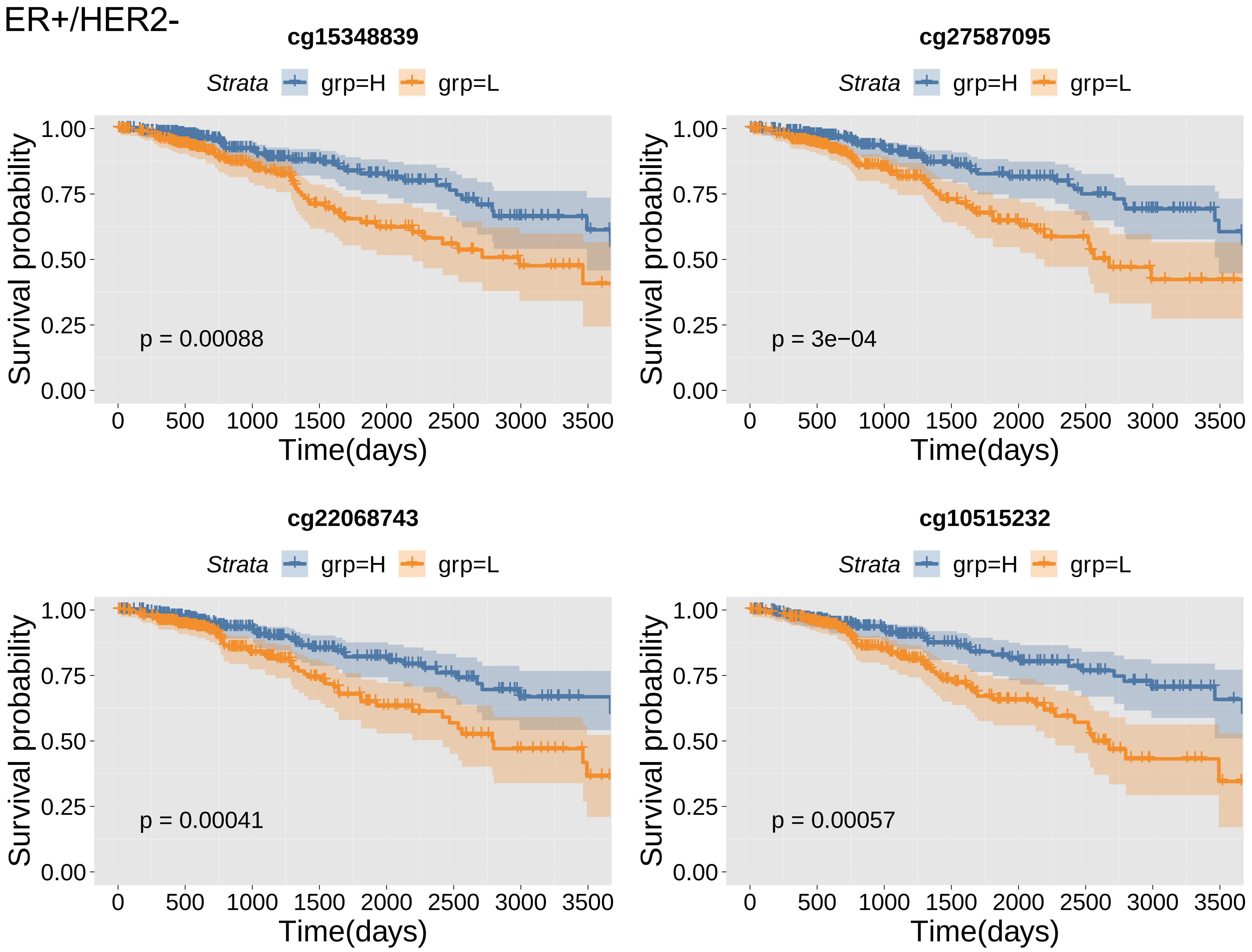
ER+/HER2-
cg15348839
Strata grp=H grp=L
Survival probability
1.00
0.75
0.50
0.25
0.00
p = 0.00088
0 500 1000 1500 2000 2500 3000 3500
Time(days)
cg27587095
Strata grp=H grp=L
Survival probability
1.00
0.75
0.50
0.25
0.00
p = 3e−04
0 500 1000 1500 2000 2500 3000 3500
Time(days)
cg22068743
Strata grp=H grp=L
Survival probability
1.00
0.75
0.50
0.25
0.00
p = 0.00041
0 500 1000 1500 2000 2500 3000 3500
Time(days)
cg10515232
Strata grp=H grp=L
Survival probability
1.00
0.75
0.50
0.25
0.00
p = 0.00057
0 500 1000 1500 2000 2500 3000 3500
Time(days)

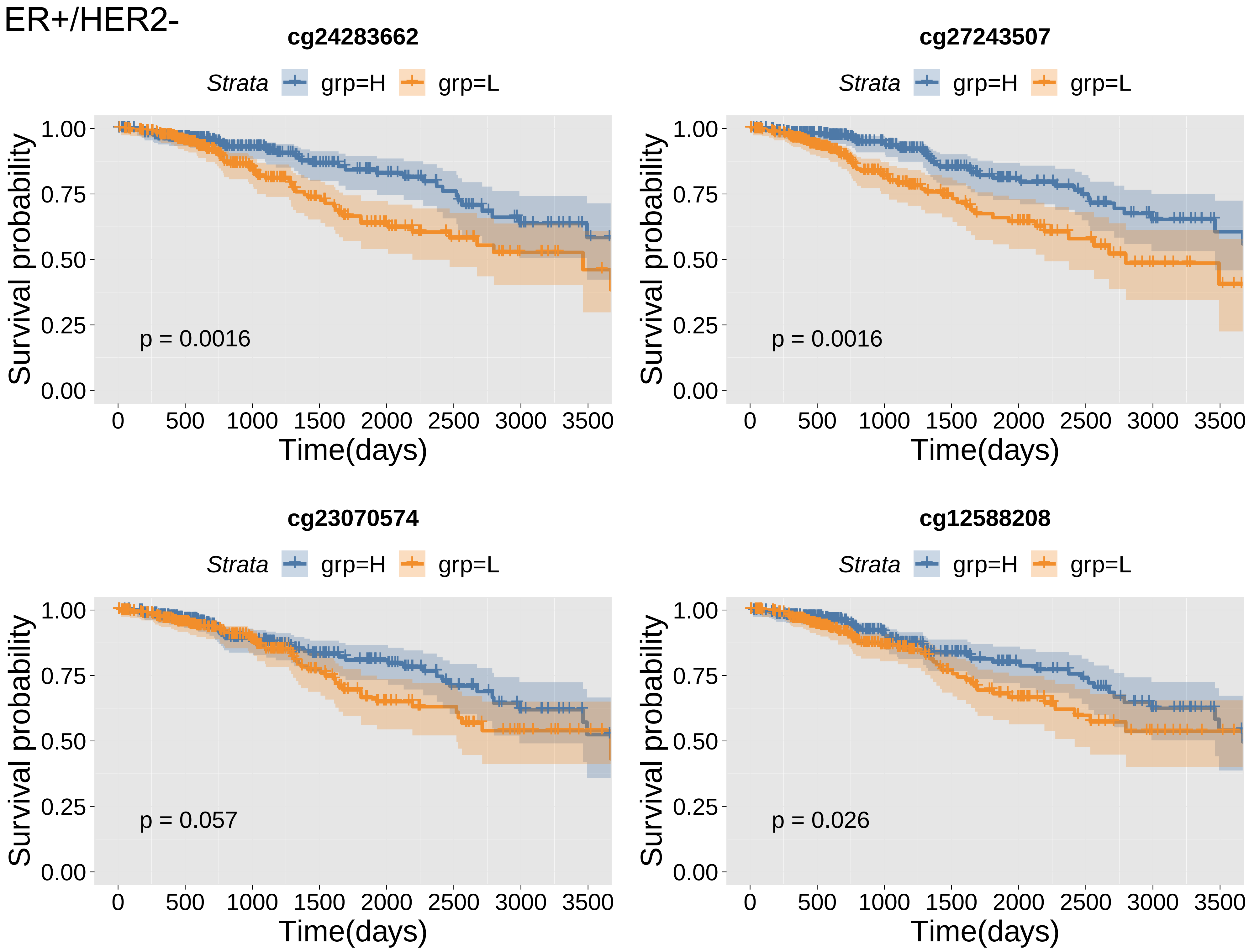

ER+/HER2-
cg24283662
cg27243507
cg23070574
cg12588208
Strata
grp=H
grp=L
Survival probability
Time(days)
p = 0.0016
p = 0.0016
p = 0.057
p = 0.026

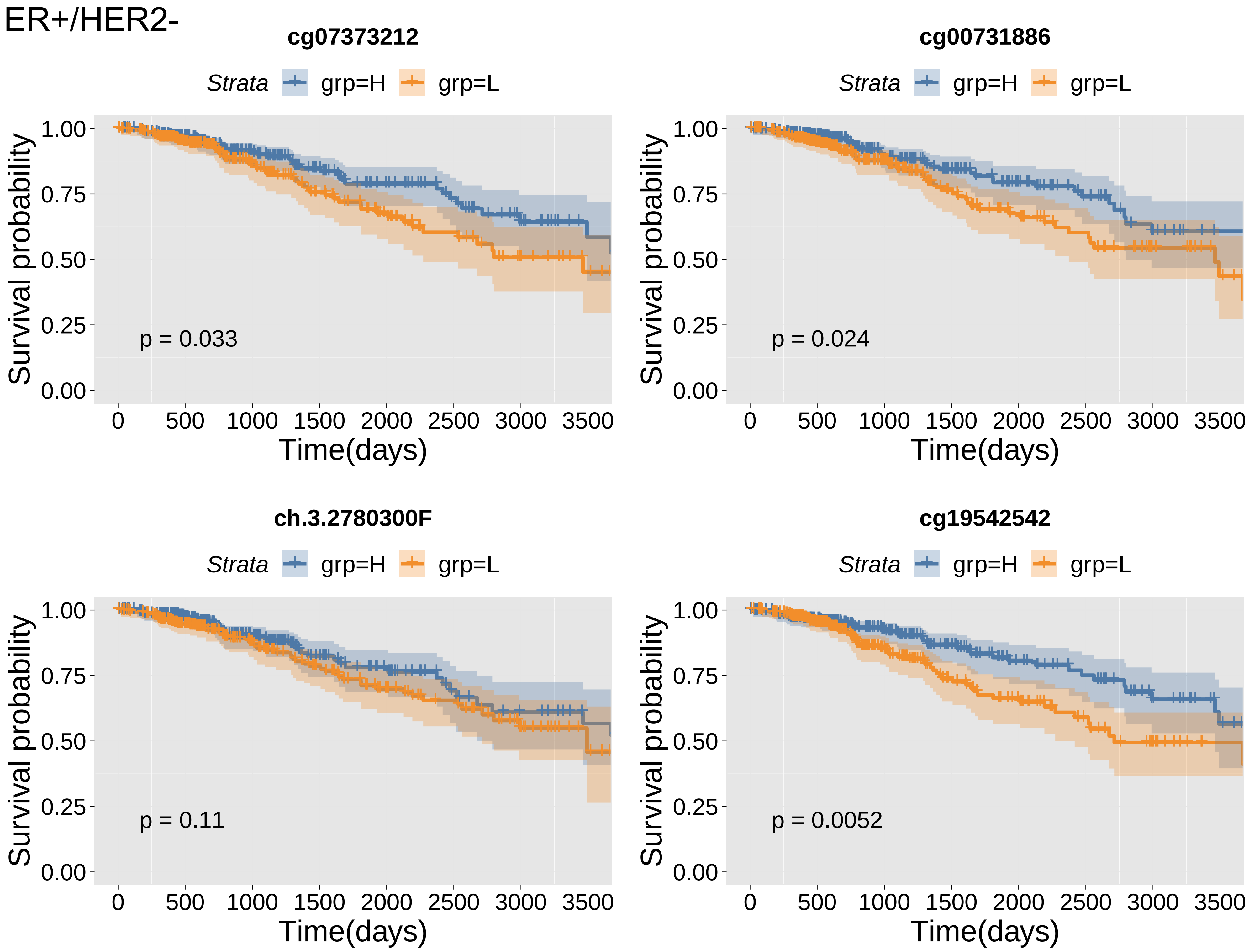
ER+/HER2-
cg07373212
Strata grp=H grp=L
Survival probability
1.00
0.75
0.50
0.25
0.00
p = 0.033
0 500 1000 1500 2000 2500 3000 3500
Time(days)
cg00731886
Strata grp=H grp=L
Survival probability
1.00
0.75
0.50
0.25
0.00
p = 0.024
0 500 1000 1500 2000 2500 3000 3500
Time(days)
ch.3.2780300F
Strata grp=H grp=L
Survival probability
1.00
0.75
0.50
0.25
0.00
p = 0.11
0 500 1000 1500 2000 2500 3000 3500
Time(days)
cg19542542
Strata grp=H grp=L
Survival probability
1.00
0.75
0.50
0.25
0.00
p = 0.0052
0 500 1000 1500 2000 2500 3000 3500
Time(days)

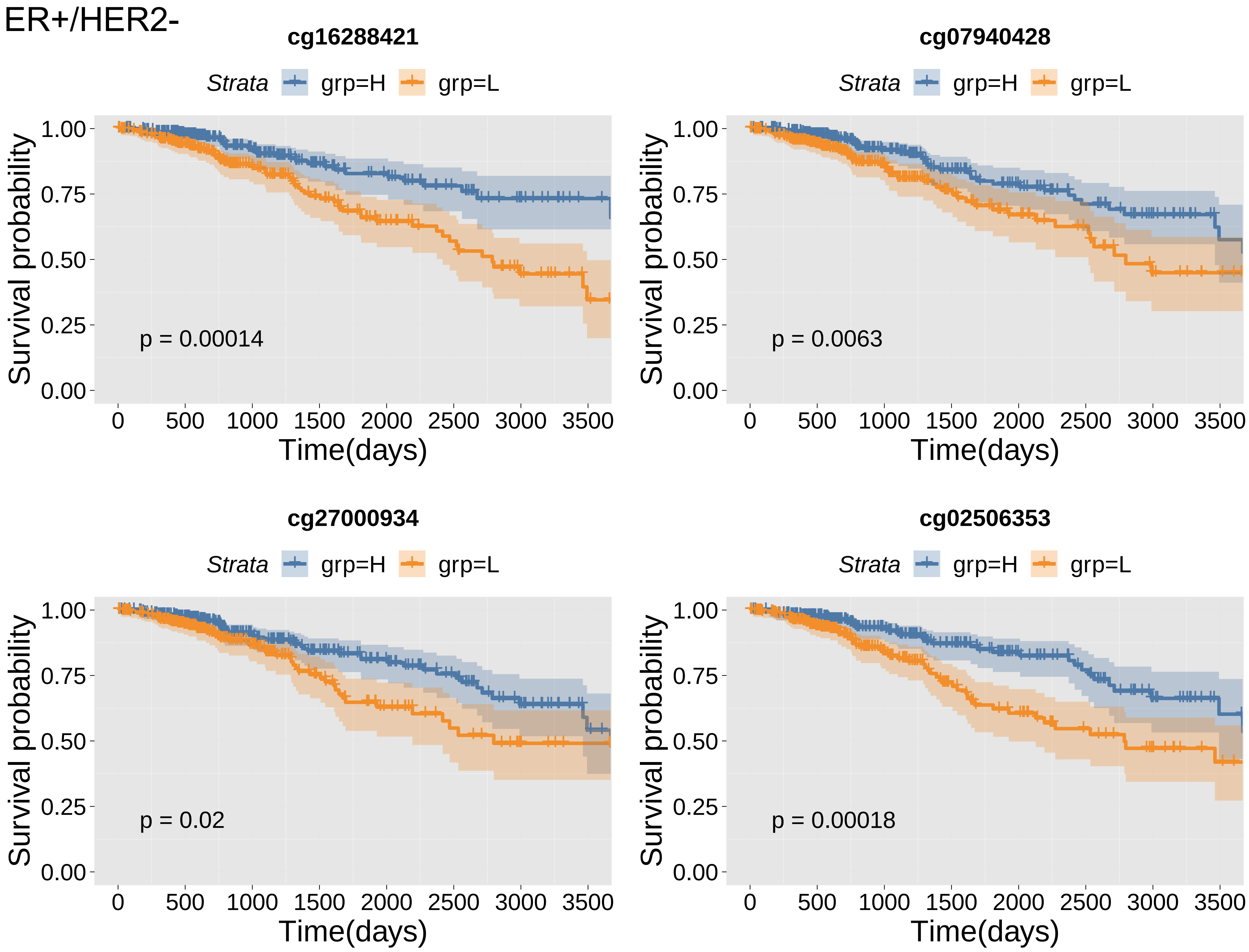
ER+/HER2-
cg16288421
Strata
grp=H
grp=L
Survival probability
1.00
0.75
0.50
0.25
0.00
p = 0.00014
0
500
1000
1500
2000
2500
3000
3500
Time(days)
cg07940428
Strata
grp=H
grp=L
Survival probability
p = 0.0063
Time(days)
cg27000934
Strata
grp=H
grp=L
Survival probability
p = 0.02
Time(days)
cg02506353
Strata
grp=H
grp=L
Survival probability
p = 0.00018
Time(days)

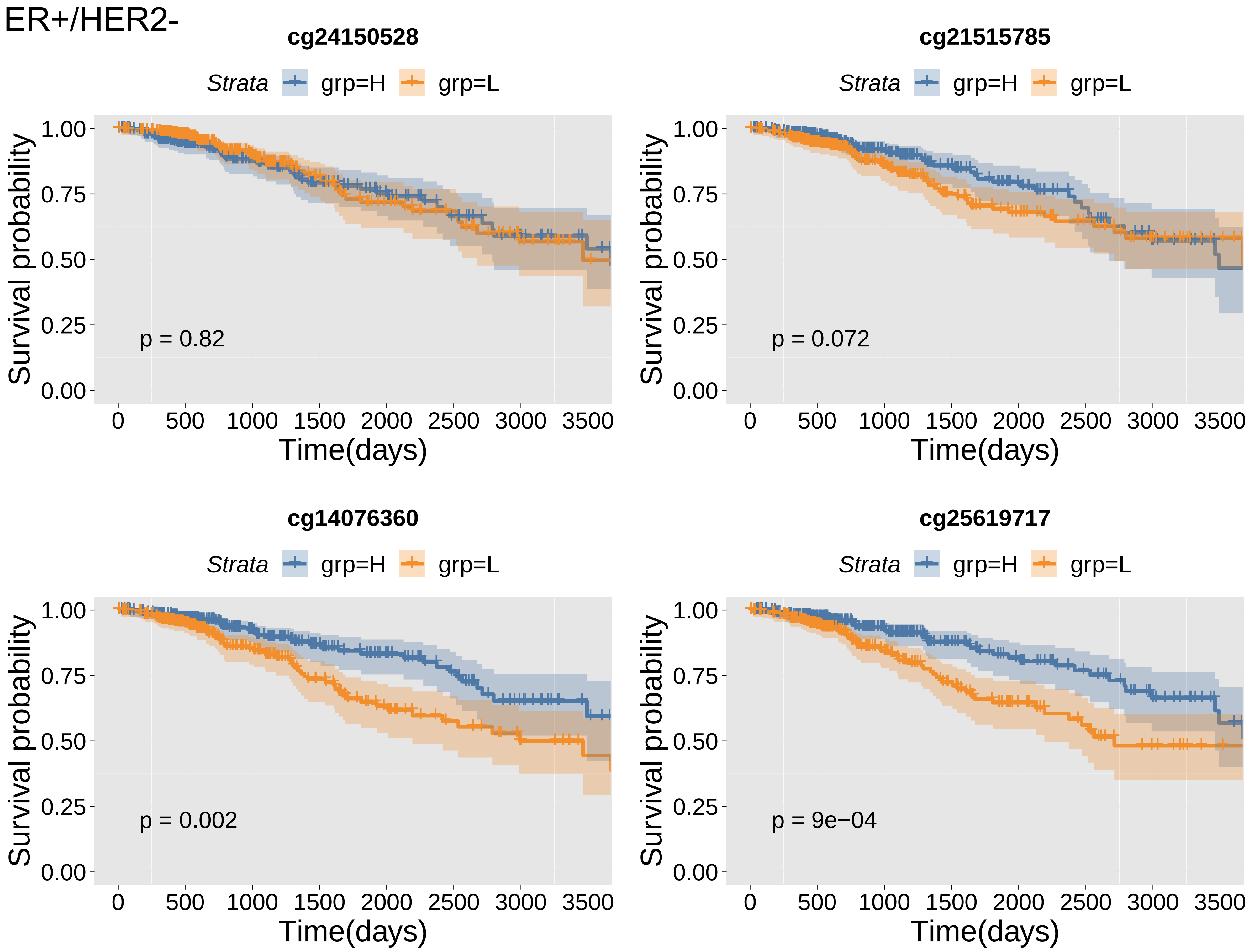
ER+/HER2-
cg24150528
Strata
grp=H
grp=L
Survival probability
1.00
0.75
0.50
0.25
0.00
p = 0.82
0
500
1000
1500
2000
2500
3000
3500
Time(days)
cg21515785
Strata
grp=H
grp=L
Survival probability
p = 0.072
Time(days)
cg14076360
Strata
grp=H
grp=L
Survival probability
p = 0.002
Time(days)
cg25619717
Strata
grp=H
grp=L
Survival probability
p = 9e−04
Time(days)

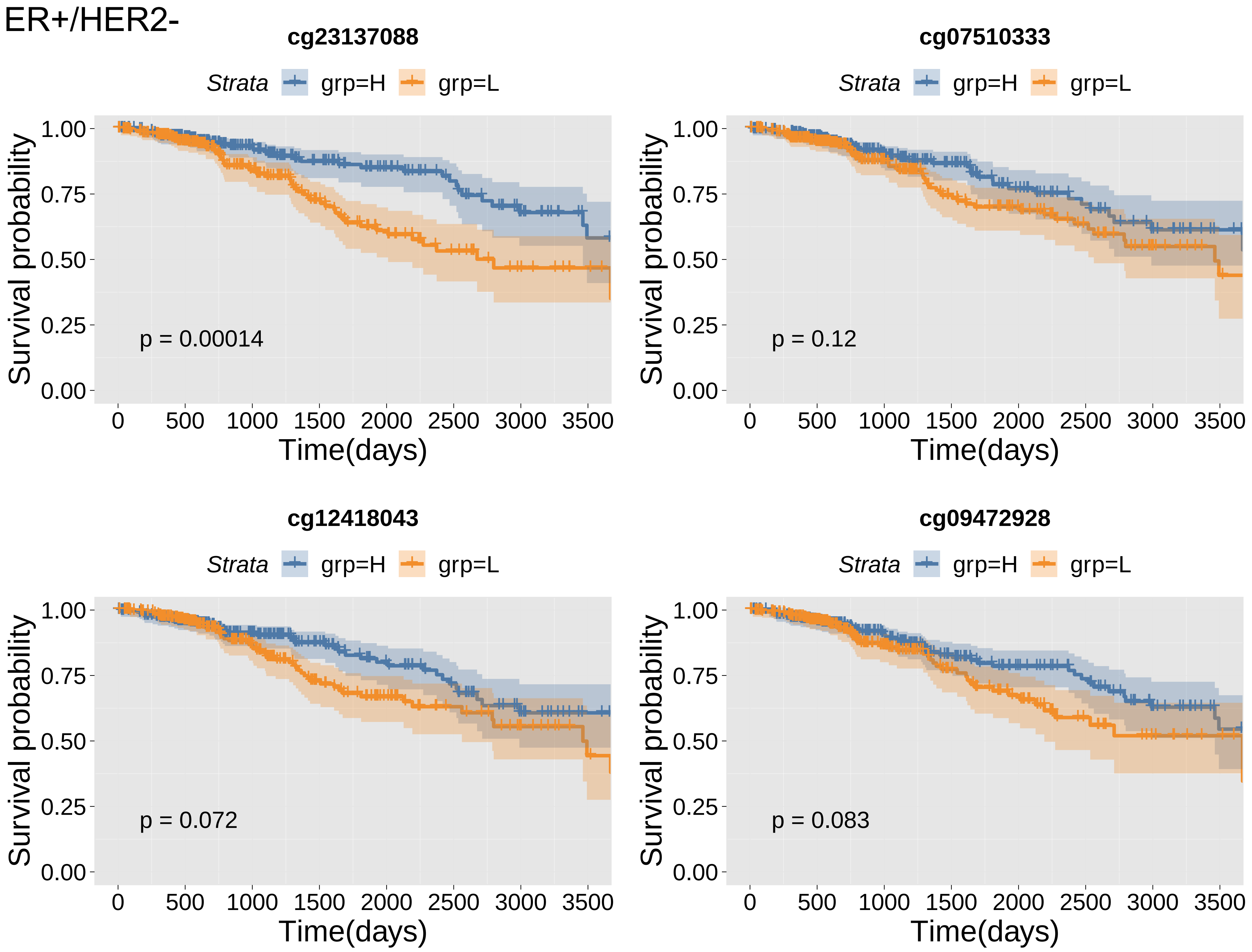
ER+/HER2-
cg23137088
Strata
grp=H
grp=L
Survival probability
1.00
0.75
0.50
0.25
0.00
p = 0.00014
0
500
1000
1500
2000
2500
3000
3500
Time(days)
cg07510333
Strata
grp=H
grp=L
Survival probability
1.00
0.75
0.50
0.25
0.00
p = 0.12
0
500
1000
1500
2000
2500
3000
3500
Time(days)
cg12418043
Strata
grp=H
grp=L
Survival probability
1.00
0.75
0.50
0.25
0.00
p = 0.072
0
500
1000
1500
2000
2500
3000
3500
Time(days)
cg09472928
Strata
grp=H
grp=L
Survival probability
1.00
0.75
0.50
0.25
0.00
p = 0.083
0
500
1000
1500
2000
2500
3000
3500
Time(days)

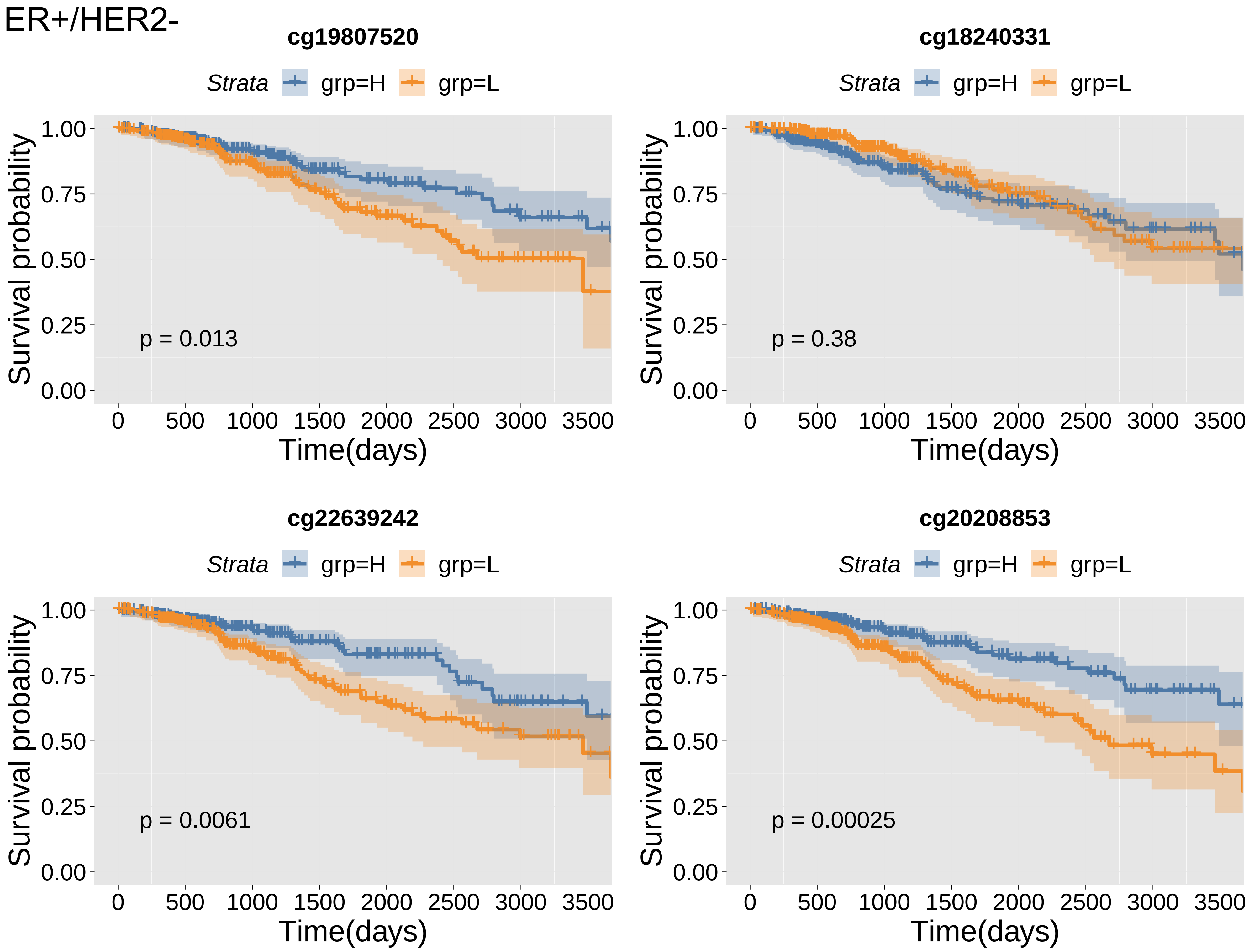
ER+/HER2-
cg19807520
Strata grp=H grp=L
Survival probability
1.00
0.75
0.50
0.25
0.00
p = 0.013
0 500 1000 1500 2000 2500 3000 3500
Time(days)
cg18240331
Strata grp=H grp=L
Survival probability
1.00
0.75
0.50
0.25
0.00
p = 0.38
0 500 1000 1500 2000 2500 3000 3500
Time(days)
cg22639242
Strata grp=H grp=L
Survival probability
1.00
0.75
0.50
0.25
0.00
p = 0.0061
0 500 1000 1500 2000 2500 3000 3500
Time(days)
cg20208853
Strata grp=H grp=L
Survival probability
1.00
0.75
0.50
0.25
0.00
p = 0.00025
0 500 1000 1500 2000 2500 3000 3500
Time(days)

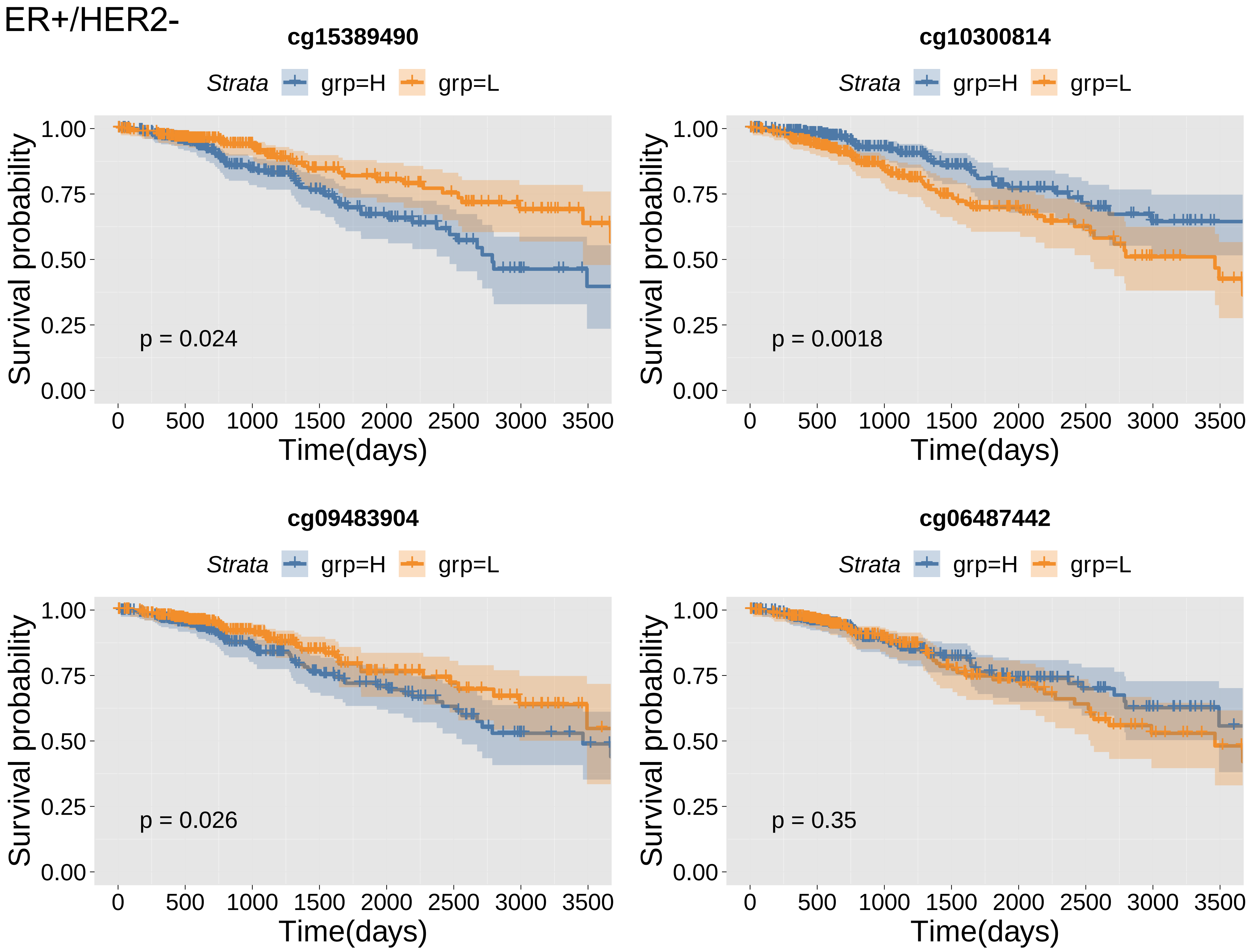

ER+/HER2-
cg15389490
Strata grp=H grp=L
Survival probability
1.00
0.75
0.50
0.25
0.00
p = 0.024
0 500 1000 1500 2000 2500 3000 3500
Time(days)
cg10300814
Strata grp=H grp=L
Survival probability
1.00
0.75
0.50
0.25
0.00
p = 0.0018
0 500 1000 1500 2000 2500 3000 3500
Time(days)
cg09483904
Strata grp=H grp=L
Survival probability
1.00
0.75
0.50
0.25
0.00
p = 0.026
0 500 1000 1500 2000 2500 3000 3500
Time(days)
cg06487442
Strata grp=H grp=L
Survival probability
1.00
0.75
0.50
0.25
0.00
p = 0.35
0 500 1000 1500 2000 2500 3000 3500
Time(days)

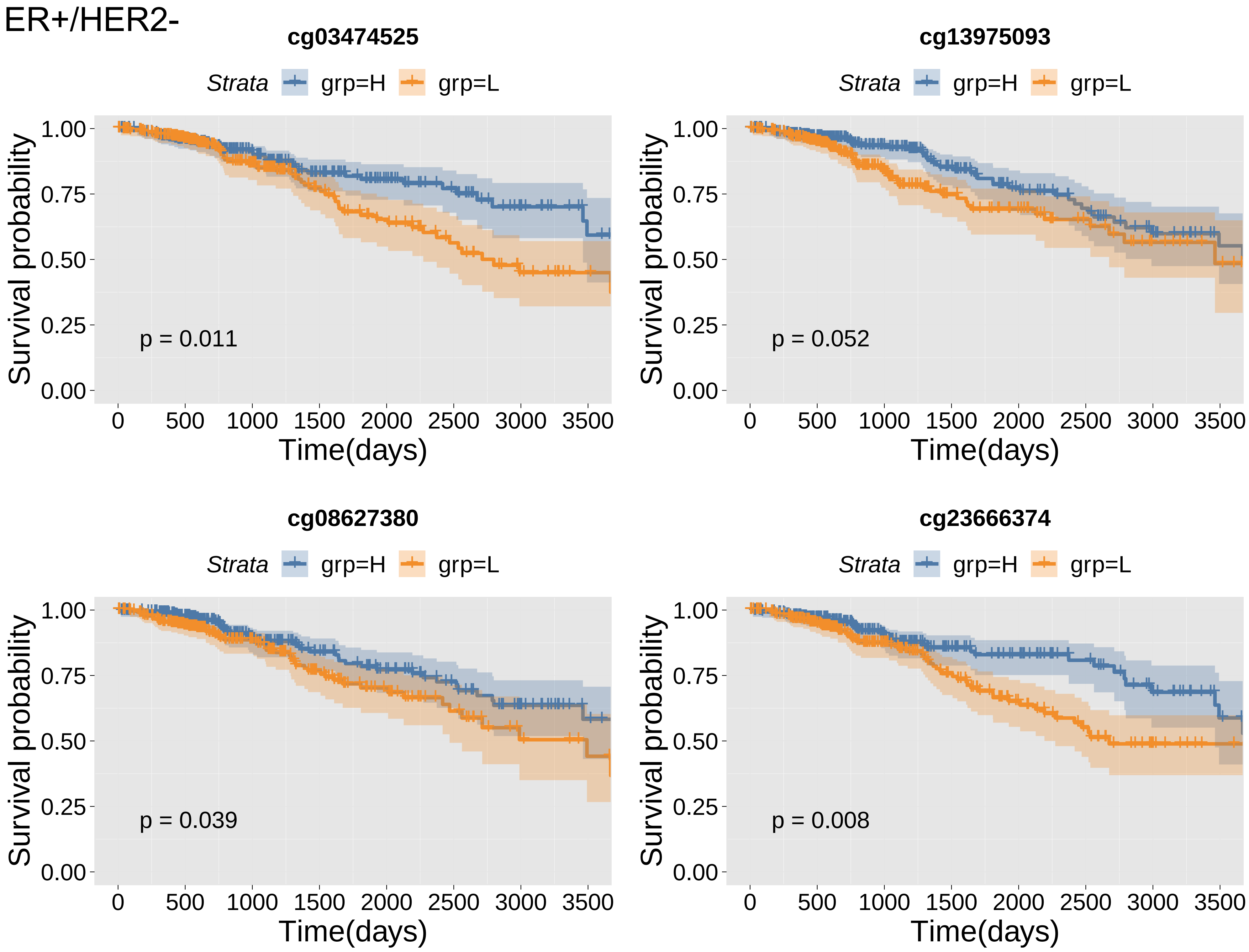

ER+/HER2-
cg03474525
Strata grp=H grp=L
Survival probability
1.00
0.75
0.50
0.25
0.00
0 500 1000 1500 2000 2500 3000 3500
Time(days)
p = 0.011
cg13975093
Strata grp=H grp=L
Survival probability
p = 0.052
Time(days)
cg08627380
Strata grp=H grp=L
Survival probability
p = 0.039
Time(days)
cg23666374
Strata grp=H grp=L
Survival probability
p = 0.008
Time(days)

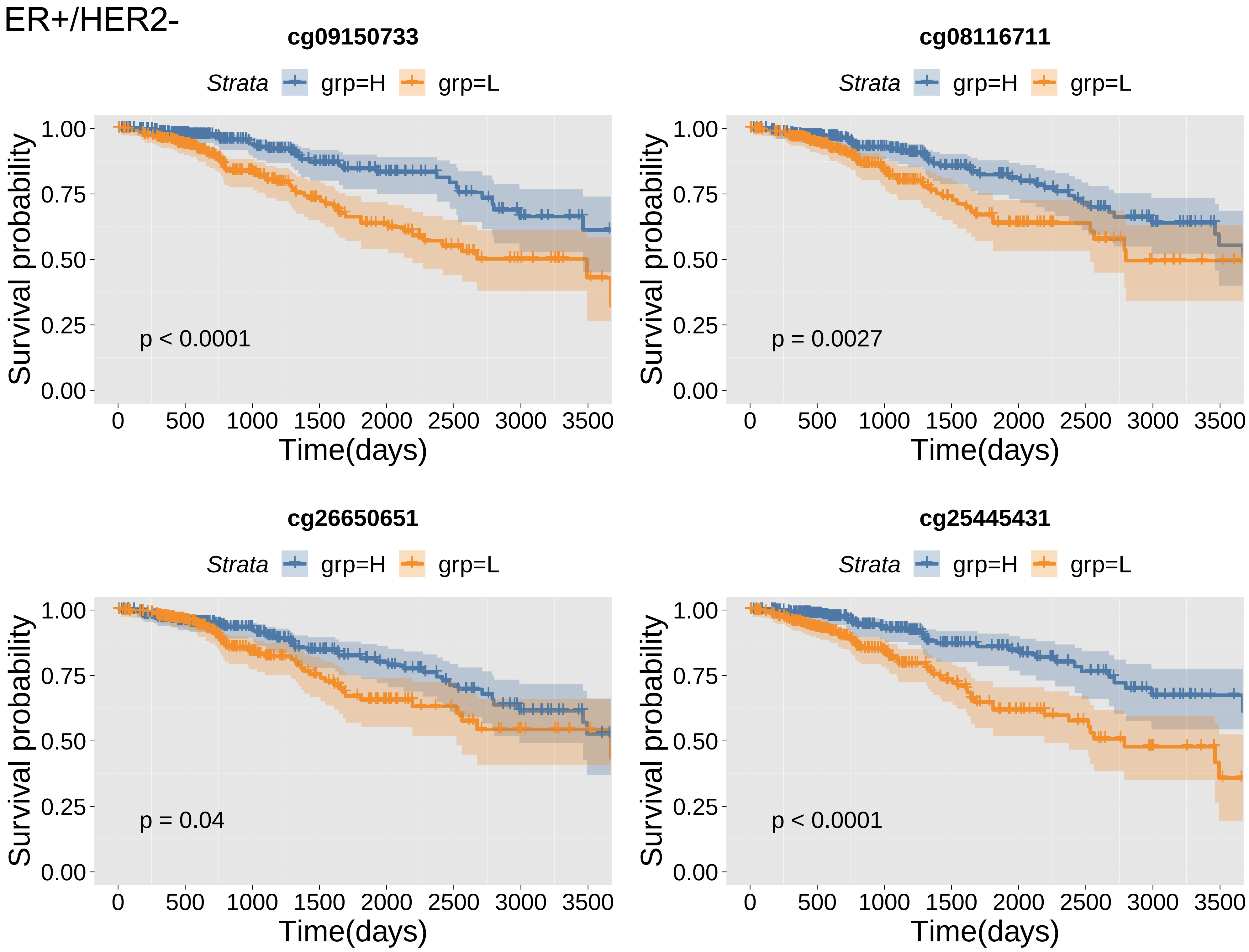
ER+/HER2-
cg09150733
Strata grp=H grp=L
Survival probability
1.00
0.75
0.50
0.25
0.00
p < 0.0001
0 500 1000 1500 2000 2500 3000 3500
Time(days)
cg08116711
Strata grp=H grp=L
Survival probability
p = 0.0027
Time(days)
cg26650651
Strata grp=H grp=L
Survival probability
p = 0.04
Time(days)
cg25445431
Strata grp=H grp=L
Survival probability
p < 0.0001
Time(days)

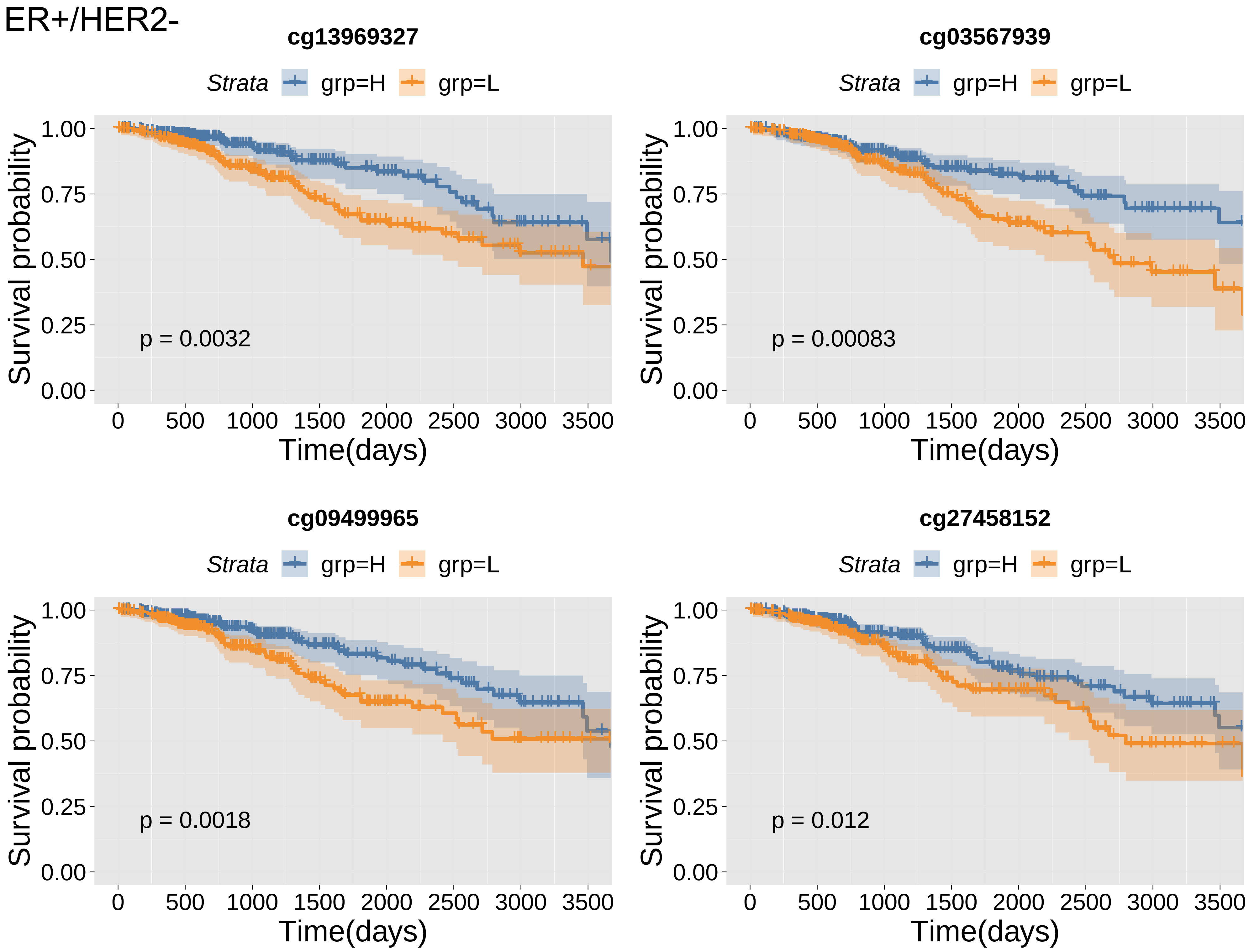

ER+/HER2-
cg13969327
Strata
grp=H
grp=L
Survival probability
1.00
0.75
0.50
0.25
0.00
p = 0.0032
0
500
1000
1500
2000
2500
3000
3500
Time(days)
cg03567939
Strata
grp=H
grp=L
Survival probability
p = 0.00083
Time(days)
cg09499965
Strata
grp=H
grp=L
Survival probability
p = 0.0018
Time(days)
cg27458152
Strata
grp=H
grp=L
Survival probability
p = 0.012
Time(days)

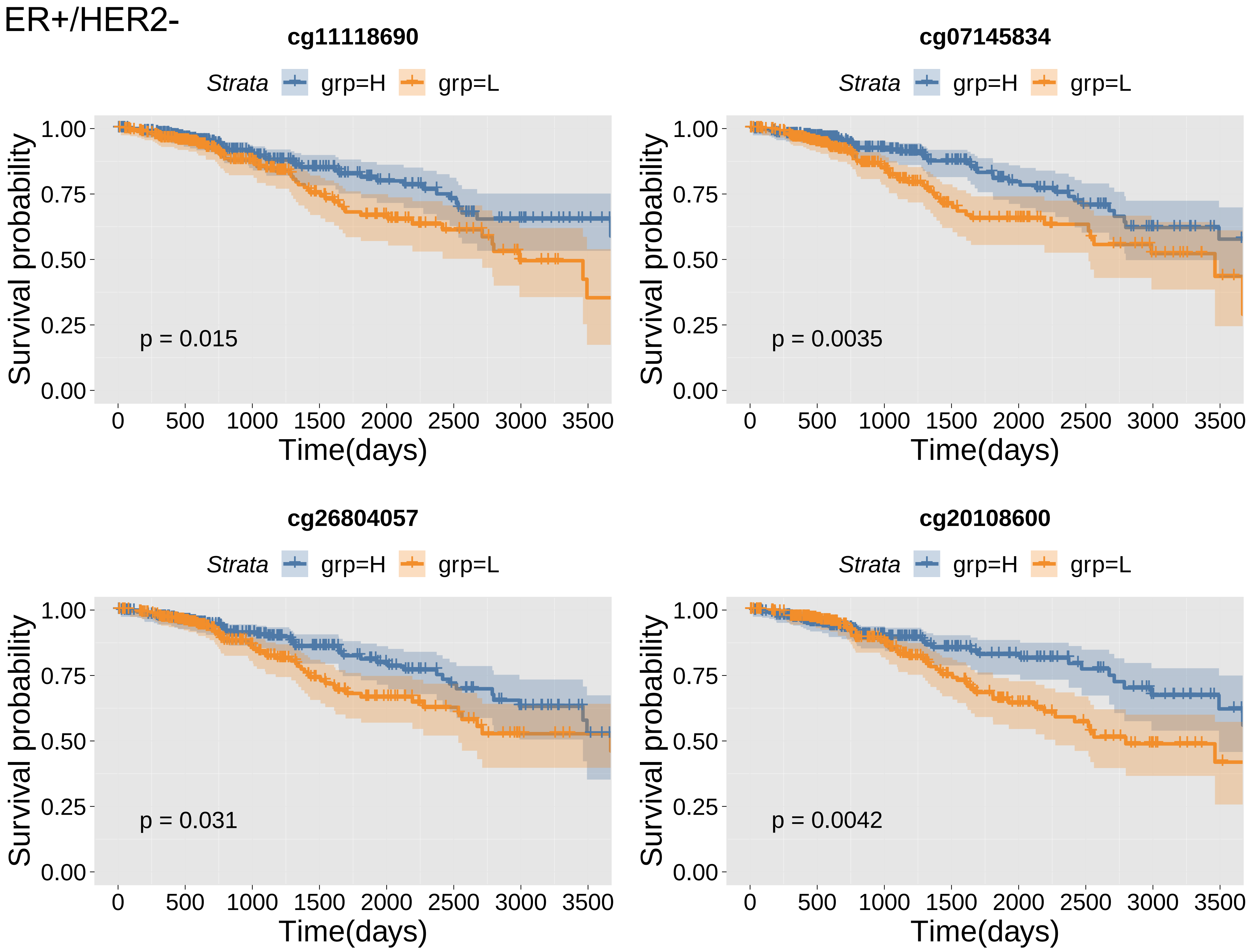

ER+/HER2-
cg11118690
Strata grp=H grp=L
Survival probability
1.00
0.75
0.50
0.25
0.00
p = 0.015
0 500 1000 1500 2000 2500 3000 3500
Time(days)
cg07145834
Strata grp=H grp=L
Survival probability
1.00
0.75
0.50
0.25
0.00
p = 0.0035
0 500 1000 1500 2000 2500 3000 3500
Time(days)
cg26804057
Strata grp=H grp=L
Survival probability
1.00
0.75
0.50
0.25
0.00
p = 0.031
0 500 1000 1500 2000 2500 3000 3500
Time(days)
cg20108600
Strata grp=H grp=L
Survival probability
1.00
0.75
0.50
0.25
0.00
p = 0.0042
0 500 1000 1500 2000 2500 3000 3500
Time(days)

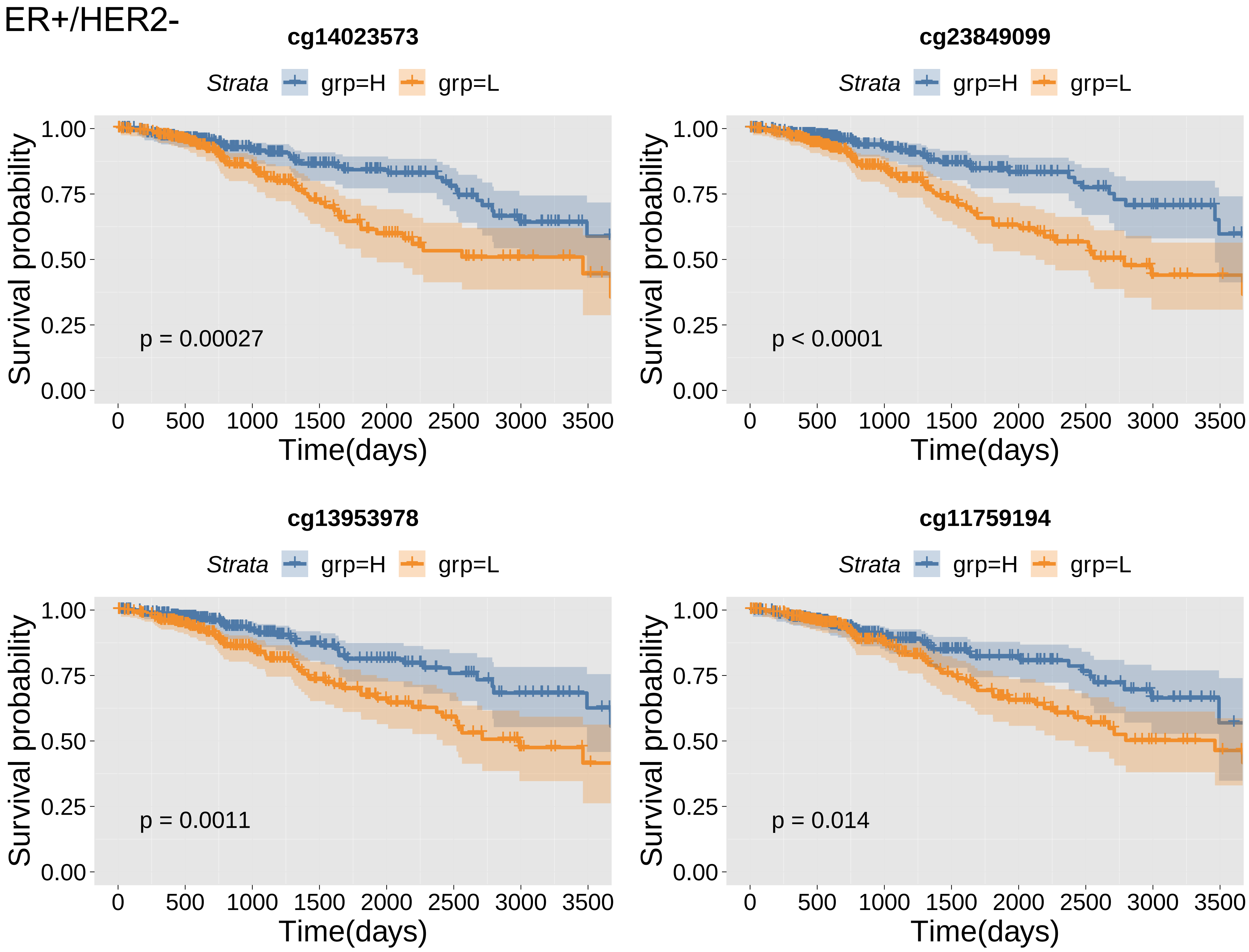
ER+/HER2-
cg14023573
Strata grp=H grp=L
Survival probability
1.00
0.75
0.50
0.25
0.00
p = 0.00027
0 500 1000 1500 2000 2500 3000 3500
Time(days)
cg23849099
Strata grp=H grp=L
Survival probability
p < 0.0001
Time(days)
cg13953978
Strata grp=H grp=L
Survival probability
p = 0.0011
Time(days)
cg11759194
Strata grp=H grp=L
Survival probability
p = 0.014
Time(days)

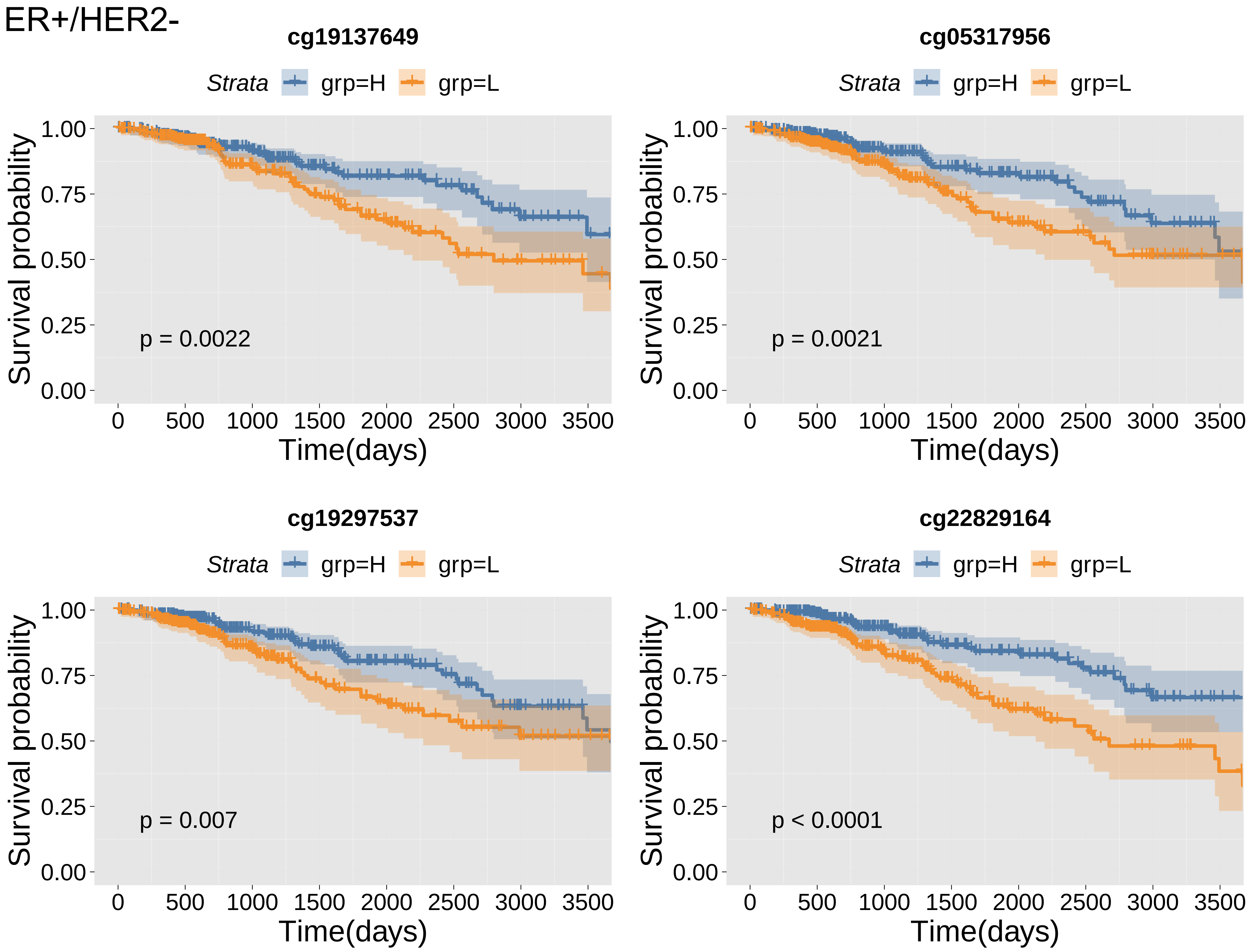
ER+/HER2-
cg19137649
Strata grp=H grp=L
Survival probability
1.00
0.75
0.50
0.25
0.00
p = 0.0022
0 500 1000 1500 2000 2500 3000 3500
Time(days)
cg05317956
Strata grp=H grp=L
Survival probability
p = 0.0021
Time(days)
cg19297537
Strata grp=H grp=L
Survival probability
p = 0.007
Time(days)
cg22829164
Strata grp=H grp=L
Survival probability
p < 0.0001
Time(days)

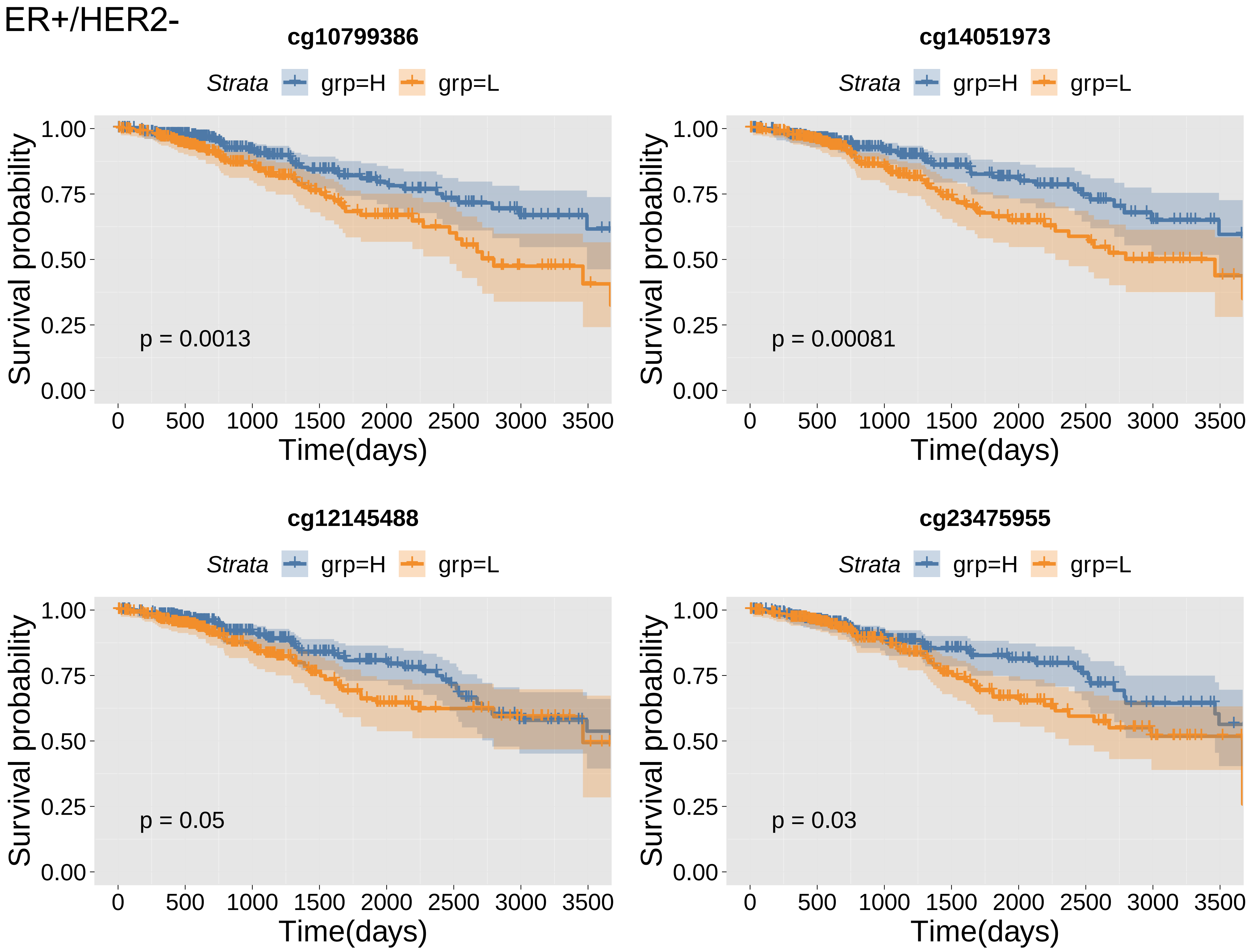
ER+/HER2-
cg10799386
Strata grp=H grp=L
Survival probability
1.00
0.75
0.50
0.25
0.00
p = 0.0013
0 500 1000 1500 2000 2500 3000 3500
Time(days)
cg14051973
Strata grp=H grp=L
Survival probability
p = 0.00081
Time(days)
cg12145488
Strata grp=H grp=L
Survival probability
p = 0.05
Time(days)
cg23475955
Strata grp=H grp=L
Survival probability
p = 0.03
Time(days)

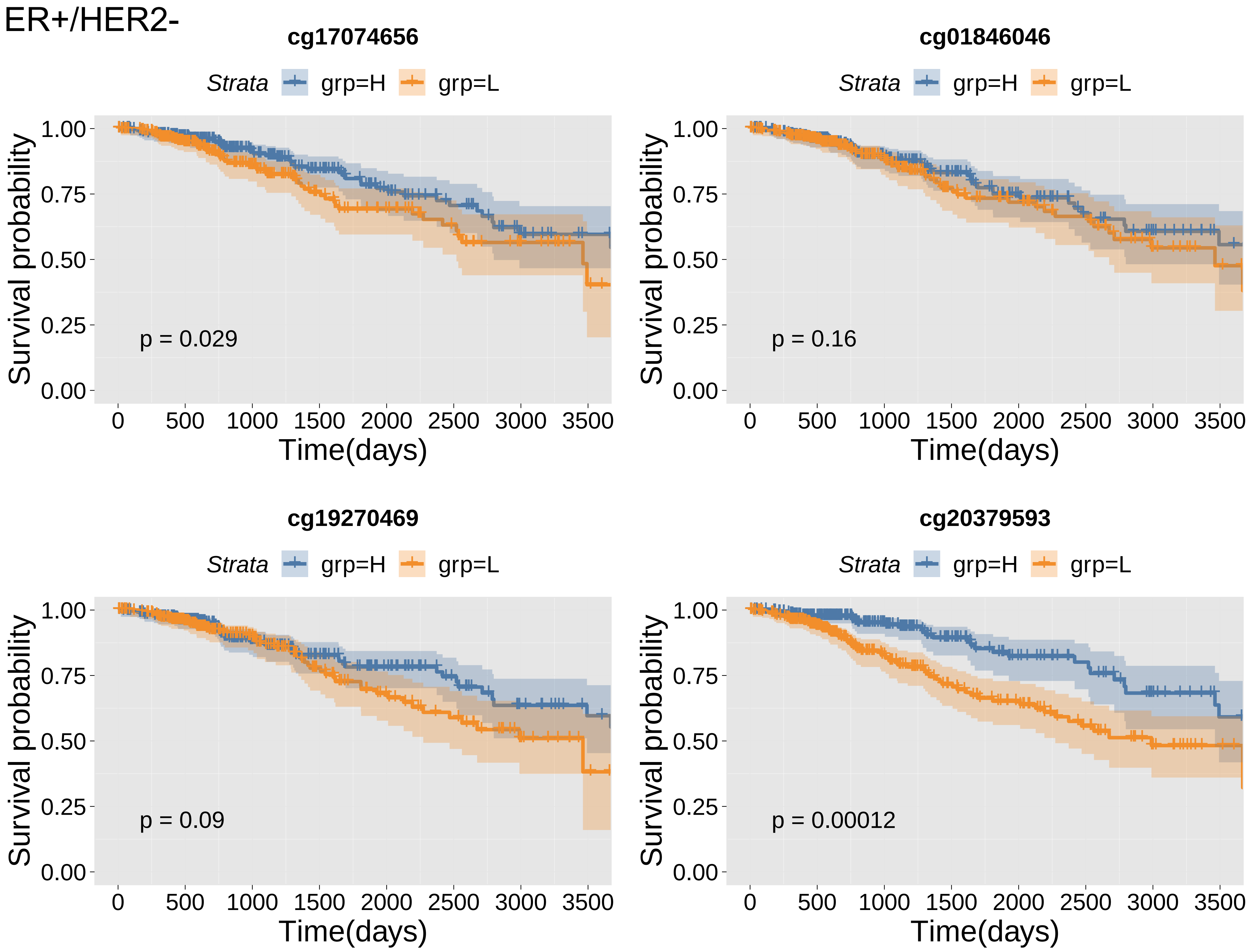
ER+/HER2-
cg17074656
Strata
grp=H
grp=L
Survival probability
1.00
0.75
0.50
0.25
0.00
p = 0.029
0
500
1000
1500
2000
2500
3000
3500
Time(days)
cg01846046
p = 0.16
cg19270469
p = 0.09
cg20379593
p = 0.00012

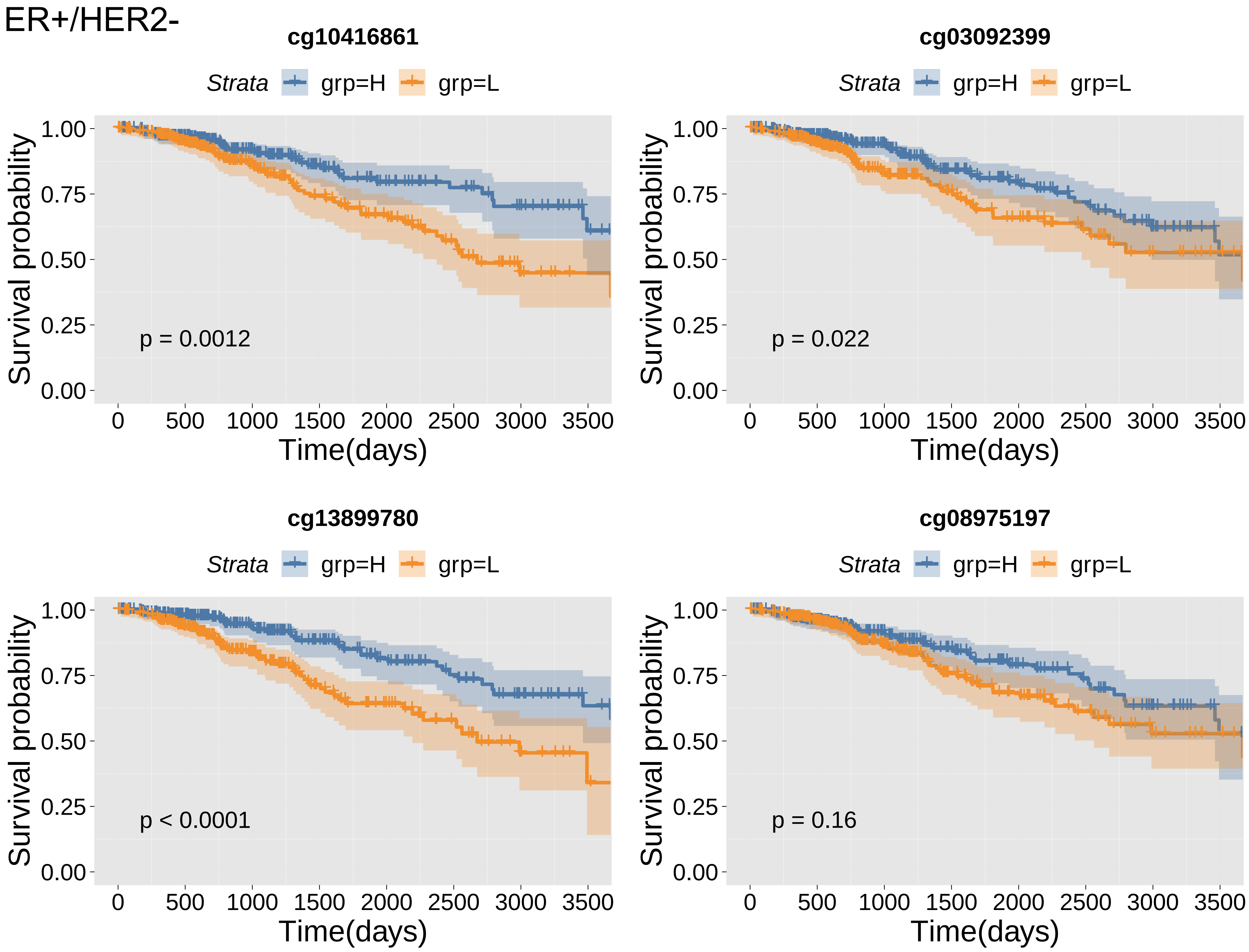

ER+/HER2-
cg10416861
Strata grp=H grp=L
Survival probability
1.00
0.75
0.50
0.25
0.00
p = 0.0012
0 500 1000 1500 2000 2500 3000 3500
Time(days)
cg03092399
Strata grp=H grp=L
Survival probability
p = 0.022
Time(days)
cg13899780
Strata grp=H grp=L
Survival probability
p < 0.0001
Time(days)
cg08975197
Strata grp=H grp=L
Survival probability
p = 0.16
Time(days)

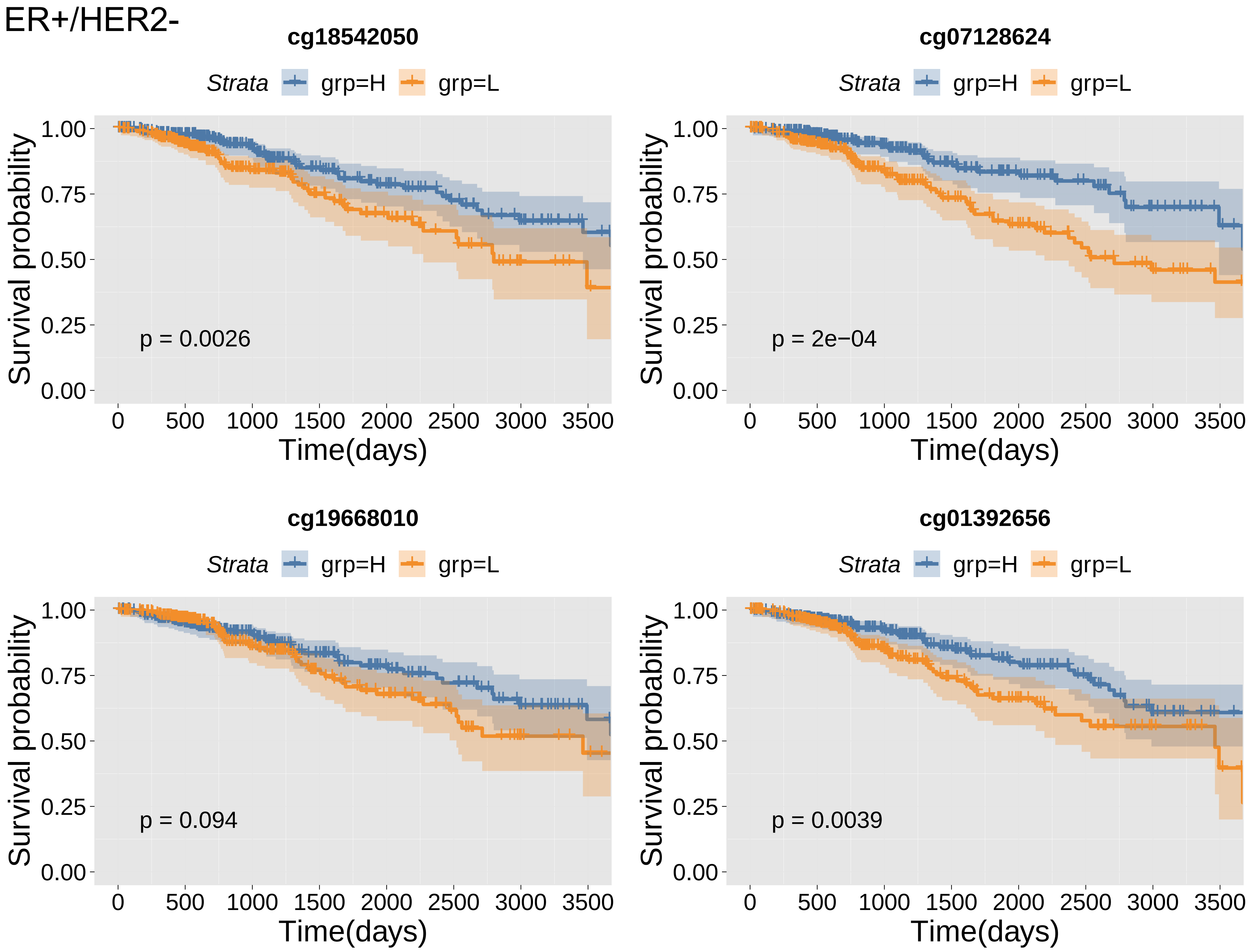
ER+/HER2-
cg18542050
Strata grp=H grp=L
Survival probability
1.00
0.75
0.50
0.25
0.00
p = 0.0026
0 500 1000 1500 2000 2500 3000 3500
Time(days)
cg07128624
Strata grp=H grp=L
Survival probability
p = 2e−04
Time(days)
cg19668010
Strata grp=H grp=L
Survival probability
p = 0.094
Time(days)
cg01392656
Strata grp=H grp=L
Survival probability
p = 0.0039
Time(days)

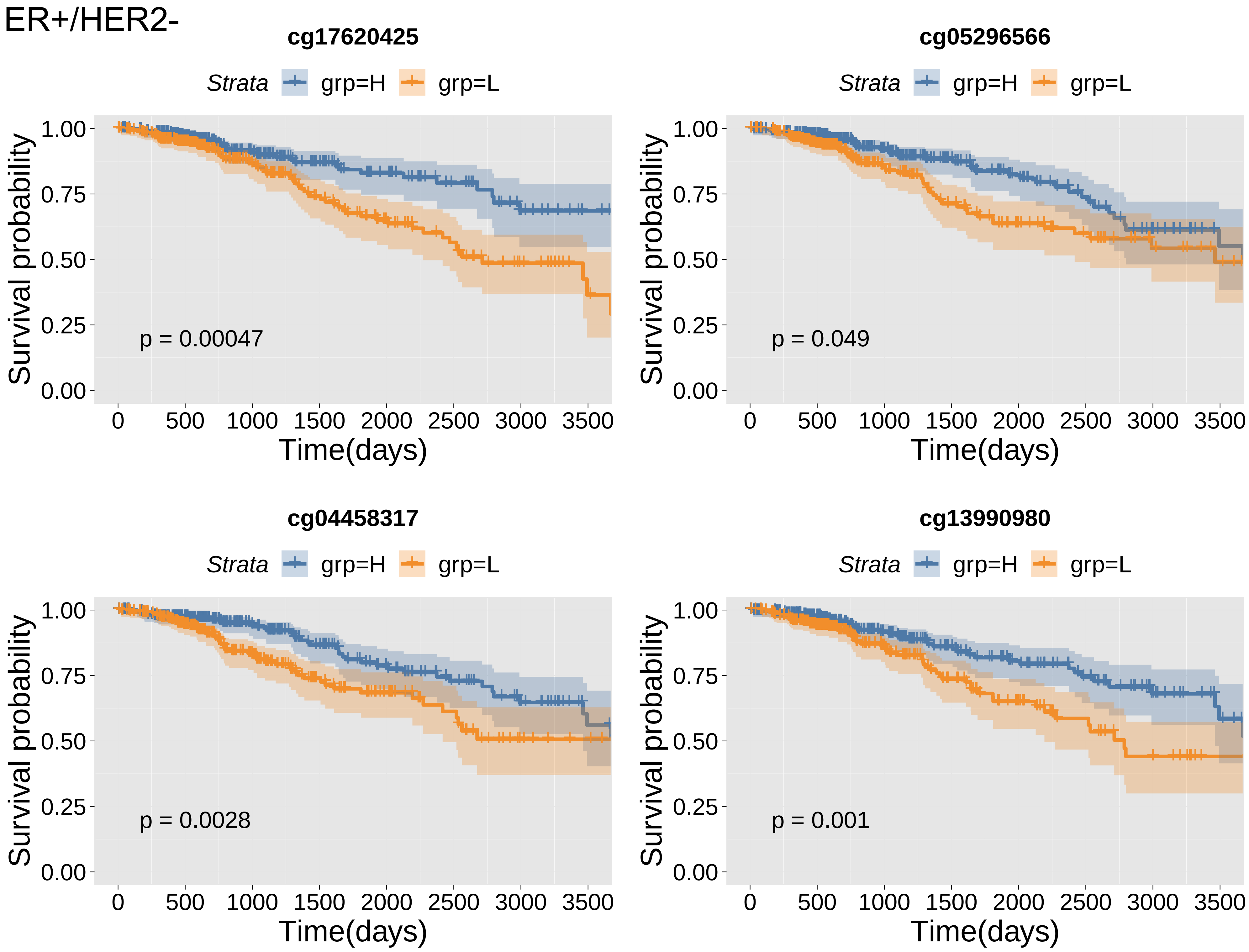
ER+/HER2-
cg17620425
Strata grp=H grp=L
Survival probability
1.00
0.75
0.50
0.25
0.00
p = 0.00047
0 500 1000 1500 2000 2500 3000 3500
Time(days)
cg05296566
Strata grp=H grp=L
p = 0.049
cg04458317
Strata grp=H grp=L
p = 0.0028
cg13990980
Strata grp=H grp=L
p = 0.001

ER+/HER2-

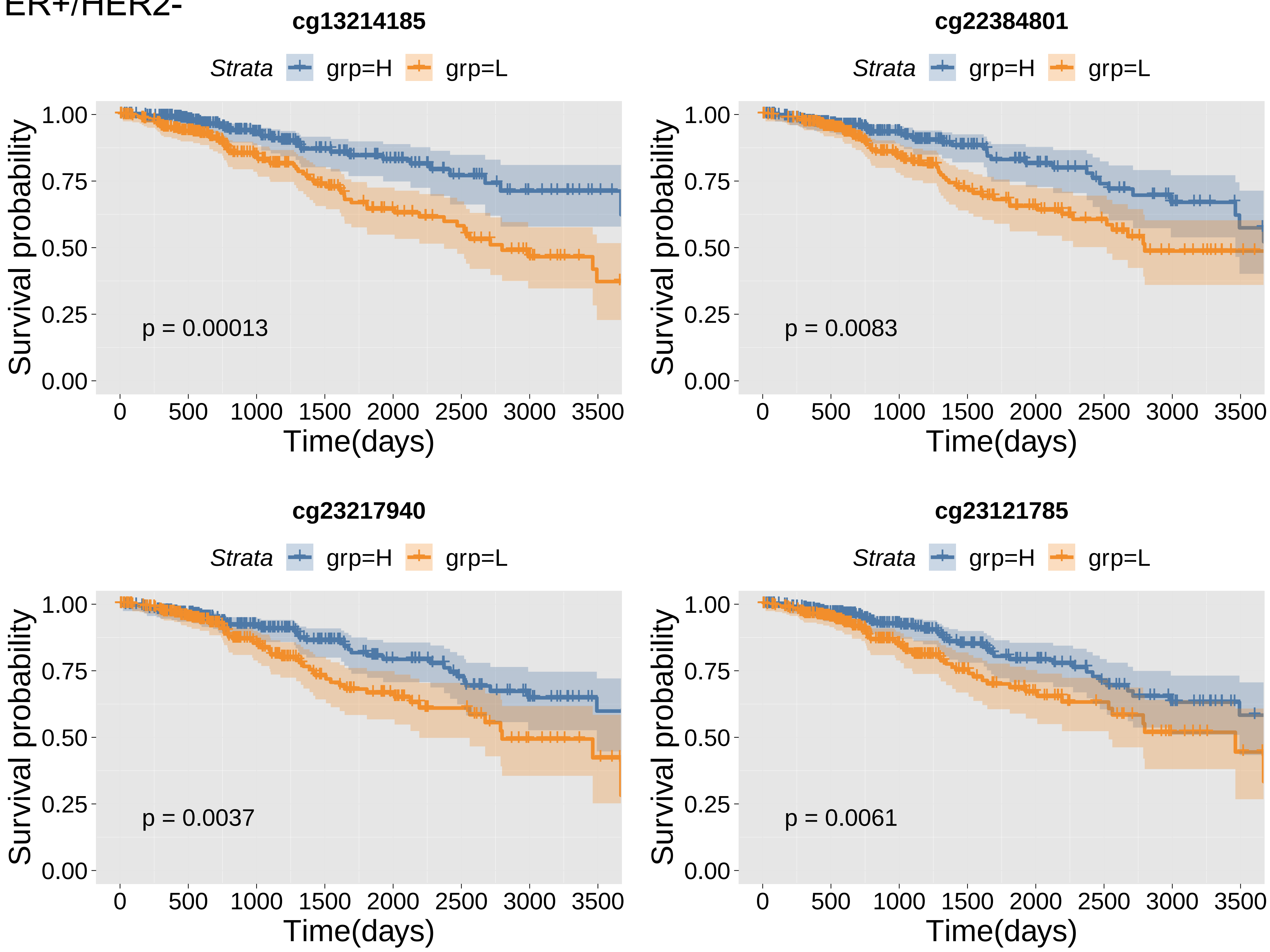
cg13214185
Strata grp=H grp=L
Survival probability
1.00
0.75
0.50
0.25
0.00
p = 0.00013
0 500 1000 1500 2000 2500 3000 3500
Time(days)
cg22384801
Strata grp=H grp=L
Survival probability
p = 0.0083
Time(days)
cg23217940
Strata grp=H grp=L
Survival probability
p = 0.0037
Time(days)
cg23121785
Strata grp=H grp=L
Survival probability
p = 0.0061
Time(days)

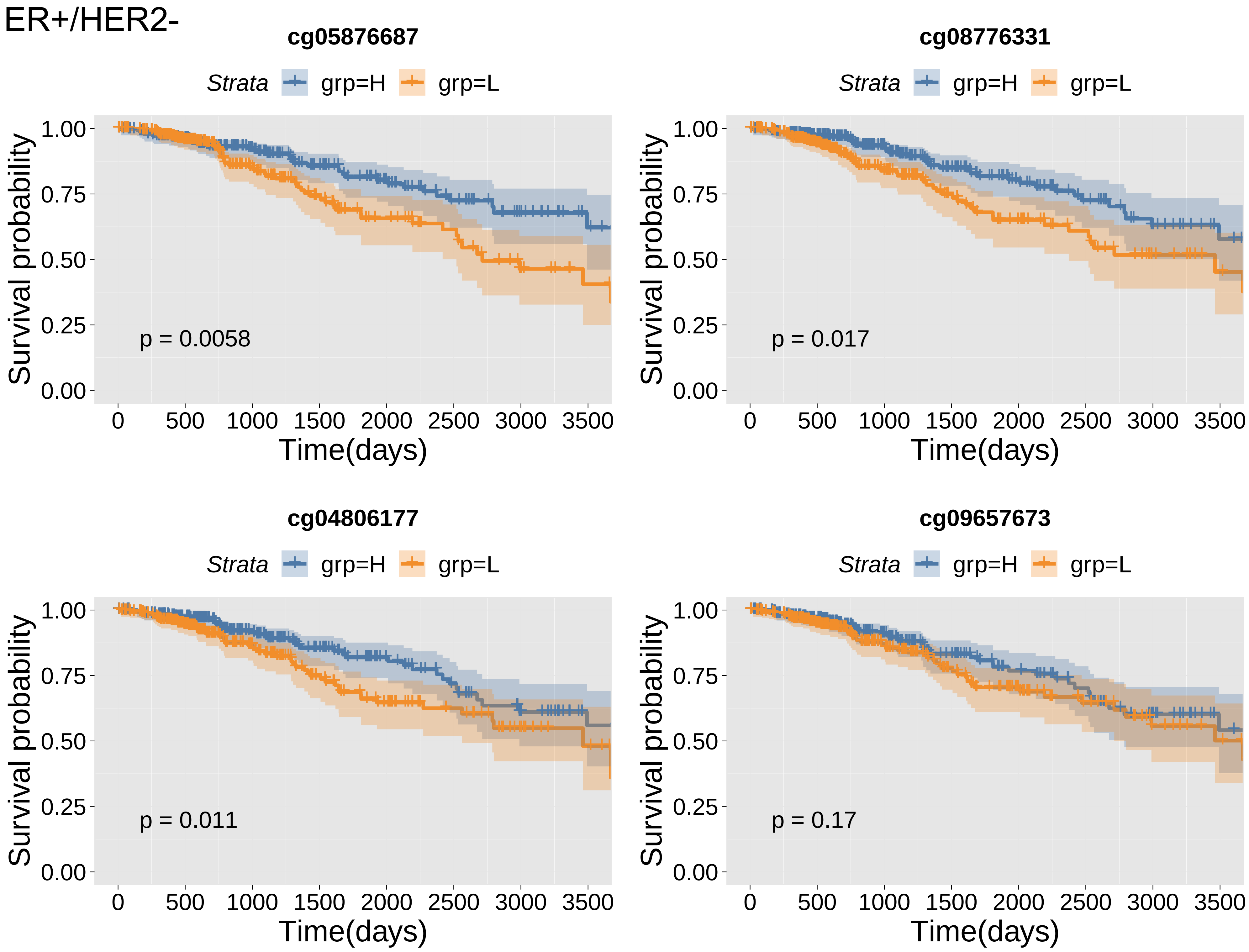

ER+/HER2-
cg05876687
Strata grp=H grp=L
Survival probability
1.00
0.75
0.50
0.25
0.00
p = 0.0058
0 500 1000 1500 2000 2500 3000 3500
Time(days)
cg08776331
Strata grp=H grp=L
Survival probability
1.00
0.75
0.50
0.25
0.00
p = 0.017
0 500 1000 1500 2000 2500 3000 3500
Time(days)
cg04806177
Strata grp=H grp=L
Survival probability
1.00
0.75
0.50
0.25
0.00
p = 0.011
0 500 1000 1500 2000 2500 3000 3500
Time(days)
cg09657673
Strata grp=H grp=L
Survival probability
1.00
0.75
0.50
0.25
0.00
p = 0.17
0 500 1000 1500 2000 2500 3000 3500
Time(days)

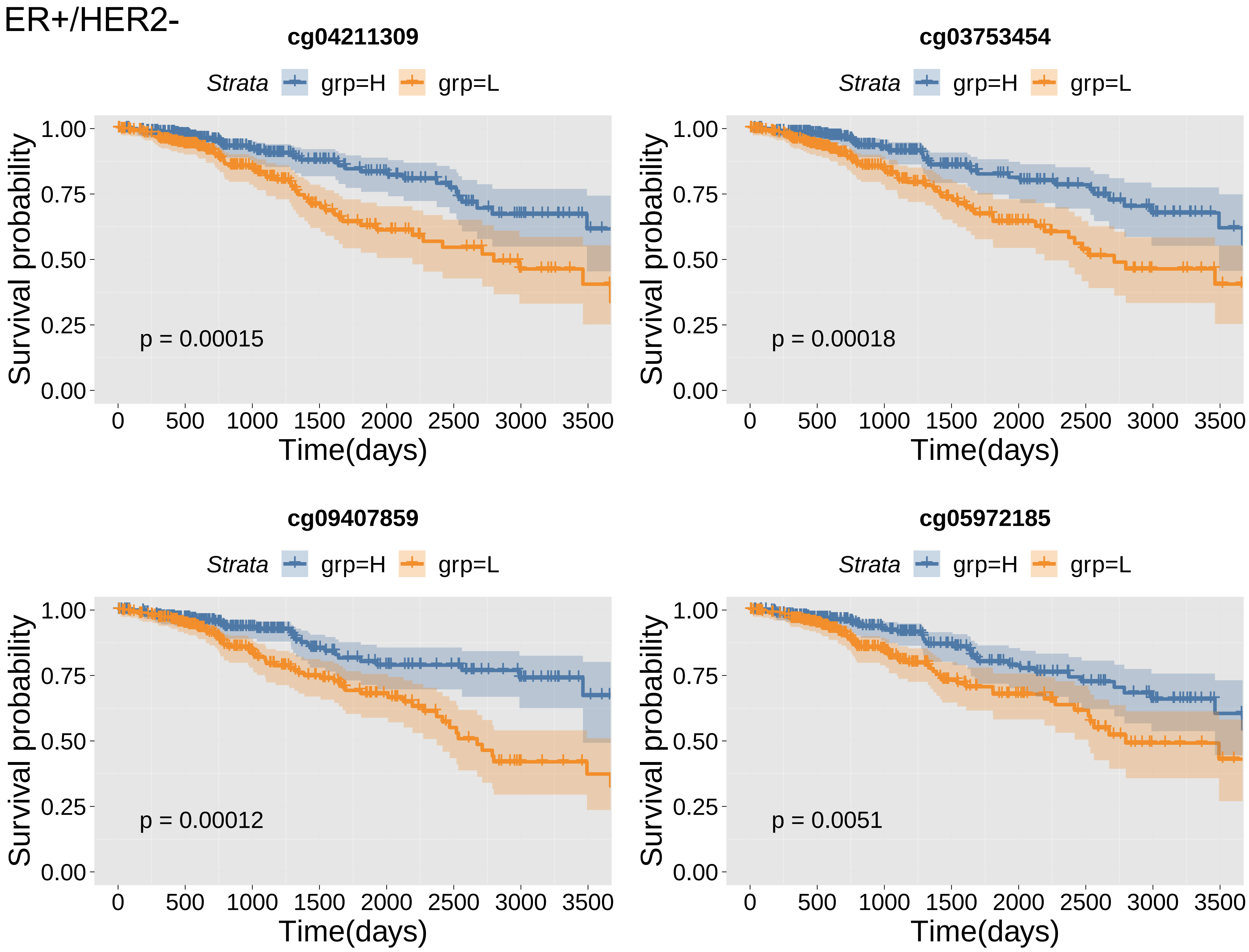

ER+/HER2-
cg04211309
Strata grp=H grp=L
p = 0.00015
cg03753454
Strata grp=H grp=L
p = 0.00018
cg09407859
Strata grp=H grp=L
p = 0.00012
cg05972185
Strata grp=H grp=L
p = 0.0051
Survival probability
Time(days)

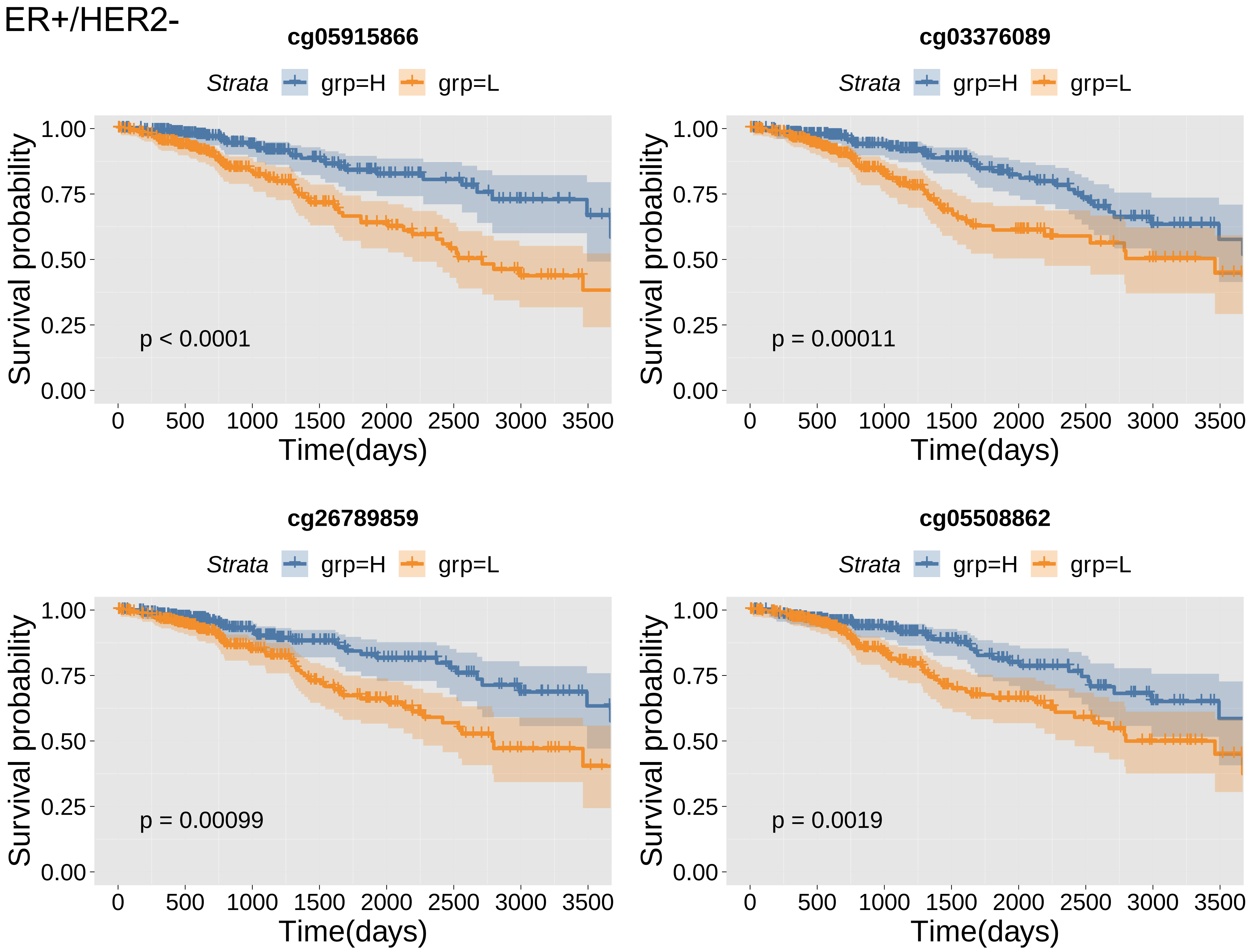
ER+/HER2-
cg05915866
Strata grp=H grp=L
p < 0.0001
cg03376089
Strata grp=H grp=L
p = 0.00011
cg26789859
Strata grp=H grp=L
p = 0.00099
cg05508862
Strata grp=H grp=L
p = 0.0019
Survival probability
1.00
0.75
0.50
0.25
0.00
Time(days)
0
500
1000
1500
2000
2500
3000
3500

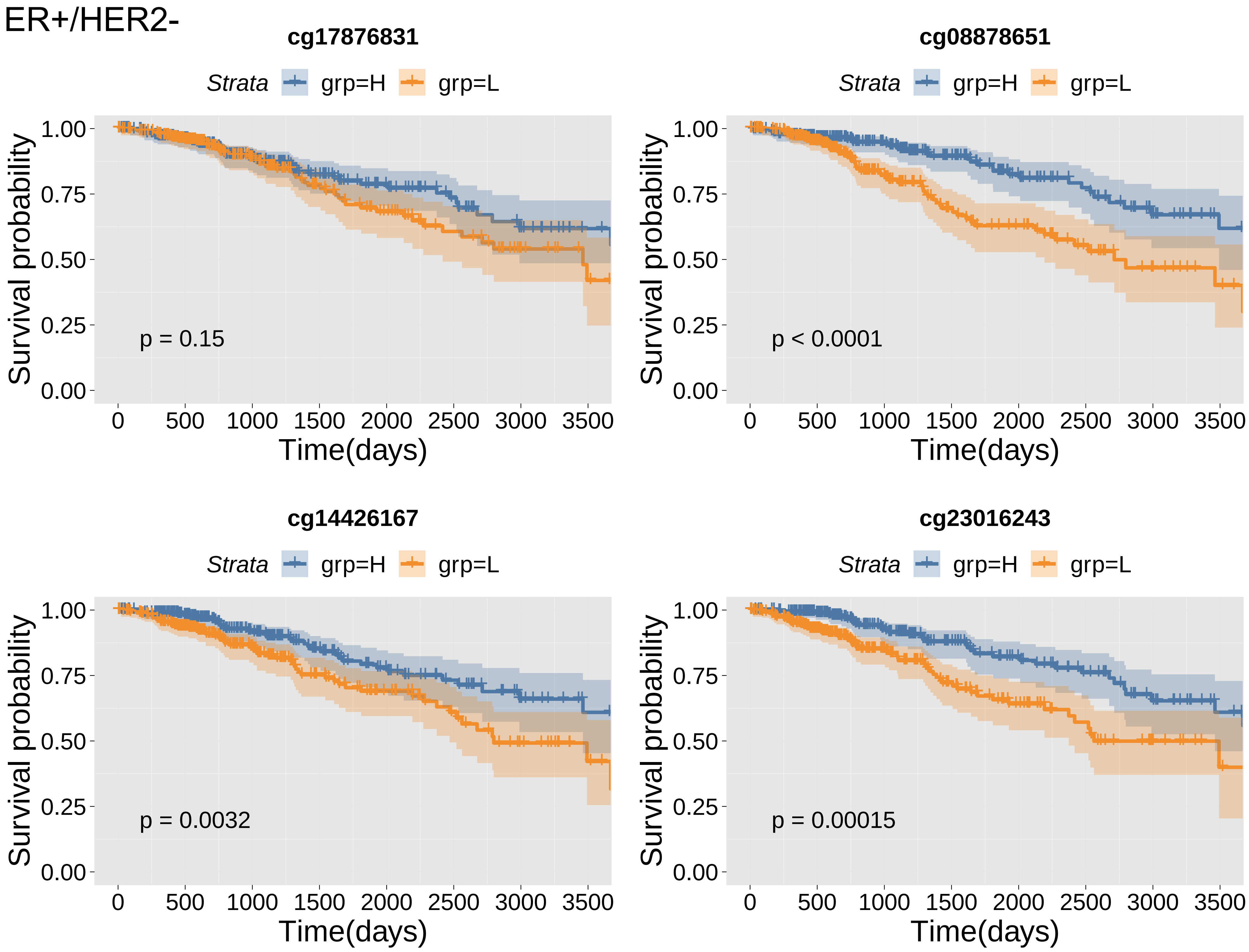

ER+/HER2-
cg17876831
Strata grp=H grp=L
Survival probability
1.00
0.75
0.50
0.25
0.00
p = 0.15
0 500 1000 1500 2000 2500 3000 3500
Time(days)
cg08878651
Strata grp=H grp=L
Survival probability
1.00
0.75
0.50
0.25
0.00
p < 0.0001
0 500 1000 1500 2000 2500 3000 3500
Time(days)
cg14426167
Strata grp=H grp=L
Survival probability
1.00
0.75
0.50
0.25
0.00
p = 0.0032
0 500 1000 1500 2000 2500 3000 3500
Time(days)
cg23016243
Strata grp=H grp=L
Survival probability
1.00
0.75
0.50
0.25
0.00
p = 0.00015
0 500 1000 1500 2000 2500 3000 3500
Time(days)

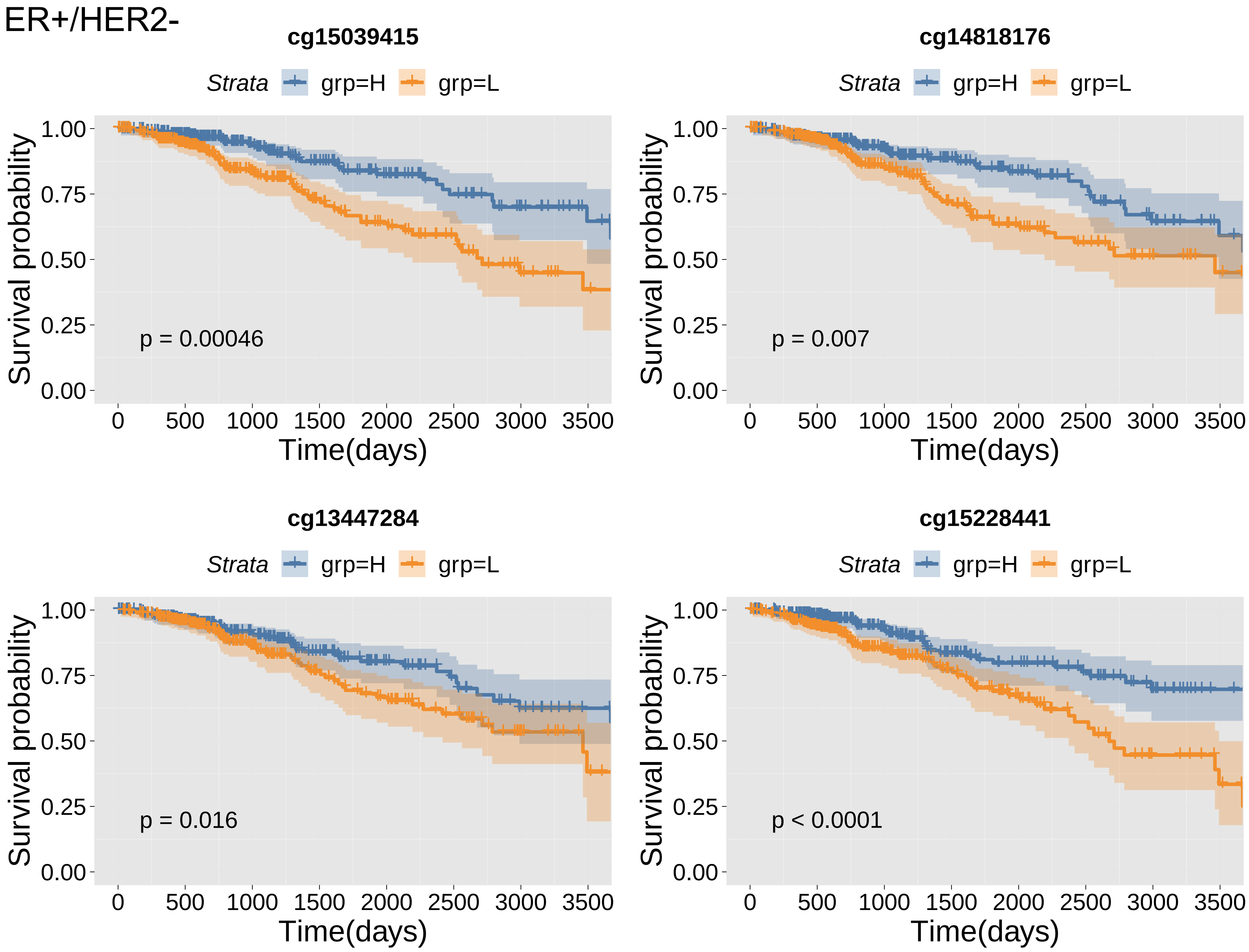
ER+/HER2-
cg15039415
Strata grp=H grp=L
Survival probability
1.00
0.75
0.50
0.25
0.00
p = 0.00046
0 500 1000 1500 2000 2500 3000 3500
Time(days)
cg14818176
Strata grp=H grp=L
Survival probability
p = 0.007
Time(days)
cg13447284
Strata grp=H grp=L
Survival probability
p = 0.016
Time(days)
cg15228441
Strata grp=H grp=L
Survival probability
p < 0.0001
Time(days)

ER+/HER2-

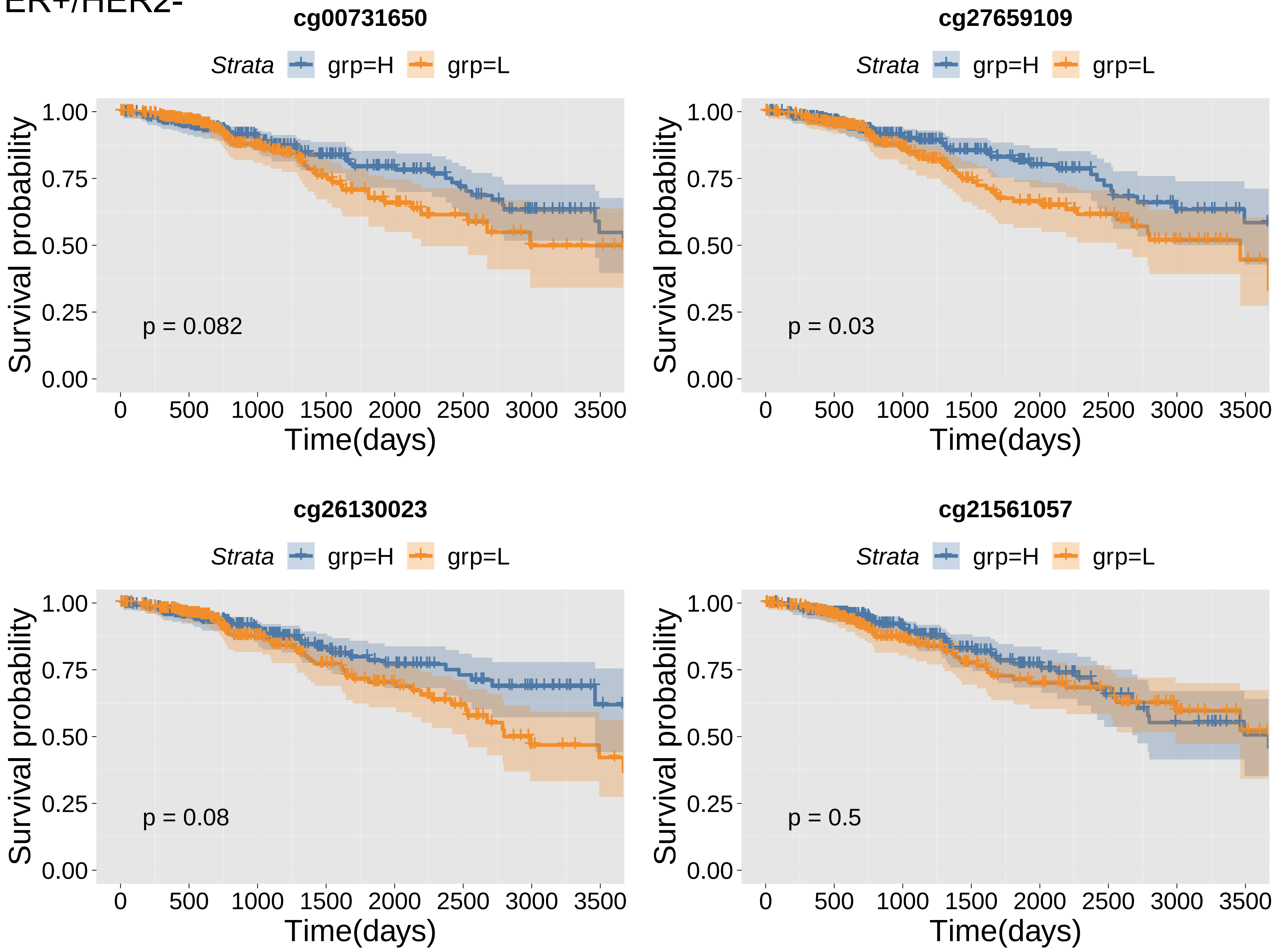
cg00731650
Strata
grp=H
grp=L
Survival probability
1.00
0.75
0.50
0.25
0.00
p = 0.082
0
500
1000
1500
2000
2500
3000
3500
Time(days)
cg27659109
Strata
grp=H
grp=L
Survival probability
p = 0.03
Time(days)
cg26130023
Strata
grp=H
grp=L
Survival probability
p = 0.08
Time(days)
cg21561057
Strata
grp=H
grp=L
Survival probability
p = 0.5
Time(days)

ER+/HER2-

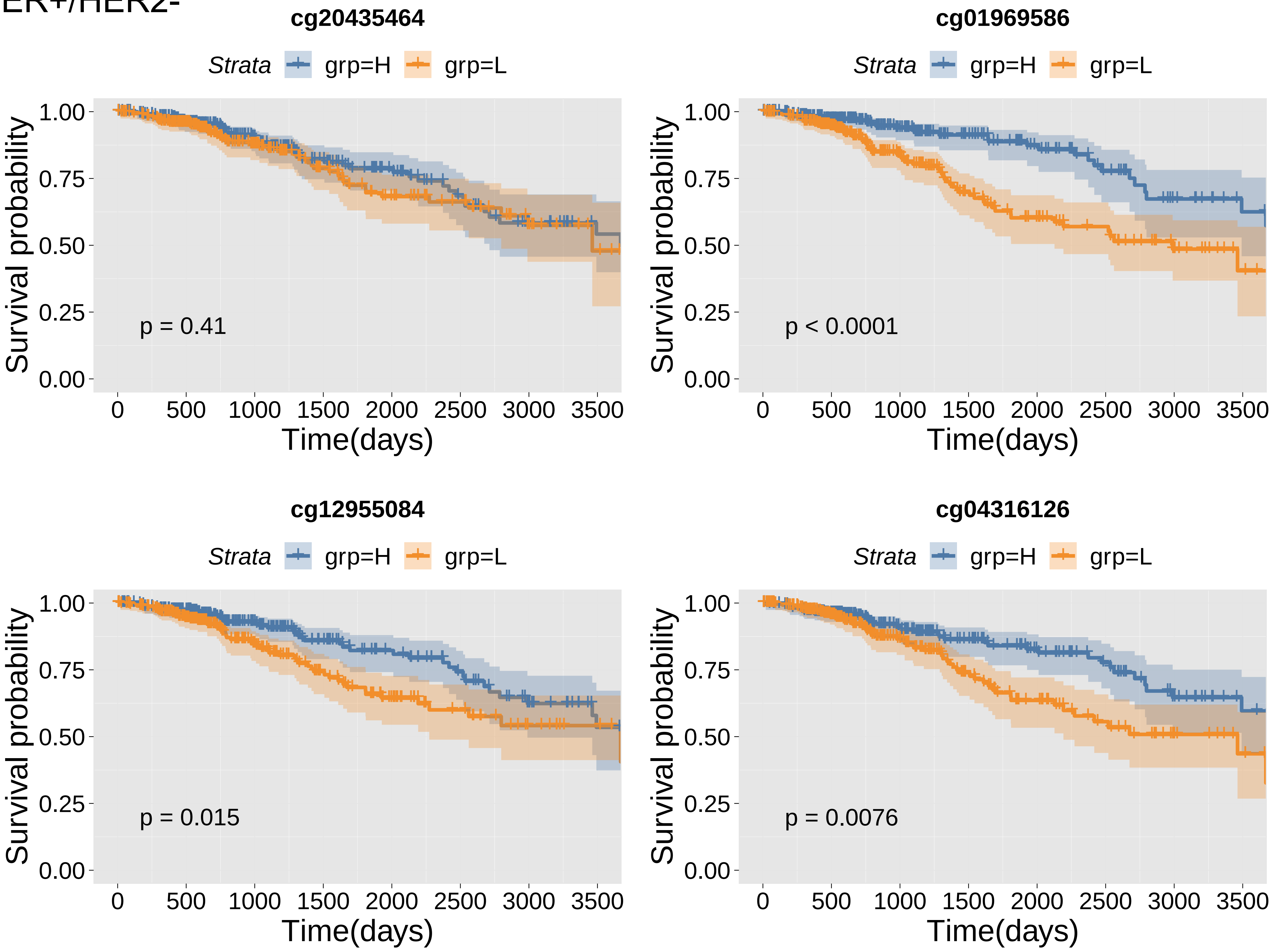

cg20435464
Strata grp=H grp=L
Survival probability
1.00
0.75
0.50
0.25
0.00
p = 0.41
0 500 1000 1500 2000 2500 3000 3500
Time(days)
cg01969586
Strata grp=H grp=L
Survival probability
1.00
0.75
0.50
0.25
0.00
p < 0.0001
0 500 1000 1500 2000 2500 3000 3500
Time(days)
cg12955084
Strata grp=H grp=L
Survival probability
1.00
0.75
0.50
0.25
0.00
p = 0.015
0 500 1000 1500 2000 2500 3000 3500
Time(days)
cg04316126
Strata grp=H grp=L
Survival probability
1.00
0.75
0.50
0.25
0.00
p = 0.0076
0 500 1000 1500 2000 2500 3000 3500
Time(days)

ER+/HER2-

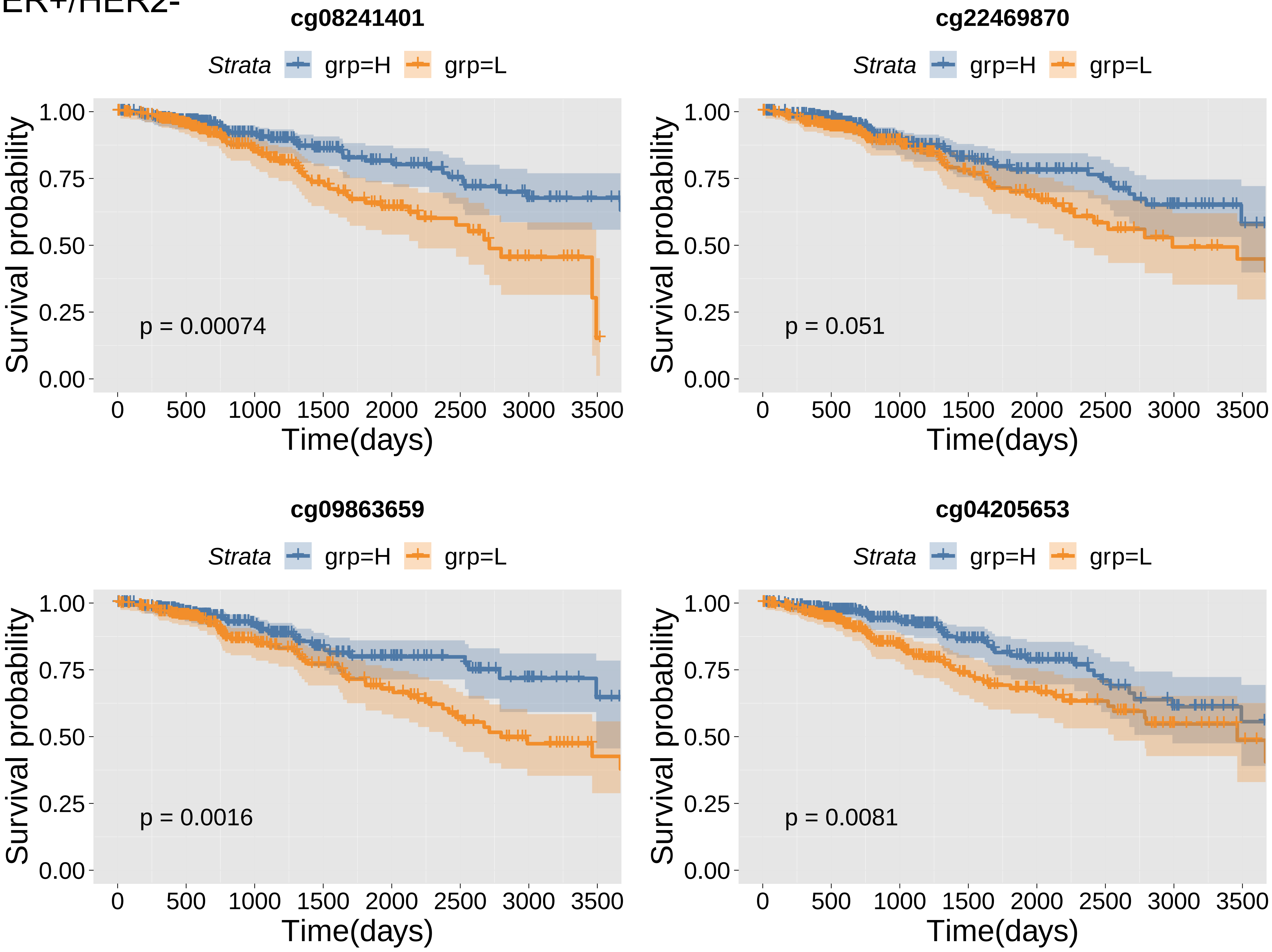
cg08241401
Strata grp=H grp=L
Survival probability
1.00
0.75
0.50
0.25
0.00
p = 0.00074
0 500 1000 1500 2000 2500 3000 3500
Time(days)
cg22469870
Strata grp=H grp=L
Survival probability
p = 0.051
Time(days)
cg09863659
Strata grp=H grp=L
Survival probability
p = 0.0016
Time(days)
cg04205653
Strata grp=H grp=L
Survival probability
p = 0.0081
Time(days)

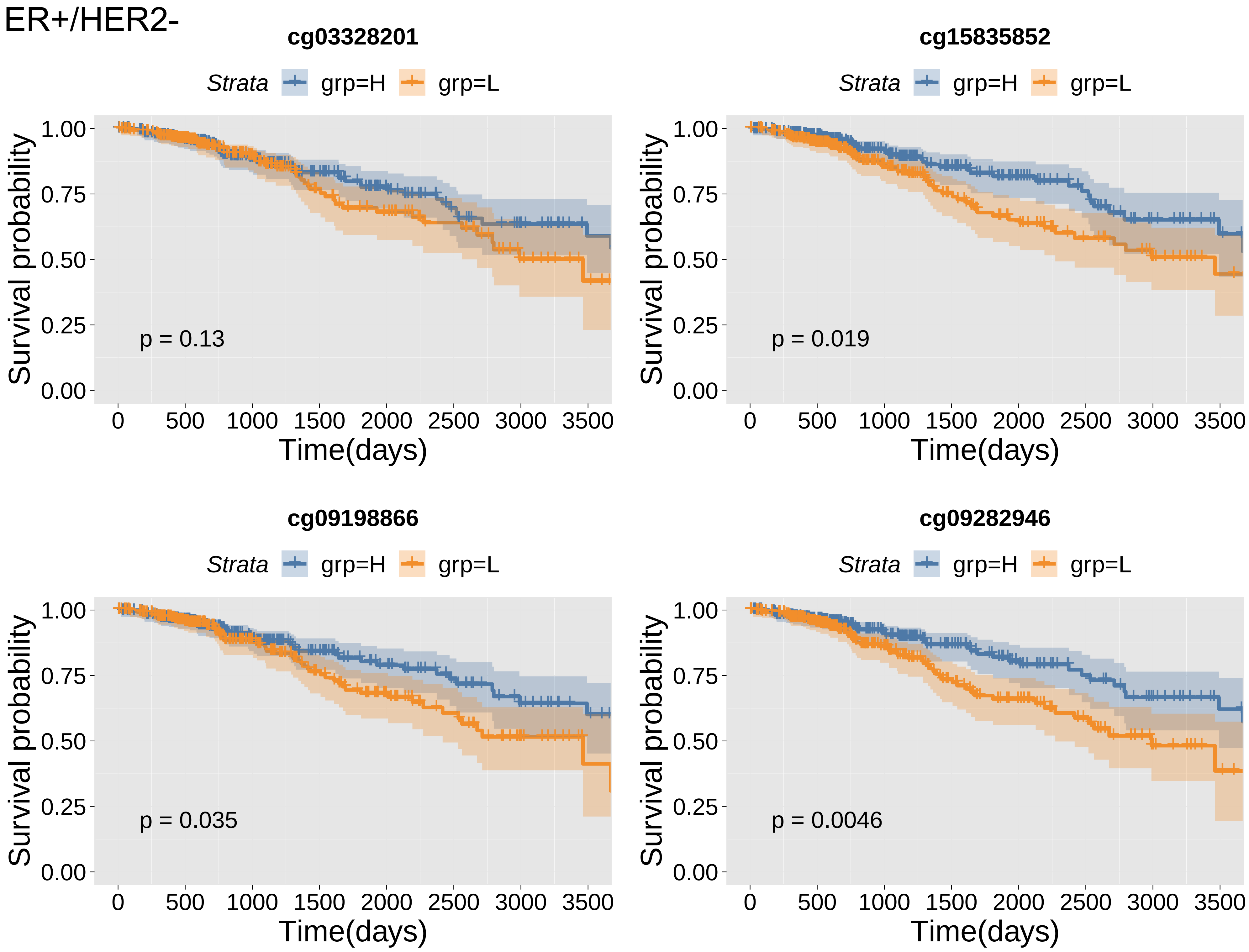

ER+/HER2-
cg03328201
Strata grp=H grp=L
Survival probability
1.00
0.75
0.50
0.25
0.00
p = 0.13
0 500 1000 1500 2000 2500 3000 3500
Time(days)
cg15835852
Strata grp=H grp=L
Survival probability
p = 0.019
Time(days)
cg09198866
Strata grp=H grp=L
Survival probability
p = 0.035
Time(days)
cg09282946
Strata grp=H grp=L
Survival probability
p = 0.0046
Time(days)

ER+/HER2-

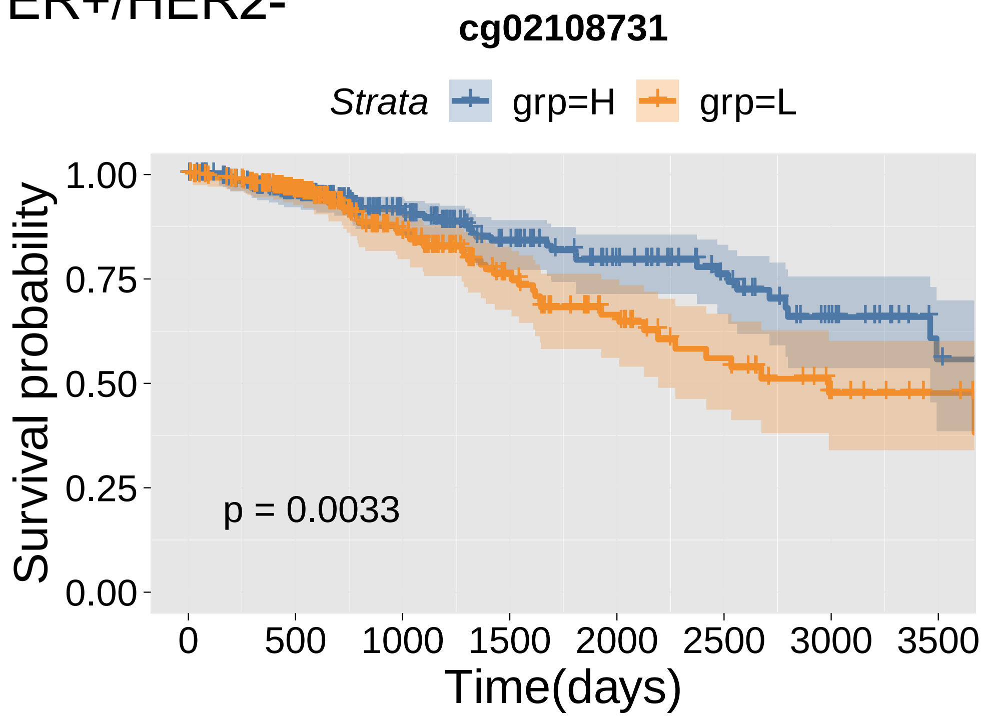

cg02108731
Strata
grp=H
grp=L
Survival probability
1.00
0.75
0.50
0.25
0.00
p = 0.0033
0
500
1000
1500
2000
2500
3000
3500
Time(days)

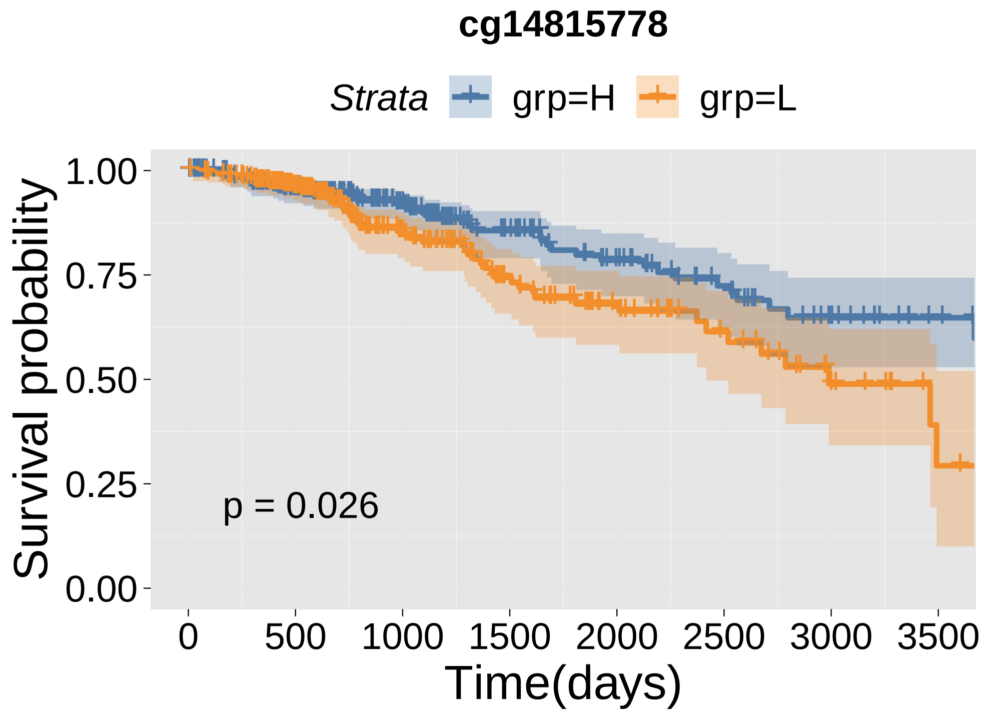

cg14815778
Strata
grp=H
grp=L
Survival probability
1.00
0.75
0.50
0.25
0.00
p = 0.026
0
500
1000
1500
2000
2500
3000
3500
Time(days)

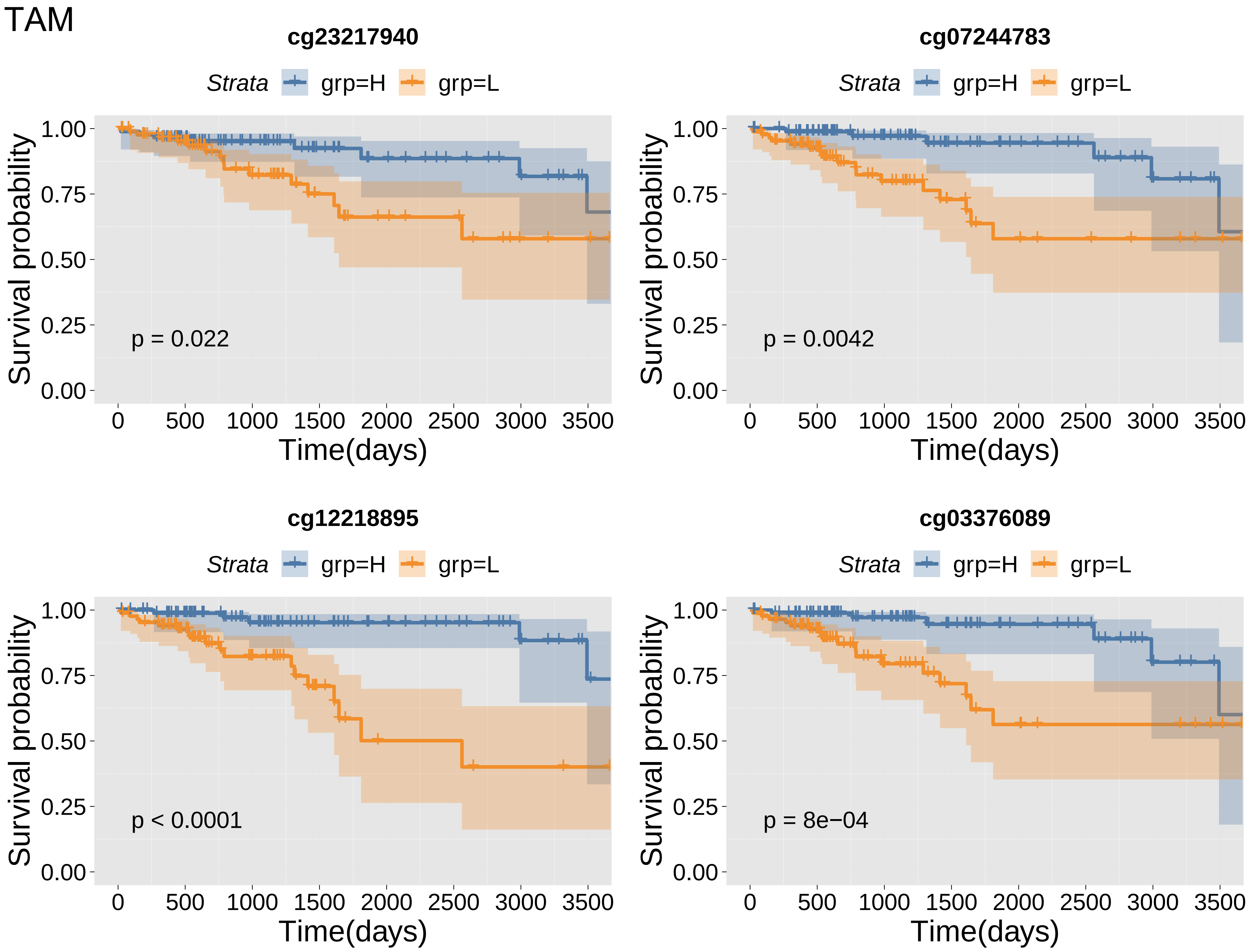

TAM
cg23217940
Strata grp=H grp=L
Survival probability
1.00
0.75
0.50
0.25
0.00
p = 0.022
0 500 1000 1500 2000 2500 3000 3500
Time(days)
cg07244783
Strata grp=H grp=L
p = 0.0042
cg12218895
Strata grp=H grp=L
p < 0.0001
cg03376089
Strata grp=H grp=L
p = 8e−04

TAM

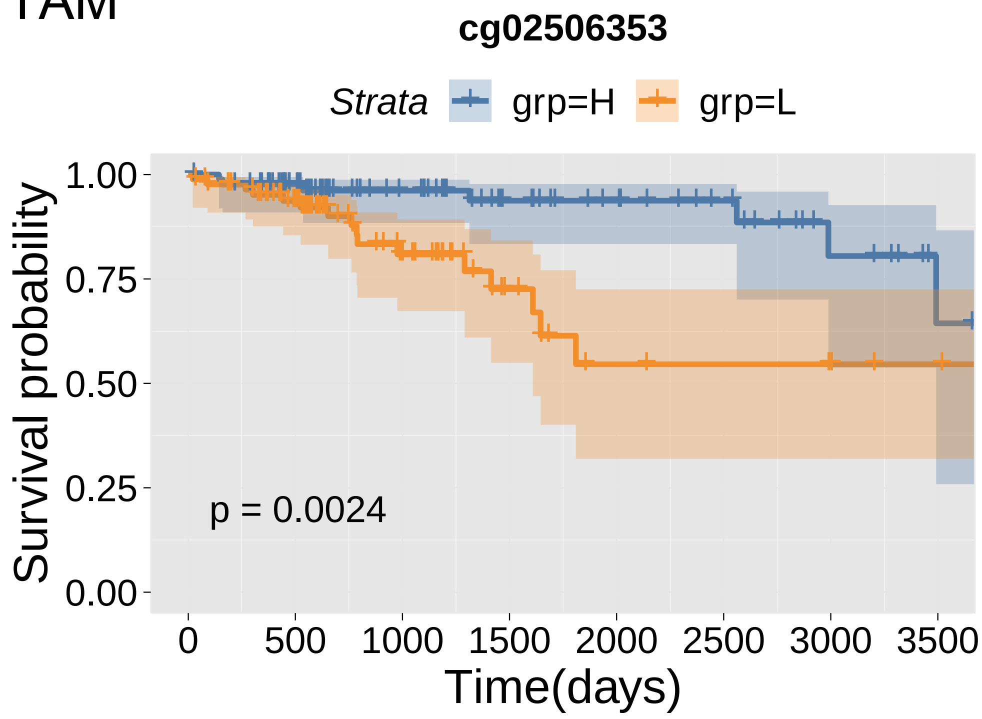
cg02506353
Strata
grp=H
grp=L
Survival probability
1.00
0.75
0.50
0.25
0.00
p = 0.0024
0
500
1000
1500
2000
2500
3000
3500
Time(days)

AI

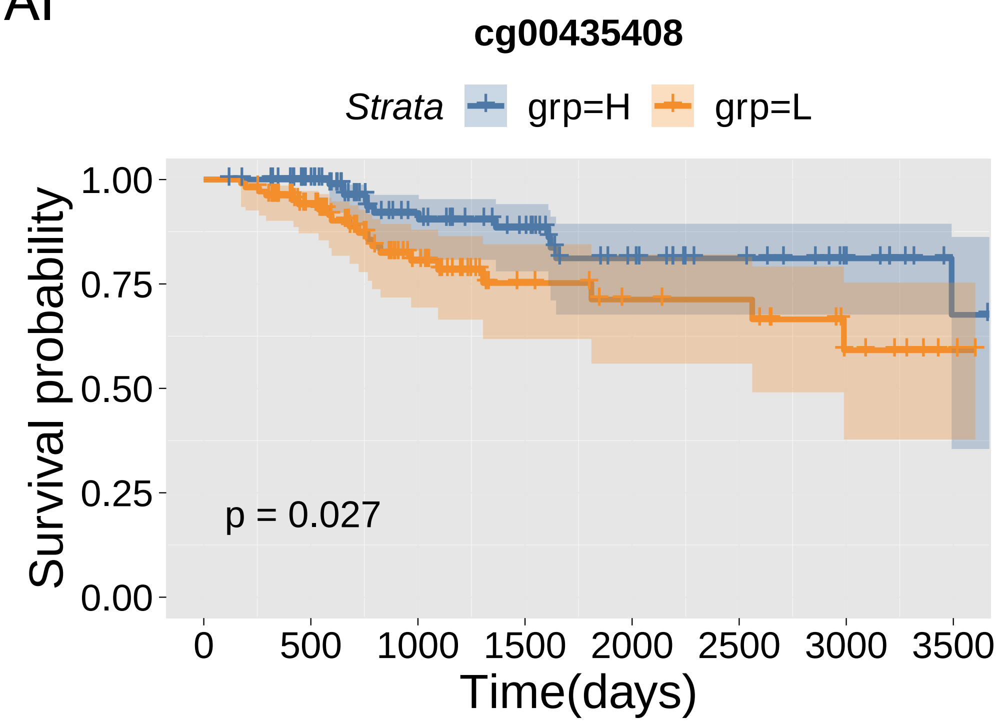

cg00435408
Strata
grp=H
grp=L
1.00
0.75
0.50
0.25
0.00
Survival probability
p = 0.027
0
500
1000
1500
2000
2500
3000
3500
Time(days)
