# Supplementary material for: Candidate methylation sites associated with endocrine therapy resistance in ER+/HER2- breast cancer
Source: BMC Cancer. 2020 Jul 19;20:676. doi: 10.1186/s12885-020-07100-z (PMC7368985; doi:10.1186/s12885-020-07100-z)
Supplement: Supplementary file 6 — Additional file 6. Single-locus Venn diagram. Venn diagram of the single-locus signatures in the ER+/HER2-, TAM and AI cohorts. [file 12885_2020_7100_MOESM6_ESM.pptx]

## Slide 1
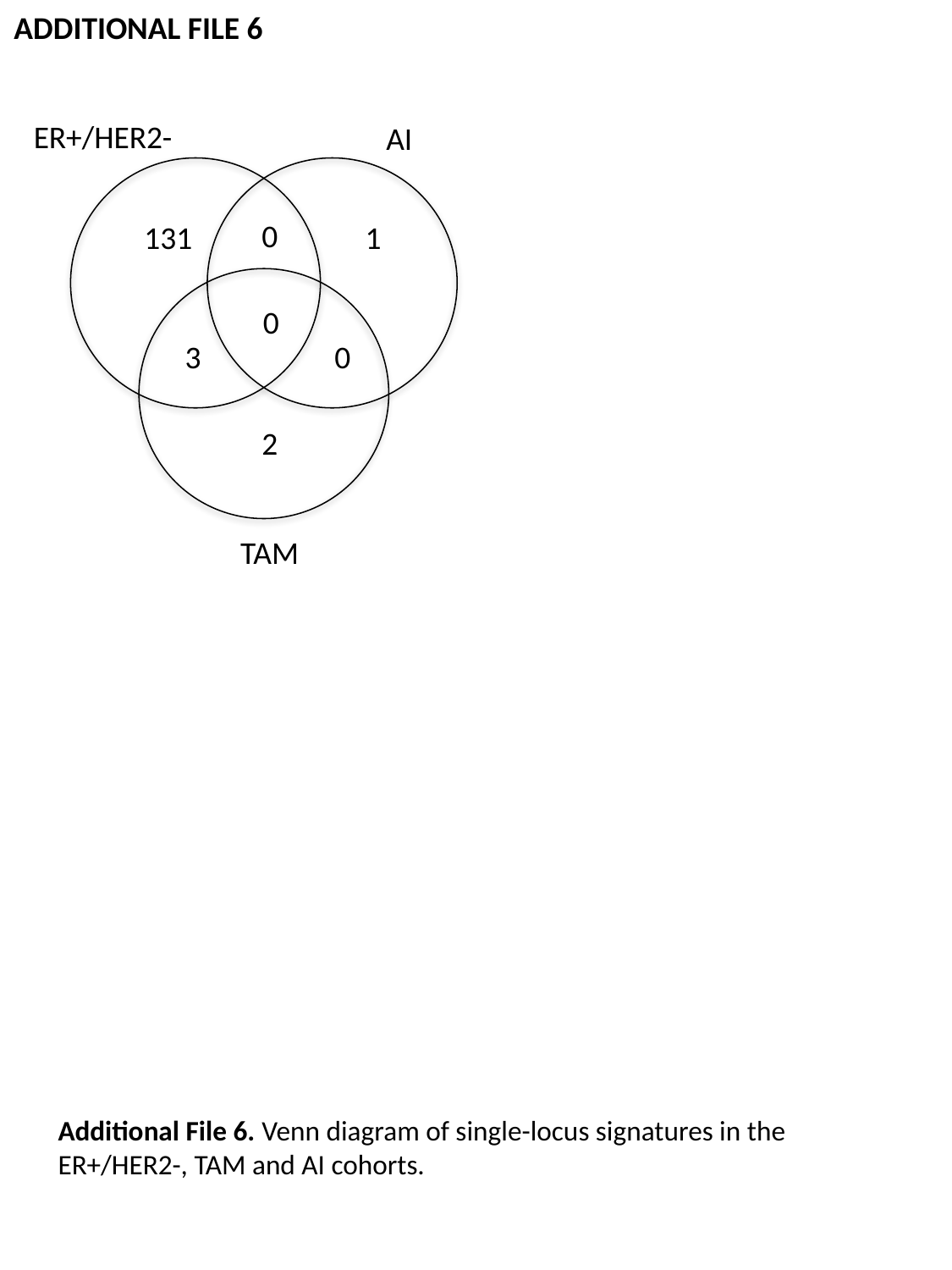

ADDITIONAL FILE 6
ER+/HER2-
AI
0
131
1
0
3
0
2
TAM
Additional File 6. Venn diagram of single-locus signatures in the ER+/HER2-, TAM and AI cohorts.
