# Supplementary material for: Candidate methylation sites associated with endocrine therapy resistance in ER+/HER2- breast cancer
Source: BMC Cancer. 2020 Jul 19;20:676. doi: 10.1186/s12885-020-07100-z (PMC7368985; doi:10.1186/s12885-020-07100-z)
Supplement: Supplementary file 9 — Additional file 9. Overlap between single-locus and multi-locus signatures. Venn diagrams of the overlap between single-locus and multi-locus signatures in the three cohorts ER+/HER2-, TAM and AI. (PPTX 41 kb) [file 12885_2020_7100_MOESM9_ESM.pptx]

## Slide 1
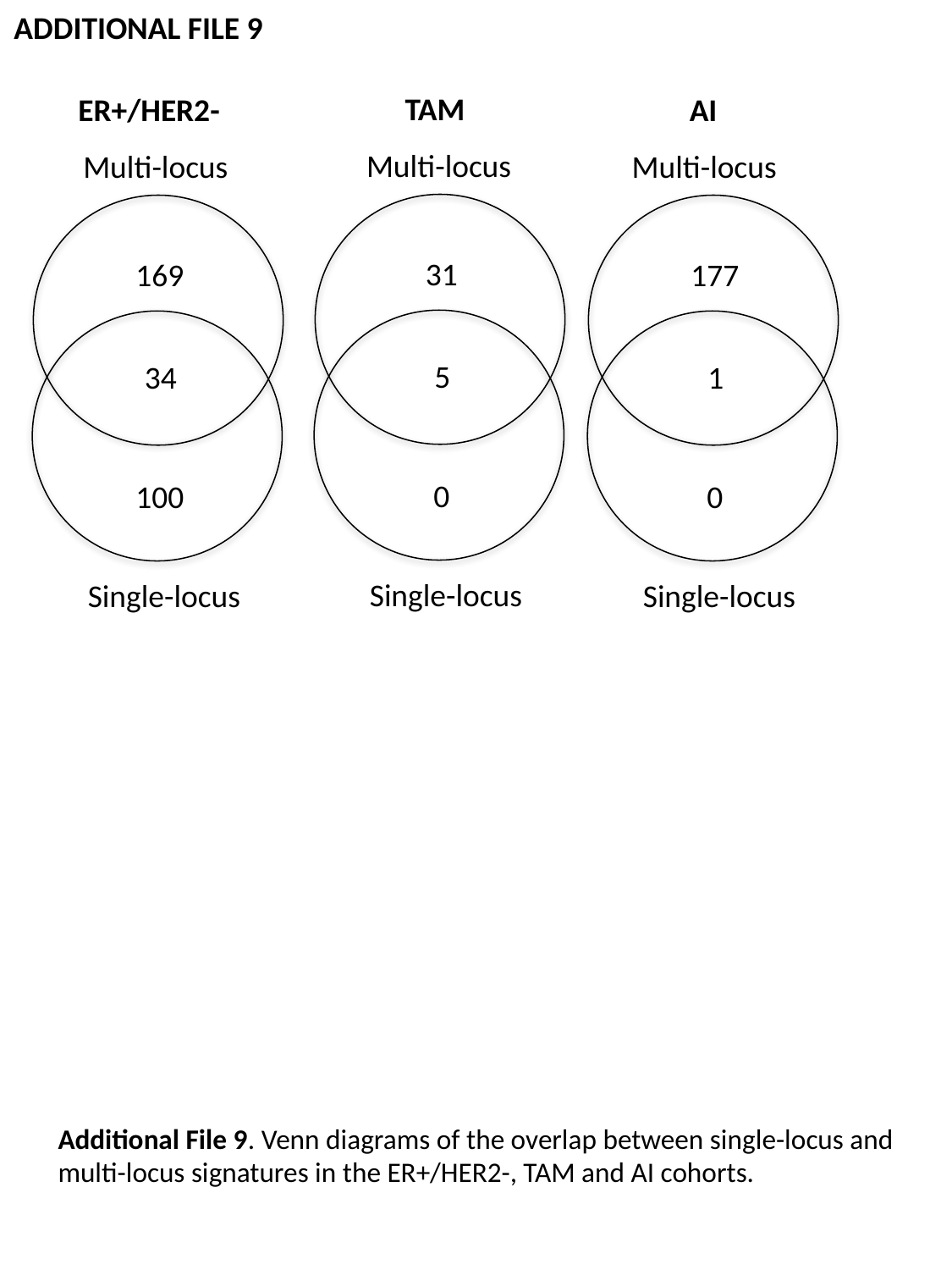

ADDITIONAL FILE 9
TAM
ER+/HER2-
AI
Multi-locus
Multi-locus
Multi-locus
31
169
177
5
34
1
0
100
0
Single-locus
Single-locus
Single-locus
Additional File 9. Venn diagrams of the overlap between single-locus and multi-locus signatures in the ER+/HER2-, TAM and AI cohorts.
