# Supplementary material for: Candidate methylation sites associated with endocrine therapy resistance in ER+/HER2- breast cancer
Source: BMC Cancer. 2020 Jul 19;20:676. doi: 10.1186/s12885-020-07100-z (PMC7368985; doi:10.1186/s12885-020-07100-z)
Supplement: Supplementary file 11 — Additional file 11. qRT-PCR results. Primer sequences and gene expression levels of CD36, FGF12, HDAC9, and KRT4 determined by qRT-PCR after treatment with tamoxifen or long-term estrogen deprivation relative to their expression in untreated T47D cells. [file 12885_2020_7100_MOESM11_ESM.docx]

**ADDITIONAL FILE 11**

**Table**. Primer sequences used for qRT-PCR analyses.

| **Gene** | **Primer 1** | **Primer 2** |
| --- | --- | --- |
| *CD36* | tggaacagaggctgacaactt | ttgattttgatagatatgggatgc |
| *FGF12* | tgctgatctttctttttcagga | aagctcaatacatcattctgaacatt |
| *HDAC9* | aatgcacagtatgatcagctcag | ggtctgtccttaggtctaaaggt |
| *KRT4* | ggggctccttcagtggtaa | ctggttgatggtgacctcct |

**Figure**. Gene expression levels of selected genes determined by qRT-PCR in T47D cells treated with tamoxifen (TMX) or long-term estrogen deprived (LTED) relative to their expression in untreated T47D cells (WT). Data are shown as mean and standard error of the mean for three independent experiments. Statistical analyses were performed by unpaired two-tailed Student’s t-test *** p-value <0.001, ** p-value <0.01, * p-value <0.05.
