# Supplementary figures and images for: Candidate methylation sites associated with endocrine therapy resistance in ER+/HER2- breast cancer
Source: BMC Cancer. 2020 Jul 19;20:676. doi: 10.1186/s12885-020-07100-z (PMC7368985; doi:10.1186/s12885-020-07100-z)

A

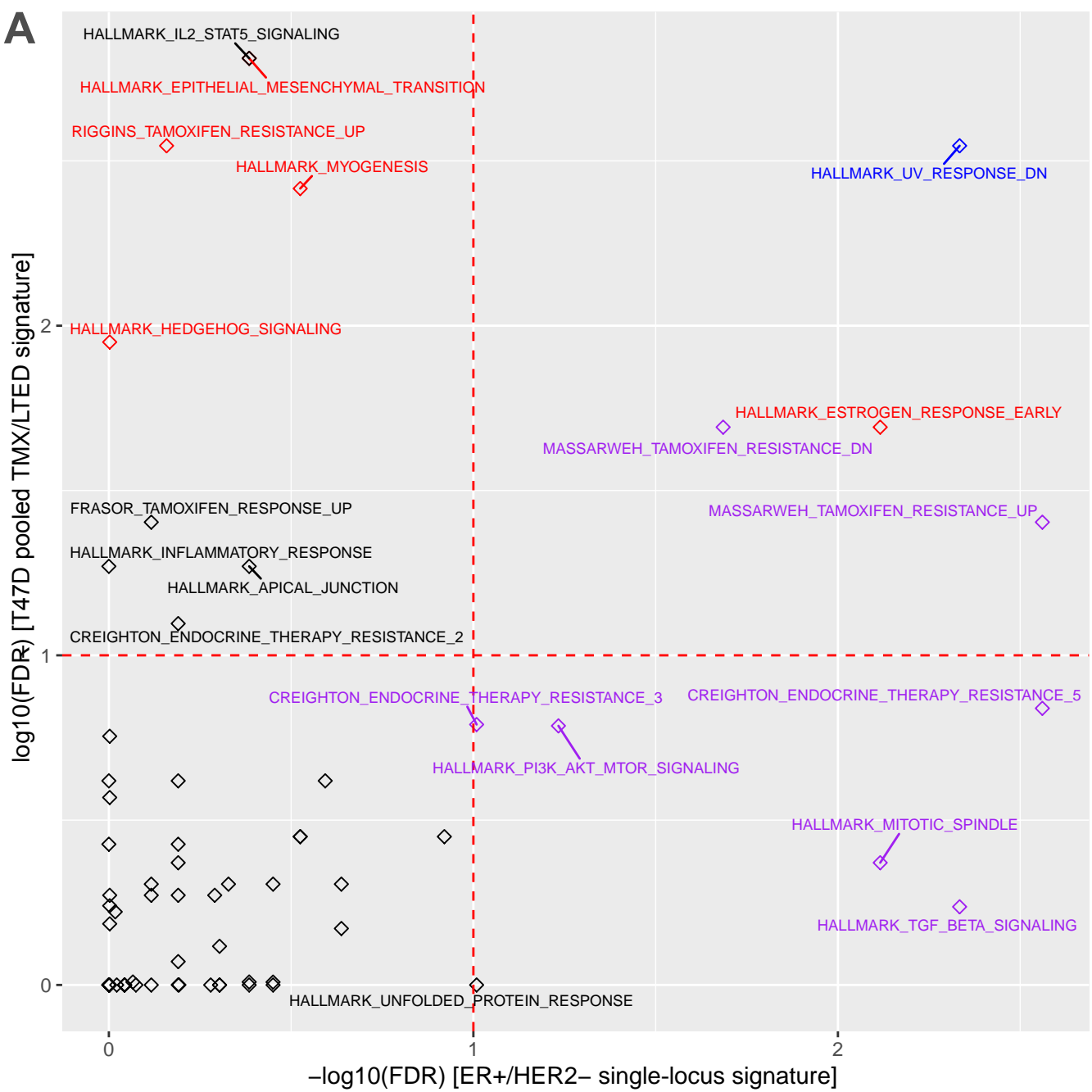

**B**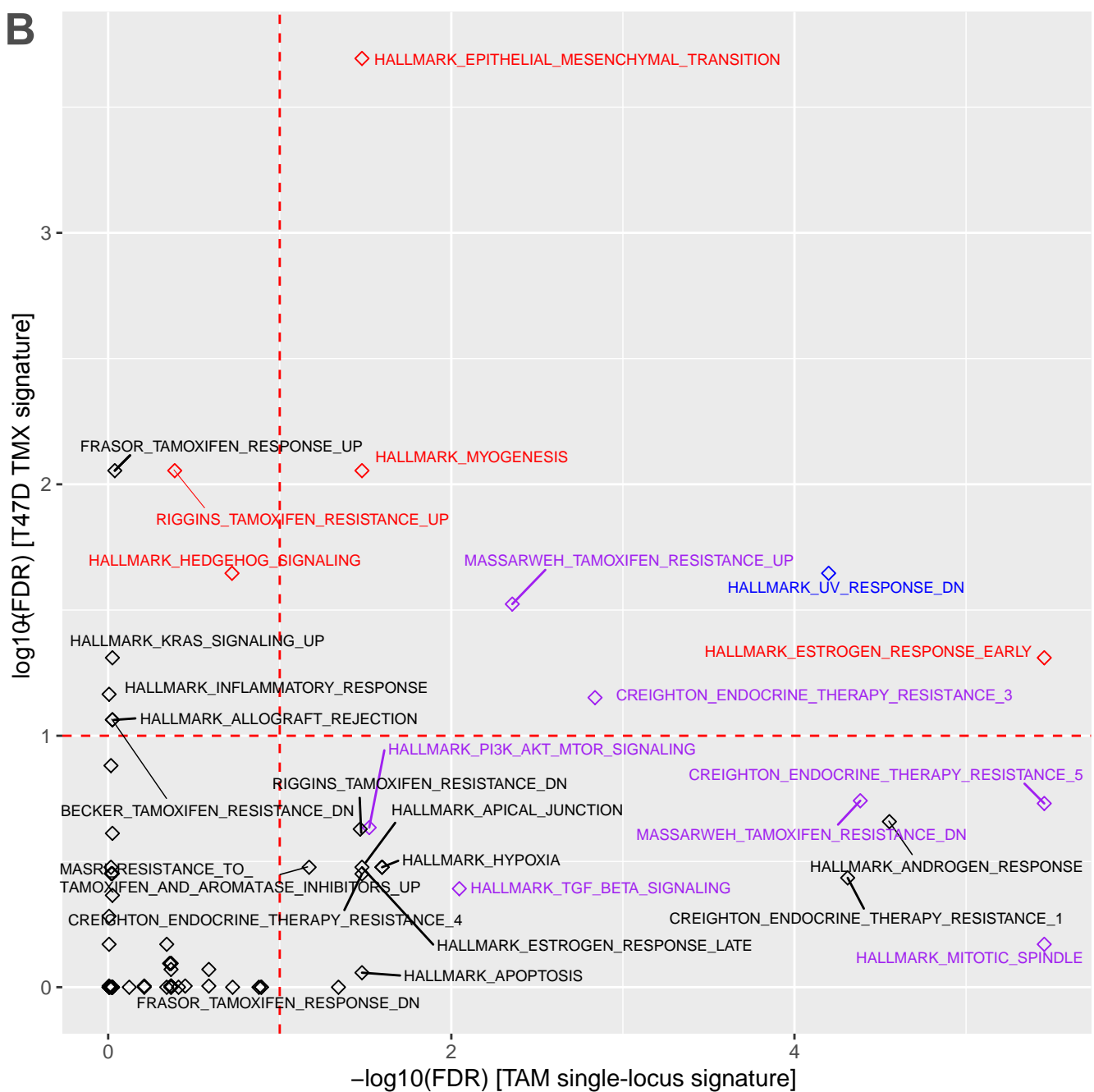

C

log10(FDR) [T47D LTED signature]

-log10(FDR) [AI single-locus signature]

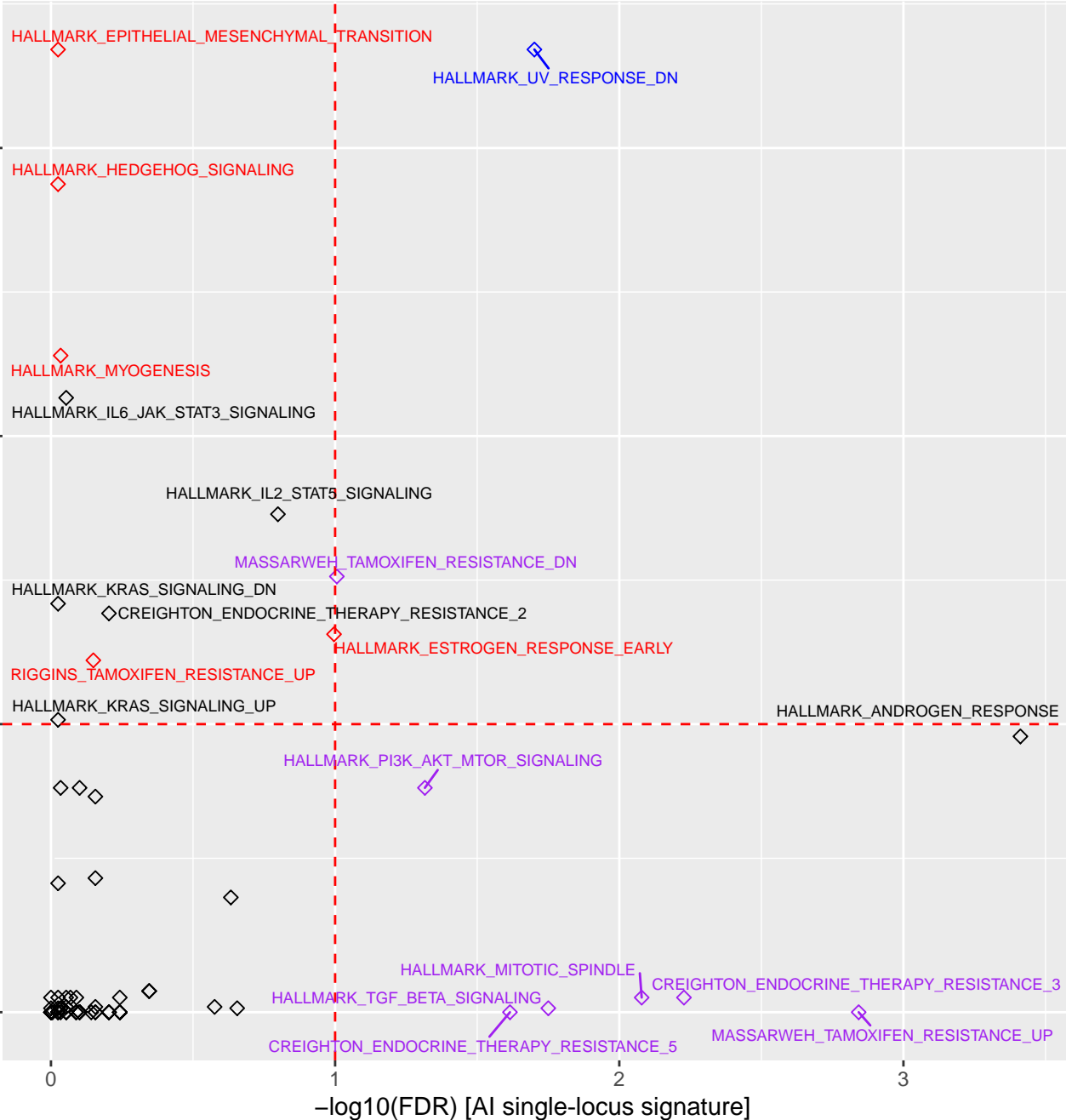

Supplement: Supplementary file 12 — Additional file 12. Gene set enrichment analysis. Gene set enrichment analysis of single-locus survival (x-axis) and RA signatures (y-axis). (A) T47D TMX/LTED signature versus ER+/HER2− single-locus signature. (B) T47D TMX signatuare versus TAM single-locus signature. (C) T47D LTED signature versus AI single-locus signature. Each diamond represents either a Hallmark gene set or a curated gene set related to tamoxifen treatment or ET from the Molecular Signatures Database. Gene sets significantly enriched (FDR < 0.1, that is -log10(FDR) > 1, indicated by the red dashed lines) in at least one of the two signatures are labelled with their name. Purple: gene sets that are significantly enriched in all three survival signatures. Red: gene sets that are significantly enriched in all three RA signatures. Blue: gene sets that are significantly enriched in all six signatures. [file 12885_2020_7100_MOESM12_ESM.pdf]

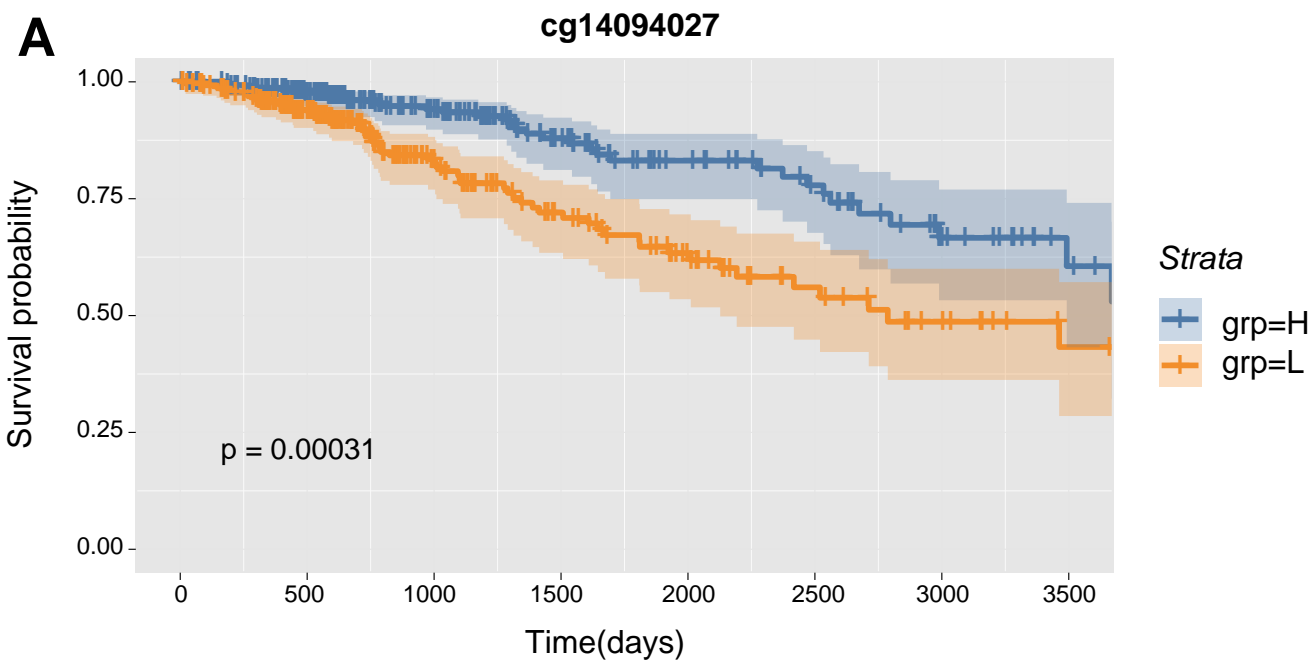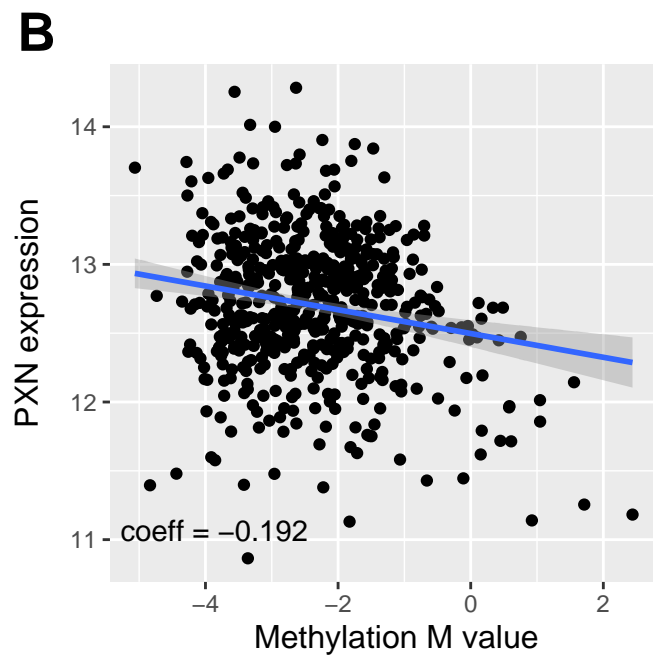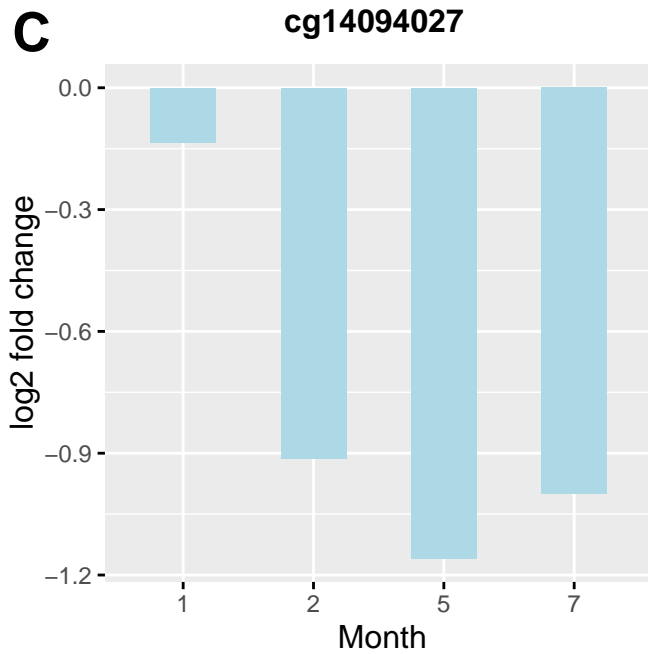

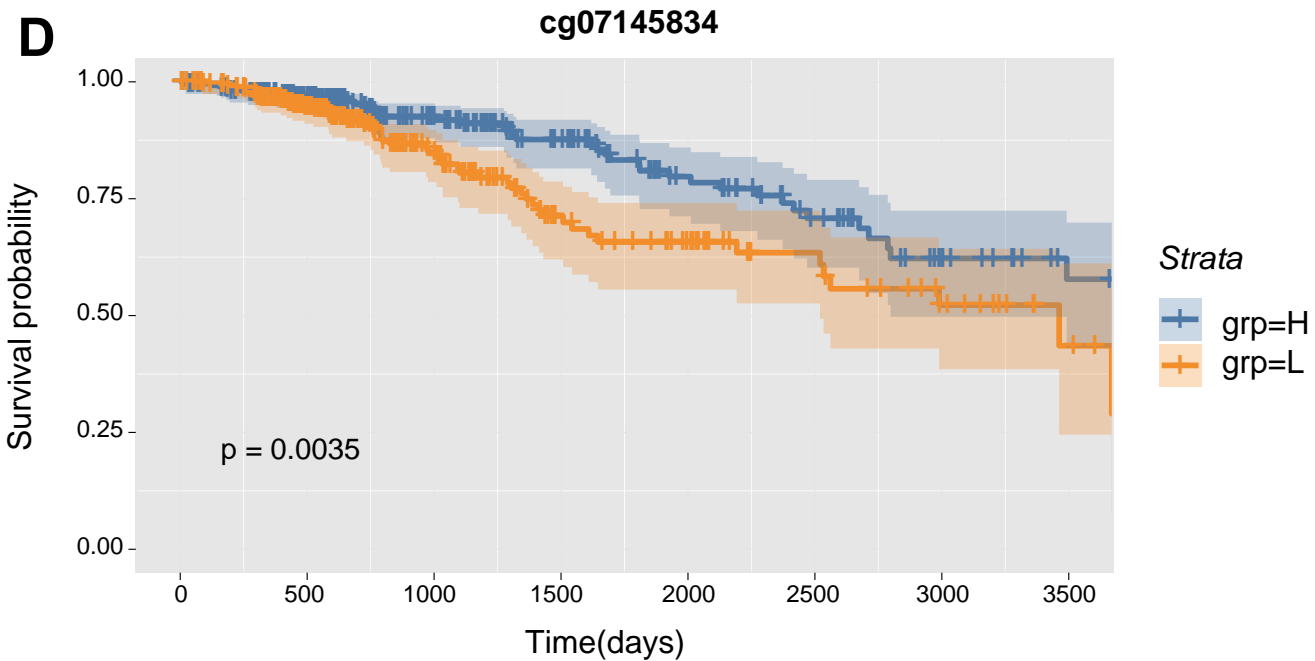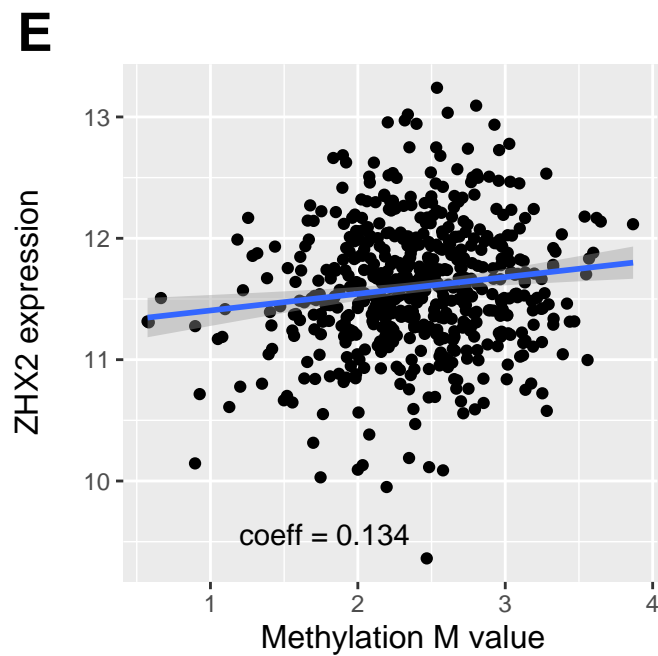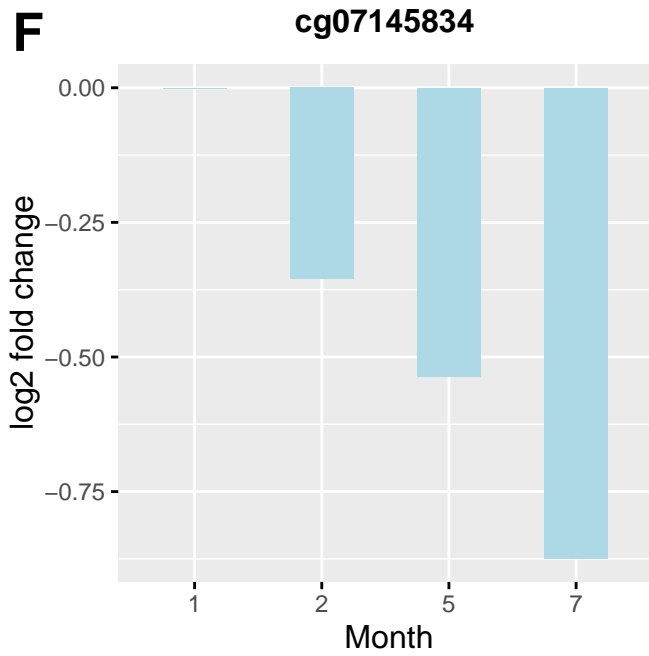

Supplement: Supplementary file 15 — Additional file 15. Association of methylation levels of selected CpG sites with survival and resistance acquisition and their correlation with expression levels of the associated genes. (A,D) Kaplan-Meier plot for CpG site cg14094027 located in the gene body of PXN (A) and CpG site cg07145834 located in the 5’UTR of ZHX2 (D), both significantly associated with survival in the ER+/HER2- cohort. Patients were stratified based on methylation levels. H, methylation levels above median; L, methylation levels below median. Shaded areas in the Kaplan-Meier plot denote the 95% CI in the H and L strata. P-values are based on a log-rank test. (B,E) Correlation between paired DNA methylation and gene expression profiles (B: cg14094027, PXN; E: cg07145834, ZHX2). Each circle corresponds to a patient sample in the ER+/HER2- cohort. The Pearson correlation coefficient is indicated, together with the corresponding regression line and its 95% CI. (C,F) Log2-fold change of the methylation M-values of cg14094027 (C) and cg07145834 (F) in the comparison of T47D TMX/LTED versus WT. [file 12885_2020_7100_MOESM15_ESM.pdf]
